# GAFF/IPolQ-Mod+LJ-Fit: Optimized force field parameters for solvation free energy predictions

**DOI:** 10.5599/admet.837

**Published:** 2020-06-28

**Authors:** Andreas Mecklenfeld, Gabriele Raabe

**Affiliations:** 1 Institut für Thermodynamik, Technische Universität Braunschweig, Hans-Sommer Strasse 5, 38106 Braunschweig, Germany; 2 Center of Pharmaceutical Engineering, Technische Universität Braunschweig, Franz-Liszt-Strasse 35a, 38106 Braunschweig, Germany

**Keywords:** Molecular dynamics simulations, force field optimization, solvation free energies, relative solubilities

## Abstract

Rational drug design featuring explicit solubility considerations can greatly benefit from molecular dynamics simulations, as they allow for the prediction of the Gibbs free energy of solvation and thus relative solubilities. In our previous work (A. Mecklenfeld, G. Raabe. J. Chem. Theory Comput. **13** no. 12 (2017) 6266–6274), we have compared solvation free energy results obtained with the General Amber Force Field (GAFF) and its default restrained electrostatic potential (RESP) partial charges to those obtained by modified implicitly polarized charges (IPolQ-Mod) for an implicit representation of impactful polarization effects. In this work, we have adapted Lennard-Jones parameters for GAFF atom types in combination with IPolQ-Mod to further improve the accuracies of solvation free energy and liquid density predictions. We thereby focus on prominent atom types in common drugs. For the refitting, 357 respectively 384 systems were considered for free energies and densities and validation was performed for 142 free energies and 100 densities of binary mixtures. By the in-depth comparison of simulation results for default GAFF, GAFF with IPolQ-Mod and our new set of parameters, which we label GAFF/IPolQ-Mod+LJ-Fit, we can clearly highlight the improvements of our new model for the description of both relative solubilities and fluid phase behaviour.

## Introduction

Solubility is a crucial thermophysical property in the pharmaceutical industry [1] and its assessment is therefore of utmost importance. Molecular simulations can be interpreted as experiments on the computer and are capable to complement the computer-aided drug design. They allow for the accurate prediction of thermophysical properties to determine suitable solvents for an active pharmaceutical ingredient in an early stage of the drug development process. According to Liu *et al*. [2], the relative solubilities can be determined by Eq. (1):


(1)

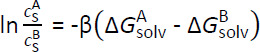



in which *c*_S_ is the molar concentration of solute S in solvent A or B, β is the inverse temperature and Δ*G*_solv_ is the Gibbs free energy of solvation of the solute. The Gibbs free energy of solvation corresponds to the change in the Gibbs free energy for transferring a single solute from a vacuum into a condensed phase [3]. In the vacuum phase, no intermolecular interactions are considered between solute and solvent, whereas they are fully existent in the condensed phase. In order for the Gibbs free energy to converge, the change of state from vacuum to condensed phase is subdivided into a series of intermediate states, each representing an inherent molecular simulation. These intermediates create a linking chain of shared configurational space overlap and are characterized by scaled solute-solvent interactions. As the intermediates often represent non-physical states, they are referred to as alchemical pathway and the scaling factor is the alchemical variable *λ*. In a previous study [4] we proposed our “pathfinder” method to define a set of *λ*-states with reduced computational effort but high statistical certainty. However, the quality of solubility predictions is greatly affected by the molecular models that describe the intermolecular interactions.

Several studies compared different molecular models, the so called force fields, for different sets of test systems [5–15]. Widely used molecular models such as the General Amber Force Field (GAFF) [16], the Optimized Potentials for Liquid Simulations model (OPLS-AA) [17] or the CHARMM General Force Field (CGenFF) [18] are applied for the description of drug-like molecules and are categorized as “Class 1” force fields. This class of models is characterized by the usage of fixed partial charges located on the atom’s center of mass for the evaluation of electrostatics by Coulomb’s law. Class 1 force fields represent a moderate computational effort, which makes them applicable for systems with high time constants or simulation techniques such as free energy calculations, which require increased effort due to the multitude of intermediate *λ*-states. A significant disadvantage is the lacking ability to represent polarization effects. This means that physical meaningful phenomena, like for the transition of a solute from a vacuum into a condensed phase, cannot be described adequately. Although polarizable models [19–30] could be used, these are considered computational expensive [31], while not necessarily more accurate than Class 1 force fields due to the more demanding parametrization [29]. For an at least implicit representation of polarization effects for solvation free energy calculations, Cerutti *et al*. [32] developed the IPolQ („implicitly polarized charges“) method, which was later modified by Muddana *et al*. [11] and referred to as IPolQ-Mod. In an extensive study [6], we compared solvation free energy results obtained with GAFF and its default two-stage RESP (“restrained electrostatic potential”) [33] partial charge scheme, and GAFF with IPolQ-Mod partial charges. We concluded a general compatibility of the GAFF model and the IPolQ-Mod method, though we highlighted shortcomings for specific compound classes due to the disturbed self-consistency of the molecular model. As a consequence, we recommended the refitting of the atom type specific parameters *ε* and *σ* of the Lennard-Jones (LJ) potential:


(2)

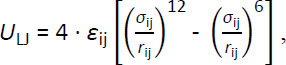



with *r*_ij_ being the distance between atoms i and j. In this study we present our methodology to optimize a large number of Lennard-Jones parameters based on GAFF for an improved representation of densities and particularly solvation free energies. We thereby consider a large number and diversity of systems and optimized atom types. Our aim is to provide model parameters to restore the self-consistency of the GAFF model in combination with IPolQ-Mod charges, as stressed in our previous work [6]. In detailed analyses we compare our newly developed model parameters with results from the standard GAFF model with both standard RESP and IPolQ-Mod partial charges for different data sets. We conclude this manuscript with a summary of our findings.

## Methodology

### Targeted functional groups, atom types and data sets

The choice of atom types for the re-parametrization was based on the analysis of substance groups occurring in potential active pharmaceutical ingredients or relevant solvents [34]. This includes alkanes, alkenes, alkynes, cycloalkanes, arenes, azoles, azines, amides, nitriles, aldehydes, ketones, alcohols, phenols, amines, ethers as well as haloalkanes, i.e. compounds with bonded fluorine, chlorine, bromine and iodine atoms. GAFF includes a variety of atom types that feature identical Lennard-Jones, but different bonded parameters. As the chemical environments for these types are comparable, no distinction was made in the re-parametrization. Furthermore, only non-hydrogen atom types were considered. Table 1 summarizes the GAFF atom types included in the refitting of the LJ-parameters.

Besides solvation free energies, we also chose liquid densities *ρ* of pure compounds in a broad temperature range as target quantities in order to allow for accurate solubility predictions as well as for precise descriptions of liquid bulk phases. The data set considered in the refitting process consists of 357 solvation free energy systems and 384 densities for small model compounds. For validation, three additional data sets were used. Set I and II cover 100 solvation free energy systems for small model compounds respectively 42 solvation free energy systems with solutes haloperidol, phenacetin, temazepam and trimethoprim. For the latter, relative solubilities were calculated according to Eq. (1) and compared to experimental relative solubility data. In validation data set III, 100 liquid densities of binary mixtures were considered. None of the validation systems were included in the refitting process. The various data sets are summarized in Table 2.

All data sets except for validation II include water as solvent compound. New parameters for solutes dissolved in water were derived and tested both for the TIP3P [35], as well as for the TIP4P/2005 [36] water models. While TIP4P/2005 is considered to be a multi-purpose model for the description of liquid water, default GAFF parameters were derived using TIP3P. As the Lennard-Jones parameters for the water models themselves were unaltered, adaptable interaction parameters *ξ*_ij_ and *ζ*_ij_ were used in the Lorentz-Berthelot combining rules, i.e.


(3)

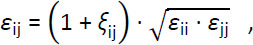



and


(4)

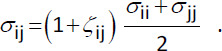



Index i refers to the atom type of interest, while index j represents the oxygen atom type of the corresponding water model. Hydrogen atom types for both water models do not participate in Lennard-Jones interactions. For all but the water interactions, parameters *ξ*_ij_ and *ζ*_ij_ were set to zero.

Our main aim in the composition of the data sets was to consider both, diverse compound pairs and a broad temperature range for the density calculations. All simulation results are given as numerical values in the Supporting Information.

### Molecular models

The adaptation of Lennard-Jones parameters is based on GAFF Version 1.8. Molecule topologies were generated using Antechamber [37] from AmberTools18 [38], followed by the transfer into the GROMACS [39–45] format using ACPYPE [46]. For comparison, we performed all simulations with default GAFF parameters using RESP [33] partial charges (GAFF/RESP), while the new Lennard-Jones parameters were specifically derived for the IPolQ-Mod method (GAFF/IPolQ-Mod+LJ-Fit). For the fitting as well as the validation sets I and III, we also considered IPolQ-Mod charges with default GAFF parameters (GAFF/IPolQ-Mod) for the sake of comparison. Partial charges were calculated using ab initio simulations in Gaussian09 [47]. Therefore, an energy optimization was performed for each system at the HF/6-31G* [48–58] level of theory prior to the partial charge calculation. For GAFF/RESP, the electrostatic potential (ESP) was calculated at HF/6-31G*, and partial charges were fitted according to the two-stage restrained electrostatic potential (RESP) [33] to match the ESP. However, this approach cannot be applied to compounds with bonded iodine, as this element is not included in the 6-31G* basis set. Although GAFF should be compatible with partial charges derived by the semi-empirically AM1-BCC [59, 60] method, Muddana *et al*. [11] highlighted that results for Δ*G*_solv_ obtained by using RESP and AM1-BCC partial charges may differ by several kJ/mol. Therefore, i.e. for the sake of consistency, no AM1-BCC charges were applied, and systems including iodine were dismissed from simulations using GAFF/RESP.

For deriving the IPolQ-Mod partial charges, the MP2/aug-cc-pVDZ [61–70] level of theory was used for all ESP calculations as initially proposed by Muddana *et al.* [11]. For the solutes in Δ*G*_solv_ simulations, two sets of ESP’s were calculated. One ESP represents the condensed phase by applying a polarizable continuum model [71] of the solvent (Gaussian keyword: SCRF=PCM), while no continuum model is applied for the representation of the vacuum phase. Partial charges from both ESP calculations were derived by RESP fitting. The charge sets were then averaged for an implicit representation of polarization effects caused by the transition of the solute from the vacuum into the solvent phase. As free energy simulations describe the behavior of the solute in infinite dilution, charges for the solvent compounds were derived in solvent phase only.

For the density calculation of a binary mixture with compounds A and B, the partial charge for an atom i in compound A *q*_i_^A^ is calculated according to Eq. (5):


(5)

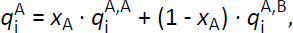



whereas *x*_A_ is the mole fraction of A in the binary mixture. Expressions *q*_i_^A,A^ and *q*_i_^A,B^ are the partial charges for atom *i* in compound *A* considering the continuum models for compounds A and B respectively. For compounds including the element iodine, a pseudopotential aug-cc-pVDZ basis set [72, 73] from the EMSL Basis Set Library [74, 75] was employed.

### Objective approach and optimization algorithms

The adaptation of Lennard-Jones parameters has been widely discussed [13, 15, 76, 77]. In order to reduce the complexity of the optimization task, pair interactions can be adapted sequentially. As the issue of high computational efforts remains, we have studied the applicability of a thermodynamic cycle approach to obtain accurate free energy results but with significant decreased expenses [78]. This approach is also employed here, when possible.

In our optimization, we target both solvation free energies and liquid densities. While the focus of our work is the accurate prediction of Δ*G*_solv_ respectively relative solubilities, the ability to describe liquid bulk phases is essential for the transfer of our new model parameters to further applications. Eq. (6) displays the chosen objective function *Z^i^* for optimization step i.


(6)

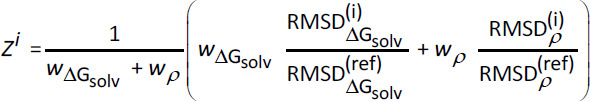



The root-mean-square deviations (RMSD) describe the divergence between simulation and experimental data. Given the two target properties Δ*G*_solv_ and *ρ*, we normalize the RMSD for Δ*G*_solv_ and *ρ* with regards to reference results (ref) obtained from simulations with unaltered model parameters. RMSD ratios are weighted by *w*_Δ_*_G_*_solv_ and *w*_p_, individually defined to obtain accurate results for both Δ*G*_solv_ and *ρ*.

Lennard-Jones parameters *ε*_ii_ and *σ*_ii_ respectively *ξ*_ij_ and *ζ*_ij_ are not explicitly associated with target properties Δ*G*_solv_ and *ρ*, as Δ*G*_solv_ depends on both enthalpic interactions as well as entropic effects between solute and solvent molecules. This requires the application of a 2-dimensional optimization algorithm. A further issue is the occurrence of statistical noise [63]. Optimization algorithms typically evaluate the change of the target function inflicted by small parameter changes. If the change in simulation results and the statistical noise are in the same order of magnitude, the optimization is likely to fail. This especially concerns derivation-based algorithms, as the usually applied difference quotients require particular small parameter changes. As a consequence, Faller *et al*. [79] proposed the usage of the derivative free and robust Downhill Simplex by Nelder and Mead [80], which was used for several subsequent force field optimizations [81–85]. To prevent local optima, optimization cycles have been repeated with differently orientated initial simpli. The optimization procedure including the thermodynamic cycle approach has been implemented into Python for a fully automated workflow.

### Simulation details

All molecular simulations were performed using GROMACS 2016.1 or 2018.1. For all simulations, the stochastic dynamics integrator [86] with a time step of δ*t* = 0.5 fs was employed, which additionally controlled the temperature using an inverse friction constant of *τ_T_* = 2.5 ps. The pressure was set to *p* = 1 atm and adjusted by the Berendsen barostat [87] during equilibration respectively the Parrinello-Rahman barostat [88] during production phases, each with a time constant of *τ_p_* = 5 ps. The particle mesh Ewald scheme with interpolation order 4, a real space cutoff radius *r*_coulomb_ = 1.2 nm and a Fourier spacing of 0.12 nm was employed for the calculation of electrostatic interactions. The cutoff radius for Lennard-Jones interactions was set to *r*_vdW_ = 1.2 nm, and long-range corrections were applied for energy and pressure calculations. System sizes exceed the recommendations proposed by Parameswaran and Mobley [89], and initial configurations were created using PACKMOL [90]. The rigid water models TIP3P and TIP4P/2005 were constrained using the settle algorithm [91]. For all solvation free energy calculations, the 1-1-48 softcore-potential with *α* = 0.003 was used [92, 93], while enthalpy differences for the calculation of Δ*G*_solv_ were written out every 100 steps. For the evaluation of Δ*G*_solv_ we used the Multistate Bennett Acceptance Ratio (MBAR) method [94] as implemented in the “alchemical_analysis.py” tool [95]. The evaluations for density calculations were performed using the GROMACS “gmx energy” utility, and their uncertainties refer to the standard error of the mean using 5 blocks of equal length.

Further simulation details presented in the following are specific to the target property, and depend on whether the simulation was performed as part of the parameter refitting or validation. For the non-refitting simulations, density calculations were performed from production runs of 4e6 simulation steps after a short energy minimization and an equilibration run of 2e6 steps. Solvation free energy calculations were performed following the workflow of our “pathfinder” method [4]. Given an initial number of *λ*-states, an energy minimization and an equilibration of 4e5 steps was performed for each state. Following that, the number and distribution of *λ*-states was adjusted in 3 to 5 trial simulations of 4e5 steps each. The aim of the trial simulations is to obtain equal partial free energy uncertainties for an overall improvement of statistical certainty [96, 97], while simultaneously ensuring sufficient configurational space overlap. This is followed by 5 productions runs of 4e6 simulations steps, which were evaluated using the block averaging technique.

For density calculations during the refitting, 4e5 equilibration steps, respectively 3.6e6 production steps were performed. To reduce the equilibration period, initial configurations were used from completed runs of similar model parameters. The number and distribution of *λ*-states was not adjusted during the fitting but adopted from corresponding GAFF/IPolQ-Mod simulations, although the existence of sufficient configurational space overlap was carefully monitored. Equivalent to the density calculations, initial configurations were taken from previous optimization steps. By this, equilibration could be reduced to 4e5 steps, while for production 4.6e6 to 5.6e6 steps were used depending on the dynamics of the systems. All Δ*G*_solv_ calculations in the refitting procedure were evaluated using bootstrapping analysis [98]. The simulation protocols were carefully tested to ensure reproducibility of the results.

### Procedure of the atom type adaptation

Due to the complexity of the optimization problem, atom types were not adjusted simultaneously, but in a subsequent manner. That means that for a current atom type optimization, only compounds were included that featured already optimized atom types. By this, the number of atom types considered, and thus the complexity of compounds increased during the optimization process. Atom types that occur in compounds with a large volume of reference data were prioritized in the order of their adaptation. For the following atom type adjustments, the number and diversity of the considered components thus quickly increased.

**Figure 1.**
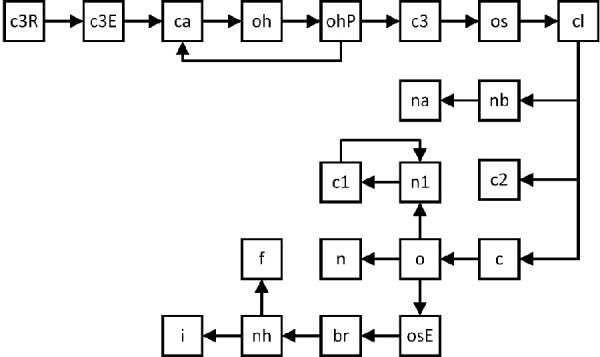
Illustration of the order of the atom type adaptation with newly defined atom types.

In the following, the procedure of the adaptation will be discussed, referring to atom types presented in Table 1. Figure 1 shows the order of the atom type adaptation.

At first, we optimized the sp^3^ hybridized carbon in ring structures (c3R), followed by the sp^3^ hybridized carbon in acyclic hydrocarbons. The aromatic sp^2^ hybridized carbon (ca) was adapted in connection with the subsequent oxygen type in alcohols and phenols. Due to the poor performance of Δ*G_solv_* predictions for both alcohols and phenols, we decided to differentiate between an oxygen type in alcohols (oh) and phenols (ohP). Because of difficulties in representing the solvent n-octanol, a sp^3^ hybridized carbon (c3) was introduced as additional atom type. That is, we differentiate between the sp^3^ hybridized carbons of a CH_2_-group within an alkyl chain (c3), the carbons in a CH_3_-group at the end of an alkyl chain (c3E), and the c3R carbon type in ring structures described in the beginning.

After the adaptation of the oxygen type in ethers (os) and the chlorine atom type (cl), atom types for nitrogen in arenes (nb) as well as sp^2^ hybridized carbon in alkenes (c2) and c=o structures (c) were optimized independently. After the refitting of type nb, the sp^2^ nitrogen in heteroaromatics (na) was modified. The adaptation of types c and o was performed successively for the description of c=o in aldehydes, ketones and esters, the latter featuring a newly defined atom type osE. After that, atom types n in amides and n1 in nitriles were targeted. For nitrile compounds, both characteristic atom types n1 and c1 were adapted successively again. Atom type bromine (br) was adjusted considering the optimized ester parameters, followed by nitrogen in amines (nh). The halogen atom types fluorine (f) and iodine (i) were re-parametrized at the end. For each of these atom types, the adjustable interaction parameters *ξ*_ij_ and *ζ*_ij_ for the description of interactions between any atom type i and the oxygen atom type in TIP3P respectively TIP4P/2005 were optimized analogously.

## Results and discussion

### Evaluation of the refitting

**Figure 2.**
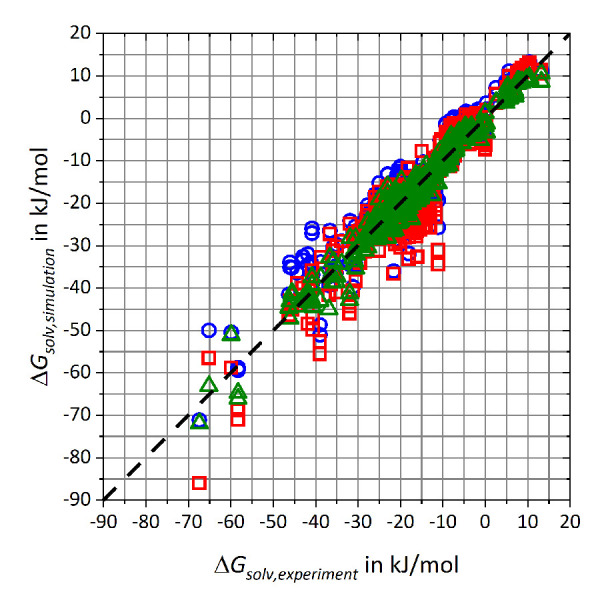
Comparison of solvation free energies ΔGsolv,simulation vs. experimental data ΔGsolv,experiment [92, 93, 99, 100] from the refitting data set. The results are represented by blue circles for GAFF/RESP, red squares for GAFF/IPolQ-Mod and green triangles for GAFF/IPolQ-Mod+LJ-Fit.

Overall, 21 atom types respectively 42 pairs of adaptable coefficients were adjusted in the refitting process. The newly derived parameters are given in the Supporting Information. In Figure 2, simulation results of the free energy of solvation Δ*G*_solv,simulation_ for the refitting data set are given for GAFF/RESP, GAFF/IPolQ-Mod and GAFF/IPolQ-Mod+LJ-Fit with respect to experimental data Δ*G*_solv,experiment_ [92, 93, 99, 100]. The diagram highlights the broad deviations of the results especially for GAFF/IPolQ-Mod and to a minor degree also for GAFF/RESP, while results for GAFF/IPolQ-Mod+LJ-Fit show significant better agreement with the experimental data.

RMSD and MAE deviations as well as the linear regression fits are summarized in Table 3. The table highlights that the RMSD value of GAFF/IPolQ-Mod+LJ-Fit has been decreased by approximately 2 kJ/mol respectively 3 kJ/mol compared to GAFF/RESP and GAFF/IPolQ-Mod. Furthermore, both the slope *m* of the linear function and the Pearson coefficient *R* are closest to 1.

**Figure 3.**
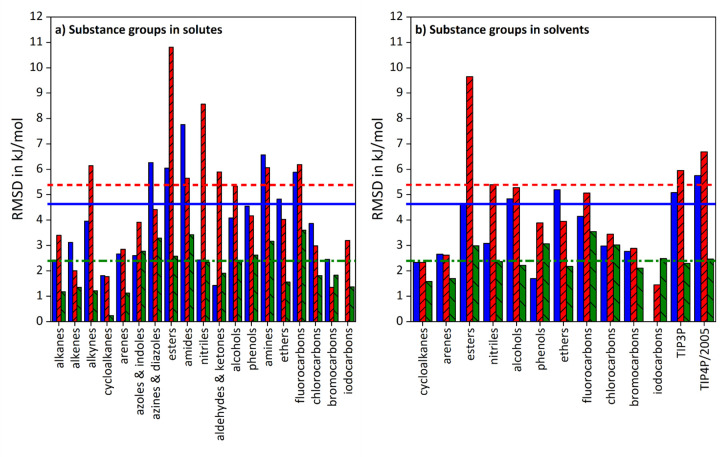
Comparison of RMSD-values for the solvation free energy results from the refitting data set obtained with the various model parameters. In diagram a), RMSD values are given aggregated by substance groups in solutes, while in diagram b), RMSD values are given by substance groups in solvents. Blue full bars refer to GAFF/RESP, red bars with rising pattern to GAFF/IPolQ-Mod and green bars with sloping pattern to GAFF/IPolQ-Mod+LJ-Fit. The bold horizontal lines indicate the overall RMSD values for the data set, whereas the continuous lines represents GAFF/RESP, the dashed lines GAFF/IPolQ-Mod and the dash-dotted lines GAFF/IPolQ-Mod+LJ-Fit.

In Figure 3, RMSD-values are given for solvation free energy results clustered by substance groups of solutes respectively solvents.

The arrangement by solutes in diagram a) demonstrates that GAFF/IPolQ-Mod+LJ-Fit yields better results than GAFF/RESP for all groups except for azoles and indoles, as well as aldehydes and ketones. However, the performance for azoles and indoles is almost equal, while the RMSD value for aldehydes and ketones lies well below its total RMSD value, indicated by the horizontal dash-dotted line. The comparison with GAFF/IPolQ-Mod further shows better performance for all groups but bromine compounds, while the corresponding RMSD is again smaller than the overall RMSD. Regarding the deviations by solvent in diagram b), GAFF/IPolQ-Mod+LJ-Fit describes phenols rather poorly compared to GAFF/RESP and GAFF/IPolQ-Mod, while this is distinctively reversed for the grouping by solutes. This is analogous to iodine compounds and GAFF/IPolQ-Mod. For all the other groups, GAFF/IPolQ-Mod+LJ-Fit yields smaller RMSD deviations.

That is, the performance of GAFF/IPolQ-Mod+LJ-Fit is much more homogenous for both solute and solvent groups compared to GAFF/RESP and GAFF/IPolQ-Mod. While these exhibit individual weaknesses, for example in the description of azines & diazoles, esters, amides and nitriles, the level of accuracy for GAFF/IPolQ-Mod+LJ-Fit remains nearly constant for the Δ*G*_solv_ refitting data set.

Some compounds of the refitting data set were also subject of a previous work [101], in which we have compared the performance of different force fields regarding the preproduction of hydration free energies. In the SI we provide the new results of GAFF/IPolQ-Mod+LJ-Fit for this small set of test systems considered in [101] compared to the previous results from GAFF/RESP and GAFF/IPolQ-Mod as well as CGenFF and OPLS-AA.

**Figure 4.**
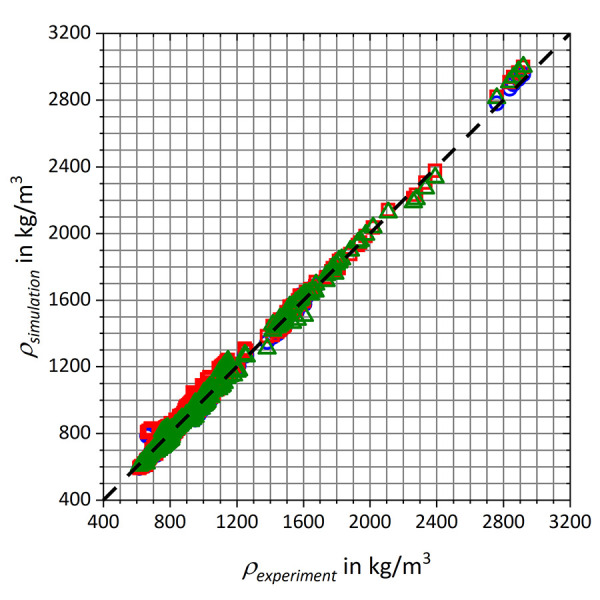
Comparison of simulated densities ρsimulation vs. experimental reference data ρexperiment [102–131] from the refitting data set. The results are represented by blue circles for GAFF/RESP, red squares for GAFF/IPolQ-Mod and green triangles for GAFF/IPolQ-Mod+LJ-Fit.

Simulation results of the density *ρ*_simulation_ from the refitting data sets are given in Figure 4 *vs*. experimental data *ρ*_experiment_ [102–131].

Table 4 summarizes the root-mean-square deviations, mean absolute errors (MAE) as well as the slopes *m* and the Pearson correlation coefficients *R* of the linear regression curves. GAFF/IPolQ-Mod+LJ-Fit demonstrates slightly improved agreement with experimental data compared to GAFF/RESP, while the results for GAFF/IPolQ-Mod are significantly worse.

**Figure 5.**
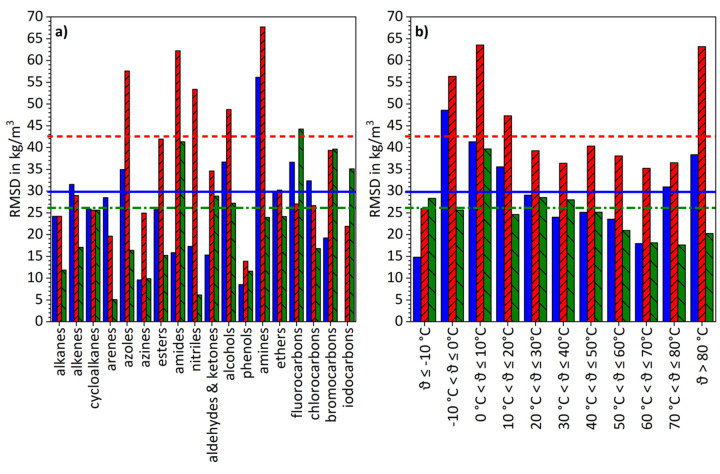
Comparison of RMSD-values for the density results from the refitting data set obtained with the various model parameters. In diagram a), RMSD values are given aggregated by substance groups, while in diagram b), RMSD values are clustered by temperature intervals. Blue full bars refer to GAFF/RESP, red bars with rising pattern to GAFF/IPolQ-Mod and green bars with sloping pattern to GAFF/IPolQ-Mod+LJ-Fit. The bold horizontal lines indicate the overall RMSD values for the data set, whereas the continuous lines represents GAFF/RESP, the dashed lines GAFF/IPolQ-Mod and the dash-dotted lines GAFF/IPolQ-Mod+LJ-Fit.

In Figure 5, RMSD values for the three sets of model parameters are presented by groups of substances respectively temperature intervals. As for GAFF/IPolQ-Mod, large deviations occur for amines, amides, azoles, nitriles and alcohols, respectively, for temperature ranges -10 °C < *ϑ* ≤ 20 °C as well as for *ϑ* > 80 °C, while GAFF/RESP demonstrates poor performance for amines and temperatures within the range of  -10 °C < *ϑ* ≤ 0 °C. GAFF/IPolQ-Mod+LJ-Fit shows lacking accuracies for amides, as well as for fluorocarbons, bromocarbons and iodocarbons. However, the impact of the temperature is less pronounced.

### Evaluation of the Validation I Data Set

**Figure 6.**
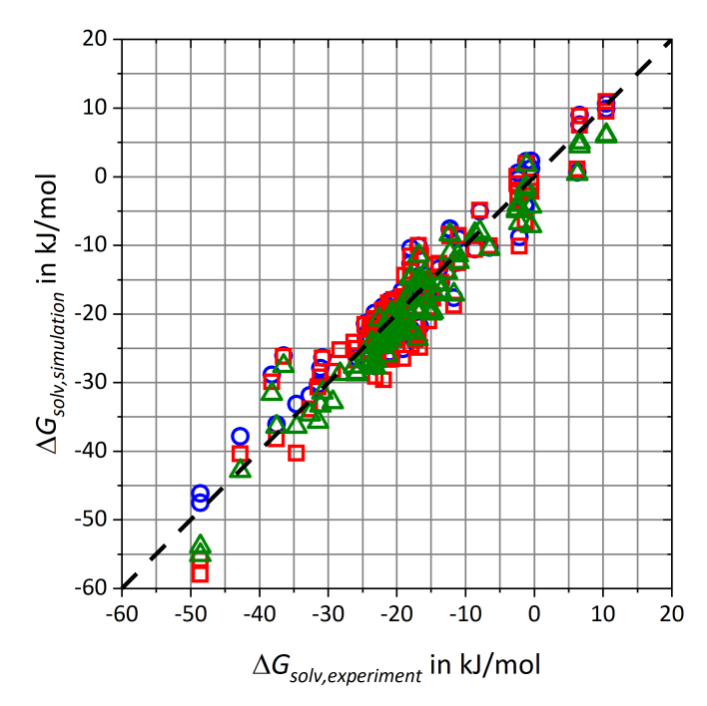
Comparison of simulated solvation free energies ΔGsolv,simulation vs. experimental reference data ΔGsolv,experiment [99] from the validation I data set. The results are represented by blue circles for GAFF/RESP, red squares for GAFF/IPolQ-Mod and green triangles for GAFF/IPolQ-Mod+LJ-Fit.

Figure 6 shows the simulation results of the solvation free energies from the validation I data set over experimental data [99].

The RMSD and MAE deviations as well as the slopes of the regression curves and the Pearson correlation coefficients are summarized in Table 5. The results shown in Figure 6 and Table 5 illustrate that although GAFF/IPolQ-Mod+LJ-Fit allows for the most accurate predictions of the solvation free energy, the RMSD and MAE deviations for the validation I data set are much higher than for the refitting data set shown in Table 3. In contrast to this, the deviations for GAFF/RESP and GAFF/IPolQ-Mod have decreased significantly, so that GAFF/RESP and GAFF/IPolQ-Mod+LJ-Fit show very similar accuracies in the prediction of Δ*G*_solv_.

**Figure 7.**
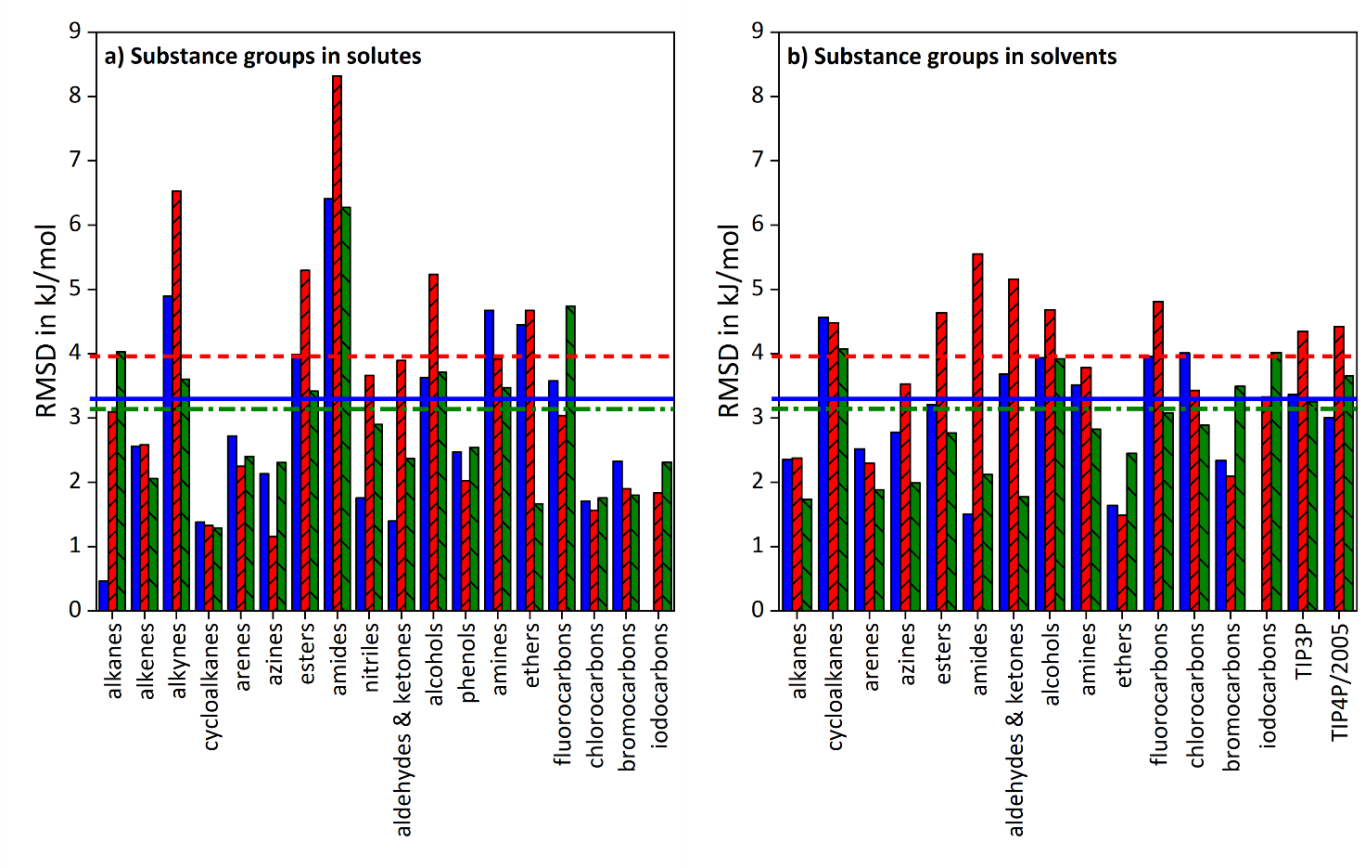
Comparison of RMSD-values for the solvation free energy results from the validation I data set obtained with the various model parameters. In diagram a), RMSD values are given aggregated by substance groups in solutes, while in diagram b), RMSD values are given by substance groups in solvents. Blue full bars refer to GAFF/RESP, red bars with rising pattern to GAFF/IPolQ-Mod and green bars with sloping pattern to GAFF/IPolQ-Mod+LJ-Fit. The bold horizontal lines indicate the overall RMSD values for the data set, whereas the continuous lines represent GAFF/RESP, the dashed lines GAFF/IPolQ-Mod and the dash-dotted lines GAFF/IPolQ-Mod+LJ-Fit.

Figure 7 demonstrates the RMSD deviations for substance groups in solute and solvent molecules.

Remarkable is the poor description of alkanenes, amides and fluorocarbons as solutes with the new parameters. However, the discussed groups of substances as solvents exhibit better-than-average RMSD values. This suggests that the corresponding substance groups or the atom types associated with them do not generally fail to reflect interactions. This raises the question whether identical atom types are justified for solute and solvent if the partial charges of the molecules are determined differently and the polarization effects are only approximated with IPolQ-Mod. Based on this consideration, there is a risk of underfitting model parameters if no distinction is made between the atom type adaptation in the solute or solvent. Consequently, individual pair potentials might be ideal, though this clearly leads the concept of a general force field ad absurdum.

However, not only do the RMSD values differ significantly between refitting- and validation I data set for GAFF/IPolQ-Mod+LJ-Fit, but also between GAFF/RESP and GAFF/IPolQ-Mod. The differences in the RMSD values between refitting and validation I data sets are ∆RMSD_GAFF/RESP_ = 1.34 kJ/mol,  ∆RMSD_GAFF/IPolQ-Mod_ = 1.43 kJ/mol and ∆RMSD_GAFF⁄IPolQ-Mod+LJ-Fit_ = -0.75 kJ/mol. As the systems from both data sets are of similar complexity and as identical simulation protocols were used leading to comparable statistical accuracies, these discrepancies cannot be attributed to systematic errors in the calculation of Δ*G*_solv_. A further analysis (see SI) highlights that the accuracies obtained from the validation I data set do not represent the predictive quality of neither of the models.

### Evaluation of the validation II data set

**Figure 8.**
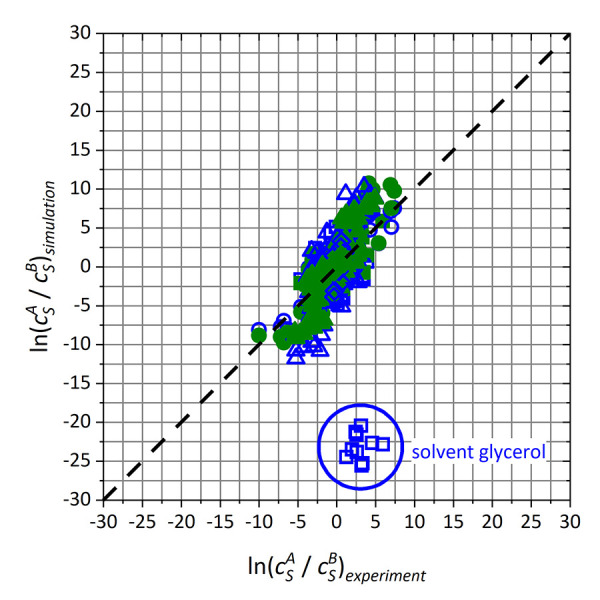
Relative solubilities obtained from simulated solvation free energies of the validation II data set over experimental data [132]. Blue hollow symbols refer to GAFF/RESP, while green full symbols represent GAFF/IPolQ-Mod+LJ-Fit. Solutes haloperidol, phenacetin, temazepam and trimethoprim are displayed by squares, circles, triangles and diamond shapes respectively.

As a consequence, the validation II data set was introduced for the prediction of relative solubilities for the more complex solute substances haloperidol, phenacetin, temazepam and trimethoprim. Based on the calculation of 42 solute/solvent pairs, 237 individual relative solubilities can be determined according to Eq. (1) using combinatorics, which are plotted against experimental data [132] in Figure 8.

Due to the consistently poor performance of GAFF/IPolQ-Mod, only GAFF/RESP and GAFF/IPolQ-Mod+LJ-Fit models are compared for the validation II data set. Figure 8 illustrates the poor description of relative solubilities for solvate haloperidol and solvent glycerol with GAFF/RESP. However, other relative solubilities also show significant deviations from the experimental reference data, especially for GAFF/RESP. Table 6 summarizes RMSD and MAE deviations as well as slopes *m* and coefficients *R* of the regression lines. Even when the haloperidol/glycerol systems are excluded, GAFF/IPolQ-Mod+LJ-Fit has a significantly better overall accuracy than GAFF/RESP.

Depending on whether the haloperidol/glycerol outliers are taken into account, the reduction of the RMSD value from the validation II data set for GAFF/IPolQ-Mod+LJ-Fit compared to GAFF/RESP is between 24 - 63%, which corresponds to a mean value of approximately 44%. This value agrees very well with the decrease in the RMSD value of about 42% for a hypothetical basis population from the refitting and validation I data set as discussed in the SI. In contrast, the validation I data set shows a decrease of only 5%. We therefore conclude that the validation II data set gives a more reasonable estimate for the potential of our newly developed model parameters aiming at improved predictions of solvation free energies and relative solubilities.

### Evaluation of the validation III data set

**Figure 9.**
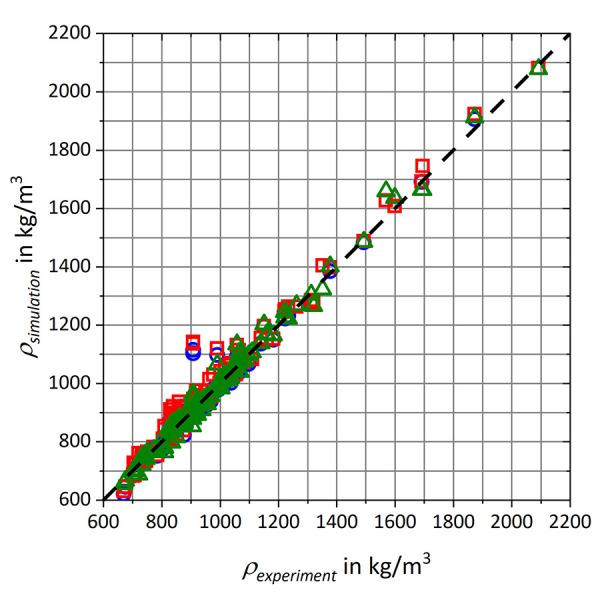
Comparison of simulated densities ρsimulation vs. experimental reference data ρexperiment [102, 118, 120, 133–138] for binary mixtures from the validation III data set. The results are represented by blue circles for GAFF/RESP, red squares for GAFF/IPolQ-Mod and green triangles for GAFF/IPolQ-Mod+LJ-Fit.

For validation III, 100 liquid densities of binary mixtures were calculated. Simulation results over experimental reference data [102, 118, 120, 133–138] are given in Figure 9.

The evaluation of the predictive performance by set of model parameters is summarized in Table 7.

**Figure 10.**
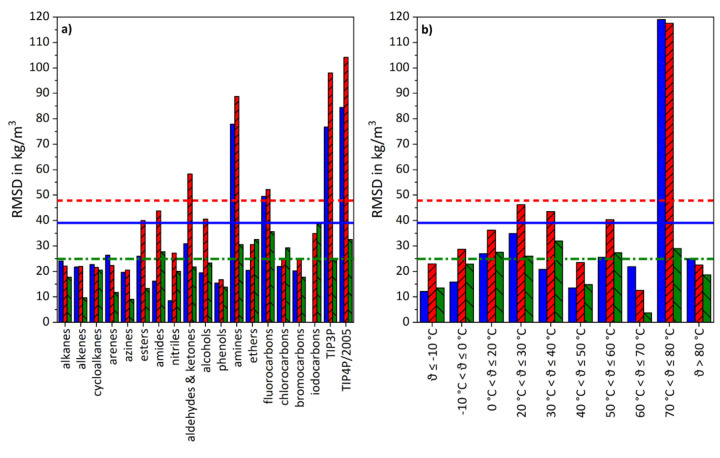
Comparison of RMSD-values for the density results from the validation III data set obtained with the various model parameters. In diagram a), RMSD values are given aggregated by substance groups, while in diagram b), RMSD values are clustered by temperature intervals. Blue full bars refer to GAFF/RESP, red bars with rising pattern to GAFF/IPolQ-Mod and green bars with sloping pattern to GAFF/IPolQ-Mod+LJ-Fit. The bold horizontal lines indicate the overall RMSD values for the data set, whereas the continuous lines represent GAFF/RESP, the dashed lines GAFF/IPolQ-Mod and the dash-dotted lines GAFF/IPolQ-Mod+LJ-Fit.

The comparison of results in Table 7 demonstrates high correlations *R* and regression slopes *m* close to 1 for all parameter sets. However, the RMSD value for GAFF/IPolQ-Mod+LJ-Fit decreased by approximately  41% and 48% compared to GAFF/RESP and GAFF/IPolQ-Mod, respectively, and is in close agreement to the accuracies presented in Table 4. RMSD values aggregated by substance groups respectively temperature intervals are displayed in Figure 10.

For GAFF/RESP and GAFF/IPolQ-Mod, the left figure demonstrates shortcomings for amines as well as for both the TIP3P and TIP4P/2005 water models. Although GAFF/IPolQ-Mod+LJ-Fit indicates deviations for fluorine and iodine compounds, these outliers are still significantly smaller than those of the other parameter sets. Regarding the reproduction of experimental data over a broad temperature range, GAFF/RESP and GAFF/IPolQ-Mod show extreme faults for systems within the temperature range of 70 °C < ϑ ≤ 80 °C, while the temperature impact on GAFF/IPolQ-Mod+LJ-Fit results is comparably small.

## Conclusions

Molecular simulations offer great potential for a better understanding of complex processes such as solubility as they sample systems on the molecular level. However, accurate simulations require accurate molecular models. As polarization is considered to be an impacting factor, though linked to high computational effort, there is need for an implicit representation of polarization effects, for example using the IPolQ-Mod method. In this work, we have optimized GAFF atom types (GAFF/IPolQ-Mod+LJ-Fit) for a variety of substance groups considering IPolQ-Mod partial charges to improve the description of solvation free energies, respectively relative solubilities, as well as liquid densities. The evaluation of our refitting data set highlights significant improvements in the description of solvation free energies for our new parameters compared to default GAFF (GAFF/RESP), but especially for GAFF with IPolQ-Mod charges but not-optimized parameters (GAFF/IPolQ-Mod). The improvement regarding the prediction of liquid densities for pure compounds is minor compared to default GAFF.

Regarding the validation, the description of densities for binary mixtures is significantly better with our new parameters. However, the accuracies of the free energy predictions for default RESP and our optimized parameters are almost identical. By in-depth analyses, partly given in the SI, we conclude that the free energy validation data does not represent the overall performance for neither GAFF/RESP nor GAFF/IPolQ-Mod. We therefore deduce that the quality of our new model parameters is misrepresented as well. As a consequence, we further compared GAFF/RESP and GAFF/IPolQ-Mod+LJ-Fit for the description of relative solubilities of four drug-like structures in a multitude of solvents, resulting in a total of 237 individual relative solubilities. The improvement in root-mean square deviations between GAFF/IPolQ-Mod+LJ and GAFF/RESP of around 44% is in much better agreement with the reduction of RMSD for the total of both previous free energy data sets. By this, our newly derived parameters for GAFF in combination with IPolQ-Mod apparently allow for a significant improvement in the prediction of relative solubilities.



## Figures and Tables

**Table 1.** Standard GAFF atom types targeted at the parameter optimization.

Atom type	Description (*16*)
br	any bromine
c	sp^2^ carbon in C=O
c1 (cg)	sp^1^ carbon (in conjugated ring systems)
c2	sp^2^ carbon, aliphatic
c3	sp^3^ carbon
ca (cc / cd / ce)	sp^2^ carbon, aromatic / conjugated
cl	any chlorine
f	any fluorine
i	any iodine
n	sp^2^ nitrogen in amides
n1	sp^1^ nitrogen
na	sp^2^ nitrogen with 3 subst.
nb (n2)	aromatic nitrogen / sp^2^ nitrogen with 2 subst.
nh (n3)	amine nitrogen / sp^3^ nitrogen with 3 subst.
o	sp^2^ oxygen in C=O
oh	sp^3^ oxygen in hydroxyl groups
os	sp^3^ oxygen in ethers and esters

**Table 2.** Data sets used for fitting respectively validating new Lennard-Jones parameters.

Data set	Content
Refitting	357 Δ*G*_solv_-systems: 112 solute / 37 solvent compounds 384 *ρ*-systems: 78 compounds, Δ*T* = (183.15 … 478.15) K
Validation I	100 Δ*G*_solv_-systems: 59 solute / 34 solvent compounds
Validation II	42 Δ*G*_solv_-systems: 4 solute / 23 solvent compounds
Validation III	100 *ρ*-systems of binary mixtures: 72 compounds, Δ*T* = (183.15 … 383.15) K

**Table 3.** Summary of the evaluation of free energy results from the refitting data set. Besides the root-mean-square deviations (RMSD) and the mean absolute errors (MAE), the slopes m of the linear fitting curves with corresponding Pearson correlation coefficients R are given. For a better comparison with GAFF/RESP, additional values for GAFF/IPolQ-Mod and GAFF/IPolQ-Mod+LJ-Fit are stated in brackets that exclude iodine compounds.

	GAFF/RESP	GAFF/IPolQ-Mod	GAFF/IPolQ-Mod+LJ-Fit
RMSD in kJ/mol	4.64	5.39 (5.49)	2.39 (2.43)
MAE in kJ/mol	3.52	3.93 (4.00)	1.77 (1.79)
Slope *m*	0.9511	1.0452 (1.0397)	1.0083 (1.0085)
Pearson *R*	0.9438	0.9350 (0.9340)	0.9850 (0.9848)

**Table 4.** Summary of the evaluation of density results from the refitting data set. Besides the root-mean-square deviations (RMSD) and the mean absolute errors (MAE), the slopes m of the linear fitting curves with corresponding Pearson correlation coefficients R are given. For a better comparison with GAFF/RESP, additional values for GAFF/IPolQ-Mod and GAFF/IPolQ-Mod+LJ-Fit are stated in brackets that exclude iodine compounds.

	GAFF/RESP	GAFF/IPolQ-Mod	GAFF/IPolQ-Mod+LJ-Fit
RMSD in kg/m^3^	29.80	42.55 (43.27)	26.15 (25.66)
MAE in kg/m^3^	22.51	33.78 (34.56)	19.20 (18.80)
Slope *m*	0.9970	0.9938 (1.0036)	1.0168 (1.0266)
Pearson *R*	0.9959	0.9956 (0.9941)	0.9979 (0.9974)

**Table 5.** Summary of the evaluation of solvation free energy results from the validation I data set. Besides the root-mean-square deviations (RMSD) and the mean absolute errors (MAE), the slopes *m* of the linear fitting curves with corresponding Pearson correlation coefficients *R* are given. For a better comparison with GAFF/RESP, additional values for GAFF/IPolQ-Mod and GAFF/IPolQ-Mod+LJ-Fit are stated in brackets that exclude iodine compounds.

	GAFF/RESP	GAFF/IPolQ-Mod	GAFF/IPolQ-Mod+LJ-Fit
RMSD in kJ/mol	3.30	3.96 (4.07)	3.14 (3.09)
MAE in kJ/mol	2.56	3.14 (3.22)	2.55 (2.50)
Slope *m*	0.9192	0.9801 (0.9805)	0.9623 (0.9623)
Pearson *R*	0.9578	0.9418 (0.9412)	0.9701 (0.9706)

**Table 6.** Summary of the evaluation of solvation free energy results from the validation II data set. Besides the root-mean-square deviations (RMSD) and the mean absolute errors (MAE), the slopes m of the linear fitting curves with corresponding Pearson correlation coefficients R are given. In order to determine the effect of the outliers for GAFF/RESP, the results with haloperidol as solute and glycerol as solvent were not considered for the values in brackets. RMSD and MAE deviations for the individual solute compounds are given below the overall values.

	GAFF/RESP	GAFF/IPolQ-Mod+LJ-Fit
RMSD	6.14 (2.98)	2.28 (2.26)
haloperidol	11.53 (2.91)	2.03 (1.89)
phenacetin	1.92	2.97
temazepam	3.27	2.24
trimethoprim	2.09	0.51

MAE	3.41 (2.40)	1.74 (1.72)
haloperidol	6.78 (2.45)	1.67 (1.55)
phenacetin	1.55	2.49
temazepam	2.66	1.66
trimethoprim	1.80	0.45

Slope *m*	0.5550 (1.1032)	1.2512 (1.2311)
Pearson *R*	0.2358 (0.6859)	0.8286 (0.8183)

**Table 7.** Summary of the evaluation of density results for binary mixtures from the validation III data set. Besides the root-mean-square deviations (RMSD) and the mean absolute errors (MAE), the slopes m of the linear fitting curves with corresponding Pearson correlation coefficients R are given. For a better comparison with GAFF/RESP, additional values for GAFF/IPolQ-Mod and GAFF/IPolQ-Mod+LJ-Fit are stated in brackets that exclude iodine compounds.

	GAFF/RESP	GAFF/IPolQ-Mod	GAFF/IPolQ-Mod+LJ-Fit
RMSD in kg/m^3^	39.06	47.85 (49.08)	24.93 (22.89)
MAE in kg/m^3^	23.47	31.71 (32.04)	17.84 (16.60)
Slope *m*	1.002	0.9977 (1.0041)	1.0132 (1.0193)
Pearson *R*	0.9816	0.9856 (0.9766)	0.9954 (0.9939)
