# Supplementary material for: GAFF/IPolQ-Mod+LJ-Fit: Optimized force field parameters for solvation free energy predictions
Source: ADMET DMPK. 2020 Jun 28;8(3):274–96. doi: 10.5599/admet.837 (PMC8915609; doi:10.5599/admet.837)
Supplement: Supplementary file 1 [file admet-8-837-s001.pdf]

Supporting Information for

GAFF/IPolQ-Mod+LJ-Fit: Optimized Force Field Parameters for Solvation Free Energy Predictions

Andreas Mecklenfeld[1,2] and Gabriele Raabe[1,2]*

[1]Institut für Thermodynamik, Technische Universität Braunschweig,
Hans-Sommer Strasse 5, 38106 Braunschweig, Germany
[2] Center of Pharmaceutical Engineering, Technische Universität Braunschweig,
Franz-Liszt-Strasse 35a, 38106 Braunschweig, Germany

*Corresponding Author: E-mail: g.raabe@tu-bs.de; Tel.: +49 (531) 391 2628; Fax: +49 (531) 391 7814

1. Newly Developed Model Parameters: GAFF/IPolQ-Mod+LJ-Fit 1
2. Simulation Results 2
3. Representation of Force Field Accuracies for the Validation I Data Set 20
4. References 21

1. Newly Developed Model Parameters: GAFF/IPolQ-Mod+LJ-Fit

Table 1. Optimized GAFF parameters (GAFF/IPolQ-Mod+LJ-Fit). In the first two columns, the atom types with corresponding description is given. Columns 3 and 4 mark the Lennard-Jones parameters σ_{ii} and ε_{ii}. The last four columns represent the interaction parameters ζ_{ij} and ξ_{ij} given in Eq. (4) and Eq. (3) of our main paper for the interactions between atom type *i* and the oxygen atoms OW and OWT4 for water models TIP3P and TIP4P/2005 respectively.

Atom type	Description	σ_{ii} in nm	ε_{ii} in kJ/mol	$\zeta_{i,OW}$	$\xi_{i,OW}$	$\zeta_{i,OWT4}$	$\xi_{i,OWT4}$
br	any bromine	0.356721	1.596301	0.1261	0.0581	0.1058	0.0848
c	sp^2 carbon in C=O	0.275839	0.334737	0.1141	-0.2467	0.0268	0.4639
c1 (cg)	sp^1 carbon (in conjugated ring systems)	0.306201	0.813913	0.1745	-0.1565	0.1725	-0.1189
c2	sp^2 carbon, aliphatic	0.357044	0.379395	0.0253	0.0008	0.0227	0.0283
c3	sp^3 carbon in alkyl chain	0.346384	0.485194	-0.0097	0.0202	-0.0097	0.0199
c3E	sp^3 carbon at the end of an alkyl chain	0.360893	0.488399	0.0000	0.0000	0.0000	0.0312
c3R	sp^3 carbon in ring structures	0.339967	0.457730	0.0000	0.0504	0.0000	0.0500
ca (cc / cd / ce)	sp^2 carbon, aromatic / conjugated	0.348013	0.385153	0.0000	-0.1025	0.0159	-0.0802
cl	any chlorine	0.342743	1.039924	0.1201	0.1555	0.0662	0.1634
f	any fluorine	0.322286	0.252515	-0.0333	-0.1380	-0.0748	-0.1543
i	any iodine	0.383086	2.092000	0.0435	0.1153	0.0891	0.1023
n	sp^2 nitrogen in amides	0.304895	0.753957	0.0720	1.4476	0.0288	1.3311
n1	sp^1 nitrogen	0.347100	0.604588	0.1896	0.5836	0.1683	0.6384
na	sp^2 nitrogen with 3 subst.	0.360871	0.569024	-0.0531	0.1739	-0.0530	0.1739
nb (n2)	aromatic nitrogen / sp^2 nitrogen with 2 subst.	0.337649	0.465545	-0.0428	0.4359	-0.0194	0.3597
nh (n3)	amine nitrogen / sp^3 nitrogen with 3 subst.	0.359321	0.810859	-0.0034	0.8954	-0.0068	0.5959
o	sp^2 oxygen in C=O	0.327493	0.969942	0.0191	0.0422	0.0429	0.0184
oh	sp^3 oxygen in hydroxyl groups of alcohols	0.328770	0.913671	0.0000	0.6500	0.0000	0.4500
ohP	sp^3 oxygen in hydroxyl groups of phenols	0.306647	0.880314	0.0000	1.1000	0.0000	0.9000
os	sp^3 oxygen in ethers	0.270445	0.289945	-0.0095	0.5336	0.0271	0.0542
osE	sp^3 oxygen in esters	0.255001	0.497896	0.1573	-0.1461	0.2787	-0.3662

2. Simulation Results

2.1. Refitting Data Set: Solvation Free Energies

Table 2: Simulation results for solvation free energies ΔG_{solv} in kJ/mol from the refitting data set. The first two columns mark the solute and solvent compounds, followed by the temperature T in K and the source for the experimental reference data. This is ensued by simulation results for the model parameter sets, whereas statistical uncertainties are given in brackets.

Solute	Solvent	T	Source	GAFF/RESP		GAFF/IPolQ-Mod		GAFF/IPolQ-Mod+LJ-Fit	
				ΔG_{solv}	$\delta\Delta G_{solv}$	ΔG_{solv}	$\delta\Delta G_{solv}$	ΔG_{solv}	$\delta\Delta G_{solv}$
1,1,1-Trichlorethan	cyclohexane	298.00	[1]	-17.57	(0.10)	-17.61	(0.10)	-19.08	(0.10)
1,1,2-trichloroethane	TIP3P	298.00	[1]	-1.49	(0.09)	-2.95	(0.14)	-8.14	(0.14)
1,1,2-trichloroethane	TIP4P/2005	298.00	[1]	-0.50	(0.13)	-1.76	(0.14)	-7.59	(0.14)
1,4-dioxane	acetonitrile	298.00	[1]	-28.02	(0.12)	-27.44	(0.12)	-22.39	(0.12)
1,4-dioxane	benzonitrile	298.00	[1]	-26.73	(0.06)	-27.03	(0.44)	-20.94	(0.44)
1,4-dioxane	fluorobenzene	298.00	[1]	-24.79	(0.12)	-25.60	(0.10)	-20.79	(0.10)
1-benzylimidazole	TIP3P	298.15	[2,3]	-33.96	(0.17)	-44.32	(0.11)	-42.81	(0.11)
1-benzylimidazole	TIP4P/2005	298.15	[2,3]	-34.38	(0.22)	-46.01	(0.14)	-41.24	(0.14)
1-bromo-4-methylbenzene	TIP3P	298.00	[1]	-2.79	(0.08)	-5.26	(0.25)	-4.07	(0.25)
1-bromo-4-methylbenzene	TIP4P/2005	298.00	[1]	-0.92	(0.12)	-3.91	(0.10)	-4.77	(0.10)
1-bromobutane	TIP3P	298.00	[1]	0.60	(0.05)	-2.04	(0.10)	-2.61	(0.10)
1-bromobutane	TIP4P/2005	298.00	[1]	2.05	(0.21)	-0.83	(0.12)	-3.04	(0.12)
1-chloro-2-methylbenzene	TIP3P	298.00	[1]	-0.75	(0.06)	-2.69	(0.13)	-2.41	(0.13)
1-chloro-2-methylbenzene	TIP4P/2005	298.00	[1]	1.28	(0.12)	-0.84	(0.13)	-2.92	(0.12)
1-chloropropane	TIP3P	298.00	[1]	0.70	(0.08)	-1.32	(0.08)	-2.44	(0.08)
1-chloropropane	TIP4P/2005	298.00	[1]	1.86	(0.16)	0.04	(0.10)	-5.12	(0.10)
1H-imidazole	TIP3P	298.00	[1]	-33.97	(0.04)	-41.67	(0.10)	-43.47	(0.10)
1H-imidazole	TIP4P/2005	298.00	[1]	-34.26	(0.10)	-43.06	(0.15)	-42.28	(0.15)
1H-pyrrole	trichloromethane	298.00	[1]	-23.48	(0.13)	-25.89	(0.06)	-25.46	(0.06)
1H-pyrrole	cyclohexane	298.00	[1]	-13.71	(0.02)	-13.66	(0.13)	-15.94	(0.13)
1H-pyrrole	octan-1-ol	298.00	[1]	-20.90	(0.43)	-22.47	(0.36)	-20.79	(0.36)
1H-pyrrole	TIP3P	298.15	[2,3]	-21.75	(0.04)	-24.25	(0.13)	-21.88	(0.13)
1H-pyrrole	TIP4P/2005	298.15	[2,3]	-22.57	(0.14)	-25.36	(0.08)	-22.22	(0.08)
1-methoxypropane	TIP3P	298.00	[1]	-1.97	(0.08)	-2.80	(0.11)	-6.64	(0.11)
1-methoxypropane	TIP4P/2005	298.00	[1]	-2.34	(0.08)	-3.02	(0.11)	-7.48	(0.11)
1-methylpyrrole	TIP3P	298.15	[2,3]	-13.13	(0.04)	-16.86	(0.10)	-13.76	(0.10)
1-methylpyrrole	TIP4P/2005	298.15	[2,3]	-14.05	(0.07)	-18.22	(0.07)	-13.80	(0.07)
1-phenylethan-1-one	TIP3P	298.00	[1]	-18.49	(0.18)	-26.20	(0.10)	-17.24	(0.10)
1-phenylethan-1-one	TIP4P/2005	298.00	[1]	-18.12	(0.21)	-26.84	(0.21)	-17.73	(0.21)
2,2,2-trifluoroethan-1-ol	trichloromethane	298.00	[1]	-15.93	(0.10)	-16.55	(0.05)	-18.12	(0.05)
2,2,2-trifluoroethan-1-ol	TIP3P	298.00	[1]	-20.48	(0.09)	-22.02	(0.07)	-19.07	(0.07)
2,2,2-trifluoroethanol	TIP4P/2005	298.00	[1]	-18.64	(0.06)	-20.09	(0.09)	-19.86	(0.09)
2,4-dimethylpyridine	TIP3P	298.00	[1]	-11.81	(0.09)	-18.08	(0.12)	-21.05	(0.11)
2,4-dimethylpyridine	TIP4P/2005	298.00	[1]	-12.60	(0.09)	-19.63	(0.24)	-21.35	(0.24)
2-butan-2-one	fluorobenzene	298.00	[1]	-18.56	(0.09)	-21.43	(0.12)	-20.50	(0.12)
2-chlorobutane	TIP3P	298.00	[1]	2.09	(0.13)	0.28	(0.05)	-1.24	(0.05)
2-chlorobutane	TIP4P/2005	298.00	[1]	3.55	(0.16)	1.92	(0.08)	-3.29	(0.08)
2-fluorophenol	TIP3P	298.15	[2,3]	-17.43	(0.26)	-17.87	(0.13)	-19.28	(0.13)
2-fluorophenol	TIP4P/2005	298.15	[2,3]	-15.08	(0.07)	-15.63	(0.17)	-20.42	(0.17)
2-iodopropane	TIP3P	298.00	[1]			-0.04	(0.09)	-3.76	(0.09)
2-iodopropane	TIP4P/2005	298.00	[1]			1.13	(0.16)	-3.90	(0.16)
2-methoxy-2-methylpropane	TIP3P	298.00	[1]	-1.30	(0.10)	-3.22	(0.12)	-7.79	(0.12)
2-methoxy-2-methylpropane	TIP4P/2005	298.00	[1]	-1.55	(0.20)	-3.77	(0.13)	-8.83	(0.13)
2-methoxypropane	TIP3P	298.00	[1]	-2.06	(0.13)	-3.49	(0.07)	-7.51	(0.07)
2-methoxypropane	TIP4P/2005	298.00	[1]	-2.31	(0.24)	-4.06	(0.16)	-8.76	(0.16)
2-methylphenol	pentan-1-ol	298.00	[1]	-35.73	(0.28)	-36.23	(0.34)	-38.70	(0.34)
2-methylphenol	cyclohexane	298.00	[1]	-21.46	(0.09)	-21.38	(0.14)	-25.89	(0.14)
2-methylphenol	ethylbenzene	298.00	[1]	-26.59	(0.12)	-27.69	(0.08)	-32.13	(0.08)
2-methylphenol	toluene	298.00	[1]	-27.18	(0.07)	-28.22	(0.08)	-32.94	(0.08)
2-methylphenol	TIP3P	298.00	[1]	-21.47	(0.12)	-24.14	(0.15)	-25.55	(0.15)
2-methylphenol	TIP4P/2005	298.00	[1]	-20.34	(0.34)	-22.73	(0.24)	-25.25	(0.24)
2-methylprop-1-ene	TIP3P	298.00	[1]	7.49	(0.07)	5.23	(0.06)	4.26	(0.06)
2-methylprop-1-ene	TIP4P/2005	298.00	[1]	8.63	(0.11)	5.90	(0.09)	3.62	(0.09)
2-methylpropan-2-ol	octan-1-ol	298.00	[1]	-26.58	(0.21)	-30.49	(0.40)	-24.18	(0.40)
2-methylpropan-2-ol	TIP3P	298.00	[1]	-20.04	(0.14)	-21.72	(0.06)	-18.95	(0.06)
2-methylpropan-2-ol	TIP4P/2005	298.00	[1]	-21.67	(0.22)	-24.05	(0.12)	-20.45	(0.12)
3-methyl-1H-indole	cyclohexane	293.00	[4]	-28.97	(0.11)	-28.93	(0.05)	-34.27	(0.05)
3-methyl-1H-indole	trichloromethane	293.00	[4]	-39.33	(0.21)	-41.67	(0.11)	-44.95	(0.11)
3-methyl-1H-indole	TIP3P	293.00	[4]	-22.05	(0.10)	-26.34	(0.11)	-24.58	(0.11)
3-methyl-1H-indole	TIP4P/2005	293.00	[4]	-20.51	(0.13)	-24.84	(0.09)	-24.03	(0.09)
3-methylphenol	3-methylphenol	298.00	[1]	-36.47	(0.27)	-36.55	(0.30)	-38.52	(0.30)
3-methylphenol	cyclohexane	298.00	[1]	-21.76	(0.14)	-22.00	(0.08)	-26.63	(0.08)
3-methylphenol	TIP3P	298.00	[1]	-25.66	(0.10)	-28.28	(0.12)	-28.22	(0.12)
3-methylphenol	TIP4P/2005	298.00	[1]	-23.55	(0.31)	-26.41	(0.16)	-28.11	(0.16)
3-methylpyridine	TIP3P	298.00	[1]	-11.71	(0.12)	-17.36	(0.12)	-19.00	(0.12)
3-methylpyridine	TIP4P/2005	298.00	[1]	-11.42	(0.08)	-18.06	(0.10)	-19.33	(0.10)

4-fluorophenol	TIP3P	298.15	[2,3]	-20.00	(0.09)	-20.76	(0.18)	-23.12	(0.18)
4-fluorophenol	TIP4P/2005	298.15	[2,3]	-17.91	(0.20)	-18.62	(0.30)	-24.15	(0.30)
4-methoxy-N,N-dimethylbenzamide	TIP3P	298.00	[1]	-33.92	(0.06)	-42.97	(0.14)	-47.23	(0.14)
4-methoxy-N,N-dimethylbenzamide	TIP4P/2005	298.00	[1]	-35.07	(0.23)	-44.96	(0.21)	-43.12	(0.21)
4-methyl-1H-imidazole	TIP3P	298.15	[2,3]	-32.64	(0.09)	-40.02	(0.12)	-42.86	(0.12)
4-methyl-1H-imidazole	TIP4P/2005	298.15	[2,3]	-33.49	(0.12)	-41.67	(0.16)	-42.50	(0.16)
4-methyl-1H-indole	cyclohexane	293.00	[4]	-19.95	(0.06)	-19.92	(0.03)	-21.46	(0.03)
4-methylphenol	pentan-1-ol	298.00	[1]	-33.90	(0.39)	-32.74	(0.31)	-37.48	(0.31)
4-methylphenol	benzene	298.00	[1]	-29.03	(0.12)	-29.14	(0.07)	-33.70	(0.07)
4-methylphenol	TIP3P	298.00	[1]	-20.44	(0.11)	-21.18	(0.05)	-23.46	(0.05)
4-methylphenol	TIP4P/2005	298.00	[1]	-18.74	(0.17)	-19.95	(0.25)	-23.51	(0.25)
4-methylpyridine	TIP3P	298.00	[1]	-13.22	(0.06)	-19.22	(0.16)	-21.07	(0.16)
4-methylpyridine	TIP4P/2005	298.00	[1]	-13.38	(0.16)	-20.36	(0.09)	-20.95	(0.09)
5-fluoracil	trichloromethane	298.00	[1]	-50.51	(0.19)	-58.89	(0.09)	-51.07	(0.09)
5-fluoracil	ethoxyethane	298.00	[1]	-50.01	(0.08)	-56.57	(0.18)	-63.12	(0.18)
5-fluoracil	ethyl acetate	298.00	[1]	-71.16	(0.14)	-86.02	(0.23)	-71.93	(0.23)
acetaldehyde	trichloromethane	298.00	[1]	-16.65	(0.07)	-20.70	(0.08)	-17.58	(0.08)
acetaldehyde	ethoxyethane	298.00	[1]	-11.67	(0.09)	-13.60	(0.04)	-12.81	(0.04)
acetaldehyde	TIP3P	298.00	[1]	-13.70	(0.09)	-21.65	(0.12)	-14.01	(0.12)
acetaldehyde	TIP4P/2005	298.00	[1]	-14.37	(0.09)	-23.13	(0.17)	-14.31	(0.17)
acetamide	trichloromethane	298.00	[1]	-28.37	(0.08)	-34.15	(0.06)	-29.65	(0.06)
acetamide	TIP3P	298.00	[1]	-36.86	(0.04)	-46.89	(0.06)	-43.21	(0.06)
acetamide	TIP4P/2005	298.00	[1]	-38.75	(0.14)	-49.89	(0.12)	-44.42	(0.12)
acetone	hexafluorobenzene	298.00	[1]	-18.74	(0.10)	-21.27	(0.11)	-19.52	(0.11)
acetone	chlorobenzene	298.00	[1]	-16.70	(0.04)	-19.75	(0.12)	-18.68	(0.12)
acetone	toluene	298.00	[1]	-14.95	(0.08)	-16.80	(0.05)	-16.38	(0.05)
acetone	cyclohexane	298.00	[1]	-10.01	(0.07)	-10.12	(0.05)	-10.60	(0.05)
acetone	trichloromethane	298.00	[1]	-20.90	(0.08)	-25.70	(0.10)	-22.90	(0.10)
acetone	benzene	298.00	[1]	-16.03	(0.04)	-17.95	(0.08)	-16.92	(0.08)
acetone	TIP3P	298.00	[1]	-14.43	(0.05)	-23.36	(0.07)	-15.17	(0.07)
acetone	TIP4P/2005	298.00	[1]	-15.35	(0.07)	-25.37	(0.14)	-15.48	(0.14)
acetonitrile	trichloromethane	298.00	[1]	-22.33	(0.08)	-26.90	(0.11)	-22.35	(0.11)
acetonitrile	cyclohexane	298.00	[1]	-10.82	(0.04)	-10.77	(0.06)	-7.99	(0.06)
acetonitrile	ethoxyethane	298.00	[1]	-17.88	(0.03)	-20.84	(0.08)	-18.23	(0.08)
acetonitrile	acetonitrile	298.00	[1]	-23.45	(0.08)	-29.06	(0.13)	-24.52	(0.13)
acetonitrile	TIP3P	298.00	[1]	-18.45	(0.11)	-27.04	(0.06)	-16.89	(0.06)
acetonitrile	TIP4P/2005	298.00	[1]	-18.64	(0.05)	-27.79	(0.11)	-18.70	(0.10)
aniline	TIP3P	298.00	[1]	-13.23	(0.13)	-16.05	(0.11)	-17.78	(0.11)
aniline	TIP4P/2005	298.00	[1]	-13.19	(0.08)	-16.20	(0.17)	-15.95	(0.17)
aniline	bromobenzene	298.00	[1]	-22.70	(0.09)	-23.62	(0.05)	-27.85	(0.05)
aniline	butyl acetate	298.00	[1]	-25.49	(0.16)	-27.15	(0.15)	-28.24	(0.15)
aniline	carbon tetrachloride	298.00	[1]	-20.49	(0.07)	-21.23	(0.09)	-25.09	(0.09)
aniline	aniline	298.00	[1]	-24.16	(0.09)	-24.89	(0.19)	-28.20	(0.19)
anisole	cyclohexane	298.00	[1]	-21.28	(0.14)	-21.07	(0.05)	-22.10	(0.05)
anisole	anisole	298.00	[1]	-25.02	(0.10)	-25.54	(0.14)	-25.64	(0.14)
anisole	ethoxyethane	298.00	[1]	-24.23	(0.12)	-24.54	(0.09)	-24.78	(0.09)
benzamide	benzene	298.00	[1]	-37.58	(0.07)	-39.78	(0.02)	-41.54	(0.02)
benzamide	trichloromethane	298.00	[1]	-41.51	(0.08)	-46.39	(0.17)	-44.56	(0.17)
benzamide	ethoxyethane	298.00	[1]	-36.32	(0.10)	-38.90	(0.05)	-44.36	(0.05)
benzamide	TIP3P	298.00	[1]	-35.18	(0.13)	-43.88	(0.08)	-41.10	(0.08)
benzamide	TIP4P/2005	298.00	[1]	-35.19	(0.12)	-45.06	(0.10)	-41.68	(0.10)
benzene	cyclohexane	298.00	[1]	-13.79	(0.05)	-13.85	(0.05)	-16.95	(0.05)
benzene	benzene	298.00	[1]	-16.10	(0.07)	-16.32	(0.06)	-19.56	(0.06)
benzene	ethoxyethane	298.00	[1]	-15.56	(0.02)	-16.13	(0.06)	-19.07	(0.06)
benzene	TIP3P	298.00	[1]	-3.44	(0.05)	-6.14	(0.07)	-3.72	(0.07)
benzene	TIP4P/2005	298.00	[1]	-2.59	(0.17)	-5.76	(0.15)	-3.67	(0.15)
benzonitrile	cyclohexane	298.00	[1]	-24.73	(0.14)	-24.66	(0.12)	-24.03	(0.12)
benzonitrile	carbon tetrachloride	298.00	[1]	-26.15	(0.08)	-27.19	(0.13)	-26.47	(0.13)
benzonitrile	benzonitrile	298.00	[1]	-34.57	(0.16)	-38.30	(0.27)	-35.35	(0.27)
benzonitrile	TIP3P	298.00	[1]	-19.53	(0.06)	-26.56	(0.07)	-15.59	(0.07)
benzonitrile	TIP4P/2005	298.00	[1]	-18.72	(0.13)	-26.35	(0.08)	-17.15	(0.08)
bromobenzene	trichloromethane	298.00	[1]	-26.45	(0.04)	-26.82	(0.06)	-28.55	(0.06)
bromobenzene	cyclohexane	298.00	[1]	-22.84	(0.15)	-22.41	(0.13)	-24.96	(0.13)
bromobenzene	ethoxyethane	298.00	[1]	-25.02	(0.13)	-25.33	(0.08)	-27.32	(0.08)
bromobenzene	bromobenzene	298.00	[1]	-24.35	(0.15)	-25.17	(0.17)	-27.28	(0.17)
bromobenzene	carbon tetrachloride	298.00	[1]	-23.31	(0.07)	-23.45	(0.08)	-25.59	(0.08)
bromobenzene	TIP3P	298.00	[1]	-3.78	(0.08)	-5.38	(0.08)	-3.17	(0.08)
bromobenzene	TIP4P/2005	298.00	[1]	-2.42	(0.07)	-3.81	(0.14)	-3.28	(0.14)
bromoethane	bromoethane	298.00	[1]	-14.60	(0.05)	-16.03	(0.13)	-17.53	(0.13)
bromoethane	TIP3P	298.00	[1]	0.17	(0.11)	-2.95	(0.05)	-3.20	(0.05)
bromoethane	TIP4P/2005	298.00	[1]	0.66	(0.11)	-1.87	(0.11)	-3.71	(0.11)
but-1-ene	carbon tetrachloride	298.00	[1]	-8.59	(0.08)	-8.69	(0.16)	-11.58	(0.16)
but-1-ene	TIP3P	298.00	[1]	7.36	(0.10)	5.19	(0.04)	5.39	(0.04)
but-1-ene	TIP4P/2005	298.00	[1]	8.67	(0.11)	6.32	(0.16)	4.67	(0.16)
but-1-yne	TIP3P	298.00	[1]	-3.29	(0.09)	-5.80	(0.03)	-1.30	(0.03)
but-1-yne	TIP4P/2005	298.00	[1]	-2.85	(0.14)	-5.56	(0.09)	-1.15	(0.09)

buta-1,3-diene	octan-1-ol	298.00	[1]	-3.64	(0.22)	-4.01	(0.14)	-6.12	(0.14)
buta-1,3-diene	TIP3P	298.00	[1]	5.53	(0.05)	3.63	(0.13)	3.54	(0.13)
buta-1,3-diene	TIP4P/2005	298.00	[1]	7.14	(0.11)	5.64	(0.10)	3.86	(0.10)
butan-1-amine	phenylmethanol	298.00	[1]	-36.02	(0.34)	-36.68	(0.36)	-25.65	(0.36)
butan-1-amine	hexafluorobenzene	298.00	[1]	-21.73	(0.18)	-21.33	(0.08)	-23.69	(0.08)
butan-1-amine	TIP3P	298.00	[1]	-27.01	(0.06)	-27.93	(0.12)	-21.20	(0.12)
butan-1-amine	TIP4P/2005	298.00	[1]	-31.88	(0.10)	-33.16	(0.26)	-22.42	(0.26)
butan-2-one	acetonitrile	298.00	[1]	-19.82	(0.11)	-21.44	(0.14)	-21.24	(0.14)
butan-2-one	benzonitrile	298.00	[1]	-18.17	(0.15)	-21.38	(0.39)	-20.60	(0.39)
butan-2-one	1-phenylethan-1-one	298.00	[1]	-17.85	(0.12)	-20.59	(0.15)	-20.11	(0.15)
butan-2-one	1-chlorohexane	298.00	[1]	-17.16	(0.08)	-19.61	(0.07)	-20.15	(0.07)
butan-2-one	3-methylphenol	298.00	[1]	-23.07	(0.24)	-31.18	(0.31)	-27.68	(0.31)
butanal	2-methylpropan-1-ol	298.00	[1]	-18.44	(0.13)	-21.48	(0.35)	-21.67	(0.35)
butanal	TIP3P	298.00	[1]	-13.09	(0.11)	-20.95	(0.05)	-13.85	(0.05)
butanal	TIP4P/2005	298.00	[1]	-13.63	(0.13)	-22.12	(0.14)	-14.01	(0.14)
butane	cyclohexane	298.00	[1]	-9.44	(0.07)	-9.34	(0.06)	-13.21	(0.06)
butane	TIP3P	298.00	[1]	10.87	(0.06)	10.73	(0.11)	8.69	(0.11)
butane	TIP4P/2005	298.00	[1]	11.80	(0.10)	11.91	(0.08)	8.49	(0.08)
butanenitrile	TIP3P	298.00	[1]	-17.12	(0.08)	-25.63	(0.14)	-15.54	(0.14)
butanenitrile	TIP4P/2005	298.00	[1]	-17.34	(0.17)	-26.32	(0.26)	-17.27	(0.26)
carbon tetrachloride	carbon tetrachloride	298.00	[1]	-18.26	(0.12)	-18.69	(0.24)	-18.53	(0.24)
carbon tetrafluoride	TIP3P	298.00	[1]	10.83	(0.05)	10.47	(0.03)	10.53	(0.03)
carbon tetrafluoride	TIP4P/2005	298.00	[1]	11.33	(0.08)	11.29	(0.09)	8.66	(0.09)
chlorobenzene	cyclohexane	298.00	[1]	-19.23	(0.09)	-18.83	(0.10)	-21.62	(0.10)
chlorobenzene	ethoxyethane	298.00	[1]	-21.66	(0.04)	-21.91	(0.08)	-24.15	(0.08)
chlorobenzene	carbon tetrachloride	298.00	[1]	-19.83	(0.10)	-20.24	(0.04)	-22.69	(0.04)
chlorobenzene	TIP3P	298.00	[1]	-2.12	(0.08)	-3.48	(0.05)	-1.89	(0.05)
chlorobenzene	TIP4P/2005	298.00	[1]	-0.41	(0.12)	-1.40	(0.16)	-3.54	(0.16)
cyclohexane	cyclohexane	298.00	[1]	-18.61	(0.11)	-18.44	(0.05)	-18.44	(0.05)
cyclohexane	TIP3P	298.00	[1]	6.72	(0.05)	6.54	(0.06)	4.71	(0.06)
cyclohexane	TIP4P/2005	298.00	[1]	7.31	(0.10)	7.32	(0.09)	5.22	(0.09)
cyclopentane	TIP3P	298.00	[1]	6.97	(0.06)	6.99	(0.02)	5.00	(0.02)
cyclopentane	TIP4P/2005	298.00	[1]	7.37	(0.08)	7.30	(0.09)	5.32	(0.09)
diiodomethane	TIP3P	298.00	[1]			-5.58	(0.12)	-10.75	(0.12)
diiodomethane	TIP4P/2005	298.00	[1]			-5.02	(0.16)	-11.31	(0.16)
ethanamine	bromobenzene	298.00	[1]	-13.20	(0.09)	-13.88	(0.13)	-15.29	(0.13)
ethanamine	cyclohexane	298.00	[1]	-7.88	(0.03)	-7.80	(0.04)	-11.11	(0.04)
ethanamine	ethoxyethane	298.00	[1]	-12.98	(0.09)	-13.19	(0.05)	-15.38	(0.05)
ethanamine	iodobenzene	298.00	[1]			-13.59	(0.07)	-15.11	(0.07)
ethanamine	TIP3P	298.00	[1]	-23.50	(0.07)	-24.03	(0.08)	-19.91	(0.08)
ethanamine	TIP4P/2005	298.00	[1]	-27.94	(0.04)	-28.17	(0.12)	-20.07	(0.12)
ethane	TIP3P	298.00	[1]	10.47	(0.08)	10.41	(0.04)	8.79	(0.04)
ethane	TIP4P/2005	298.00	[1]	11.01	(0.11)	10.69	(0.07)	8.06	(0.07)
ethane-1,2-diol	octan-1-ol	298.00	[1]	-39.74	(0.48)	-40.60	(0.32)	-31.70	(0.32)
ethane-1,2-diol	TIP3P	298.00	[1]	-48.68	(0.15)	-52.72	(0.10)	-43.05	(0.10)
ethane-1,2-diol	TIP4P/2005	298.00	[1]	-51.07	(0.17)	-55.64	(0.20)	-42.94	(0.20)
ethanol	ethanol	298.00	[1]	-23.39	(0.05)	-23.94	(0.31)	-20.19	(0.31)
ethanol	benzene	298.00	[1]	-14.76	(0.05)	-15.09	(0.06)	-16.12	(0.06)
ethanol	cyclohexane	298.00	[1]	-7.69	(0.06)	-7.57	(0.03)	-10.28	(0.03)
ethanol	phenylmethanol	298.00	[1]	-21.55	(0.31)	-21.83	(0.38)	-18.68	(0.38)
ethanol	propan-2-ol	298.00	[1]	-22.65	(0.43)	-23.24	(0.44)	-19.43	(0.44)
ethanol	butan-1-ol	298.00	[1]	-22.67	(0.41)	-22.99	(0.48)	-19.60	(0.38)
ethanol	ethoxyethane	298.00	[1]	-14.89	(0.07)	-15.69	(0.06)	-18.39	(0.06)
ethanol	3-methylphenol	298.00	[1]	-23.08	(0.28)	-24.02	(0.33)	-19.46	(0.33)
ethanol	trichloromethane	298.00	[1]	-15.44	(0.03)	-16.72	(0.03)	-16.87	(0.03)
ethanol	chlorobenzene	298.00	[1]	-13.24	(0.11)	-14.07	(0.11)	-14.82	(0.11)
ethanol	acetonitrile	298.00	[1]	-19.79	(0.06)	-22.30	(0.04)	-20.46	(0.04)
ethanol	benzonitrile	298.00	[1]	-17.23	(0.06)	-20.30	(0.34)	-19.14	(0.34)
ethanol	bromobenzene	298.00	[1]	-13.01	(0.08)	-13.99	(0.06)	-15.57	(0.06)
ethanol	ethyl acetate	298.00	[1]	-22.76	(0.12)	-25.91	(0.04)	-20.26	(0.04)
ethanol	fluorobenzene	298.00	[1]	-13.80	(0.06)	-14.88	(0.08)	-16.25	(0.08)
ethanol	iodobenzene	298.00	[1]			-13.57	(0.11)	-15.01	(0.11)
ethanol	TIP3P	298.00	[1]	-19.16	(0.09)	-20.53	(0.06)	-17.23	(0.06)
ethanol	TIP4P/2005	298.00	[1]	-20.03	(0.09)	-21.76	(0.10)	-17.27	(0.10)
ethene	TIP3P	298.00	[1]	7.27	(0.09)	6.02	(0.04)	6.82	(0.04)
ethene	TIP4P/2005	298.00	[1]	8.51	(0.05)	7.09	(0.13)	7.60	(0.13)
ethoxybenzene	cyclohexane	298.00	[1]	-24.35	(0.17)	-24.05	(0.06)	-25.26	(0.06)
ethoxybenzene	ethoxybenzene	298.00	[1]	-27.11	(0.07)	-27.20	(0.06)	-27.79	(0.06)
ethoxybenzene	TIP3P	298.00	[1]	-7.64	(0.03)	-10.35	(0.15)	-10.42	(0.15)
ethoxybenzene	TIP4P/2005	298.00	[1]	-6.52	(0.13)	-10.03	(0.27)	-11.37	(0.27)
ethoxyethane	octan-1-ol	298.00	[1]	-11.78	(0.21)	-10.94	(0.25)	-11.95	(0.25)
ethoxyethane	ethoxyethane	298.00	[1]	-15.21	(0.12)	-14.98	(0.06)	-15.27	(0.06)
ethoxyethane	TIP3P	298.00	[1]	0.27	(0.07)	-1.13	(0.04)	-4.70	(0.04)
ethoxyethane	TIP4P/2005	298.00	[1]	-0.29	(0.16)	-1.62	(0.07)	-5.26	(0.07)
ethyl acetate	bromobenzene	298.00	[1]	-23.12	(0.09)	-25.13	(0.13)	-22.63	(0.13)
ethyl acetate	toluene	298.00	[1]	-22.02	(0.18)	-23.14	(0.12)	-21.78	(0.12)

ethyl acetate	chlorobenzene	298.00	[1]	-23.06	(0.15)	-24.91	(0.11)	-22.88	(0.11)
ethyl acetate	ethyl acetate	298.00	[1]	-22.90	(0.05)	-24.10	(0.09)	-21.96	(0.09)
ethyl acetate	TIP3P	298.00	[1]	-17.13	(0.06)	-22.73	(0.12)	-13.62	(0.12)
ethyl acetate	TIP4P/2005	298.00	[1]	-19.08	(0.10)	-25.74	(0.26)	-11.83	(0.26)
ethyl formate	TIP3P	298.00	[1]	-19.31	(0.22)	-34.40	(0.13)	-10.20	(0.13)
ethyl formate	TIP4P/2005	298.00	[1]	-25.72	(0.18)	-31.11	(0.47)	-15.22	(0.47)
ethyne	TIP3P	298.00	[1]	-5.67	(0.09)	-7.40	(0.07)	1.22	(0.07)
ethyne	TIP4P/2005	298.00	[1]	-5.29	(0.08)	-7.14	(0.14)	1.32	(0.14)
fluorobenzene	carbon tetrachloride	298.00	[1]	-15.42	(0.05)	-15.89	(0.07)	-18.83	(0.07)
fluorobenzene	cyclohexane	298.00	[1]	-14.20	(0.07)	-14.12	(0.15)	-17.50	(0.15)
fluorobenzene	TIP3P	298.00	[1]	-0.53	(0.04)	-3.19	(0.13)	-1.82	(0.13)
fluorobenzene	TIP4P/2005	298.00	[1]	1.11	(0.08)	-1.67	(0.11)	-2.96	(0.11)
formaldehyde	phenylmethanol	298.00	[1]	-10.34	(0.33)	-14.06	(0.36)	-12.11	(0.36)
formamide	ethoxyethane	298.00	[1]	-15.24	(0.05)	-17.37	(0.06)	-19.08	(0.06)
hex-1-ene	TIP3P	298.00	[1]	8.19	(0.08)	6.04	(0.07)	5.14	(0.07)
hex-1-ene	TIP4P/2005	298.00	[1]	9.70	(0.15)	7.23	(0.18)	5.77	(0.18)
hexane	TIP3P	298.00	[1]	11.90	(0.07)	11.76	(0.11)	9.21	(0.11)
hexane	TIP4P/2005	298.00	[1]	13.23	(0.13)	13.09	(0.08)	9.66	(0.08)
iodobenzene	cyclohexane	298.00	[1]			-24.76	(0.08)	-27.61	(0.08)
iodobenzene	trichloromethane	298.00	[1]			-28.01	(0.10)	-30.42	(0.10)
iodobenzene	carbon tetrachloride	298.00	[1]			-24.99	(0.10)	-28.32	(0.10)
iodobenzene	ethoxyethane	298.00	[1]			-24.65	(0.09)	-26.82	(0.09)
iodobenzene	TIP3P	298.00	[1]			-4.84	(0.08)	-5.61	(0.08)
iodobenzene	TIP4P/2005	298.00	[1]			-3.46	(0.19)	-6.09	(0.19)
iodoethane	TIP3P	298.00	[1]			-0.05	(0.08)	-4.12	(0.08)
iodoethane	TIP4P/2005	298.00	[1]			0.56	(0.19)	-4.19	(0.19)
iodomethane	ethoxyethane	298.00	[1]			-13.24	(0.01)	-13.96	(0.02)
iodomethane	TIP3P	298.00	[1]			-0.39	(0.12)	-3.33	(0.12)
iodomethane	TIP4P/2005	298.00	[1]			0.33	(0.17)	-2.98	(0.17)
methanamine	benzene	298.00	[1]	-9.76	(0.06)	-9.84	(0.11)	-10.65	(0.11)
methanamine	trichloromethane	298.00	[1]	-12.59	(0.06)	-13.49	(0.07)	-12.82	(0.07)
methanamine	ethoxyethane	298.00	[1]	-8.96	(0.05)	-9.04	(0.07)	-10.58	(0.07)
methanamine	toluene	298.00	[1]	-9.04	(0.06)	-9.00	(0.07)	-10.30	(0.07)
methanamine	carbon tetrachloride	298.00	[1]	-5.16	(0.10)	-5.72	(0.04)	-7.59	(0.04)
methanamine	TIP3P	298.00	[1]	-20.39	(0.08)	-20.00	(0.06)	-17.81	(0.06)
methanamine	TIP4P/2005	298.00	[1]	-23.65	(0.14)	-23.34	(0.04)	-17.27	(0.04)
methanol	butan-1-ol	298.00	[1]	-20.37	(0.27)	-20.98	(0.47)	-14.59	(0.37)
methanol	ethoxyethane	298.00	[1]	-12.31	(0.04)	-13.08	(0.05)	-14.87	(0.05)
methanol	benzene	298.00	[1]	-12.04	(0.05)	-12.46	(0.03)	-12.35	(0.03)
methanol	cyclohexane	298.00	[1]	-3.69	(0.03)	-3.67	(0.05)	-5.81	(0.05)
methanol	trichloromethane	298.00	[1]	-12.25	(0.06)	-13.30	(0.07)	-13.03	(0.07)
methanol	chlorobenzene	298.00	[1]	-10.20	(0.06)	-10.94	(0.07)	-11.46	(0.07)
methanol	bromobenzene	298.00	[1]	-9.97	(0.06)	-10.80	(0.04)	-11.32	(0.04)
methanol	ethyl acetate	298.00	[1]	-20.94	(0.12)	-23.88	(0.11)	-17.24	(0.11)
methanol	iodobenzene	298.00	[1]			-10.39	(0.10)	-10.53	(0.10)
methanol	TIP3P	298.00	[1]	-21.84	(0.08)	-21.62	(0.07)	-17.75	(0.07)
methanol	TIP4P/2005	298.00	[1]	-22.49	(0.11)	-22.09	(0.05)	-17.27	(0.05)
methoxymethane	TIP3P	298.00	[1]	-2.22	(0.04)	-2.45	(0.05)	-4.48	(0.05)
methoxymethane	TIP4P/2005	298.00	[1]	-1.89	(0.08)	-2.52	(0.11)	-4.04	(0.11)
methyl acetate	cyclohexane	298.00	[1]	-14.12	(0.11)	-14.32	(0.07)	-12.24	(0.07)
methyl acetate	benzene	298.00	[1]	-20.22	(0.06)	-21.20	(0.05)	-18.31	(0.05)
methyl acetate	hexafluorobenzene	298.00	[1]	-23.01	(0.08)	-24.14	(0.10)	-19.11	(0.10)
methyl acetate	TIP3P	298.00	[1]	-18.49	(0.09)	-23.55	(0.06)	-13.50	(0.06)
methyl acetate	TIP4P/2005	298.00	[1]	-20.12	(0.12)	-25.85	(0.16)	-11.04	(0.16)
methyl benzoate	TIP3P	298.00	[1]	-19.41	(0.11)	-23.51	(0.09)	-14.14	(0.09)
methyl benzoate	TIP4P/2005	298.00	[1]	-20.12	(0.09)	-24.38	(0.28)	-13.38	(0.28)
methyl formate	octan-1-ol	298.00	[1]	-13.91	(0.34)	-19.48	(0.45)	-11.56	(0.35)
methyl formate	TIP3P	298.00	[1]	-17.37	(0.06)	-21.51	(0.12)	-11.93	(0.11)
methyl formate	TIP4P/2005	298.00	[1]	-18.51	(0.10)	-23.29	(0.15)	-9.99	(0.15)
n-(3-hydroxyphenyl)acetamide	TIP3P	298.00	[1]	-58.82	(0.23)	-68.94	(0.11)	-66.09	(0.11)
n-(3-hydroxyphenyl)acetamide	TIP4P/2005	298.00	[1]	-59.42	(0.23)	-71.05	(0.24)	-64.74	(0.24)
n,n,4-trimethylbenzamide	TIP3P	298.00	[1]	-25.96	(0.12)	-35.89	(0.15)	-39.28	(0.15)
n,n,4-trimethylbenzamide	TIP4P/2005	298.00	[1]	-27.07	(0.16)	-37.85	(0.22)	-37.41	(0.22)
N,N-dimethylacetamide	trichloromethane	298.00	[1]	-34.81	(0.11)	-41.06	(0.03)	-36.93	(0.03)
N,N-dimethylacetamide	N,N-dimethylacetamide	298.00	[1]	-28.46	(0.21)	-31.42	(0.14)	-31.24	(0.14)
N,N-dimethylformamide	cyclohexane	298.00	[1]	-15.71	(0.07)	-15.83	(0.08)	-15.85	(0.08)
N,N-dimethylformamide	ethoxyethane	298.00	[1]	-22.99	(0.15)	-25.81	(0.07)	-25.45	(0.07)
n-methylacetamide	TIP3P	298.00	[1]	-32.02	(0.15)	-44.20	(0.15)	-43.19	(0.15)
n-methylacetamide	TIP4P/2005	298.00	[1]	-34.61	(0.04)	-48.53	(0.17)	-42.03	(0.17)
n-methylaniline	TIP3P	298.00	[1]	-13.73	(0.05)	-17.10	(0.06)	-22.50	(0.06)
n-methylaniline	TIP4P/2005	298.00	[1]	-13.10	(0.08)	-16.86	(0.14)	-21.46	(0.14)
octane	acetonitrile	298.00	[1]	-15.37	(0.08)	-7.72	(0.08)	-16.47	(0.08)
octane	benzonitrile	298.00	[1]	-16.21	(0.24)	-11.82	(0.39)	-20.41	(0.39)
pent-1-ene	TIP3P	298.00	[1]	8.24	(0.07)	6.83	(0.07)	5.94	(0.07)
pent-1-ene	TIP4P/2005	298.00	[1]	9.85	(0.13)	8.04	(0.13)	6.34	(0.13)
pent-1-yne	TIP3P	298.00	[1]	-3.69	(0.03)	-6.26	(0.12)	-1.25	(0.12)

pent-1-yne	TIP4P/2005	298.00	[1]	-3.00	(0.19)	-5.62	(0.08)	-1.72	(0.08)
pentane	cyclohexane	298.00	[1]	-12.88	(0.11)	-12.71	(0.08)	-17.20	(0.08)
pentane	TIP3P	298.00	[1]	11.40	(0.05)	11.27	(0.11)	8.90	(0.11)
pentane	TIP4P/2005	298.00	[1]	12.51	(0.09)	12.43	(0.07)	9.21	(0.07)
phenol	toluene	298.00	[1]	-25.20	(0.09)	-25.59	(0.10)	-29.03	(0.10)
phenol	cyclohexane	298.00	[1]	-18.50	(0.14)	-18.67	(0.05)	-21.98	(0.05)
phenol	benzene	298.00	[1]	-26.29	(0.07)	-26.61	(0.12)	-29.87	(0.12)
phenol	pentan-1-ol	298.00	[1]	-30.38	(0.35)	-31.20	(0.37)	-33.67	(0.37)
phenol	ethoxyethane	298.00	[1]	-26.46	(0.06)	-27.26	(0.12)	-32.77	(0.12)
phenol	2-propan-2-yloxypropane	298.00	[1]	-29.79	(0.09)	-30.21	(0.14)	-37.17	(0.14)
phenol	trichloromethane	298.00	[1]	-26.78	(0.08)	-27.65	(0.12)	-30.21	(0.12)
phenol	tetrachloroethene	298.00	[1]	-19.78	(0.07)	-19.69	(0.09)	-23.12	(0.09)
phenol	bromobenzene	298.00	[1]	-24.52	(0.08)	-25.80	(0.20)	-28.94	(0.20)
phenol	ethyl acetate	298.00	[1]	-37.11	(0.12)	-39.12	(0.11)	-36.97	(0.11)
phenol	TIP3P	298.00	[1]	-21.78	(0.11)	-22.92	(0.06)	-24.39	(0.06)
phenol	TIP4P/2005	298.00	[1]	-20.48	(0.20)	-21.91	(0.07)	-24.55	(0.07)
phenyl formate	TIP3P	298.00	[1]	-25.18	(0.10)	-32.63	(0.10)	-16.84	(0.10)
phenyl formate	TIP4P/2005	298.00	[1]	-25.04	(0.05)	-32.70	(0.04)	-19.24	(0.04)
piperidine	2-methylpropan-1-ol	298.00	[1]	-27.15	(0.62)	-26.17	(0.44)	-25.00	(0.34)
propan-1-ol	TIP3P	298.00	[1]	-24.55	(0.11)	-26.81	(0.16)	-20.26	(0.16)
propan-1-ol	TIP4P/2005	298.00	[1]	-26.31	(0.11)	-28.97	(0.18)	-20.86	(0.18)
propan-2-ol	propan-2-ol	298.00	[1]	-26.80	(0.40)	-24.16	(0.21)	-22.82	(0.21)
propanal	TIP3P	298.00	[1]	-15.21	(0.12)	-23.59	(0.10)	-15.41	(0.10)
propanal	TIP4P/2005	298.00	[1]	-16.01	(0.09)	-25.34	(0.21)	-16.36	(0.21)
propane	cyclohexane	298.00	[1]	-5.97	(0.06)	-6.13	(0.08)	-9.43	(0.08)
propane	TIP3P	298.00	[1]	10.37	(0.06)	10.36	(0.05)	8.63	(0.05)
propane	TIP4P/2005	298.00	[1]	11.13	(0.07)	11.22	(0.08)	8.43	(0.08)
propanenitrile	TIP3P	298.00	[1]	-16.90	(0.12)	-25.43	(0.10)	-15.73	(0.10)
propanenitrile	TIP4P/2005	298.00	[1]	-17.19	(0.07)	-26.46	(0.09)	-17.26	(0.10)
propene	octan-1-ol	298.00	[1]	-1.58	(0.13)	-1.91	(0.21)	-3.53	(0.21)
propene	TIP3P	298.00	[1]	6.91	(0.06)	5.04	(0.05)	5.14	(0.05)
propene	TIP4P/2005	298.00	[1]	8.02	(0.08)	5.74	(0.09)	5.19	(0.08)
propyl acetate	carbon tetrachloride	298.00	[1]	-22.93	(0.13)	-23.87	(0.10)	-22.82	(0.10)
propyl acetate	ethylbenzene	298.00	[1]	-24.14	(0.05)	-25.29	(0.05)	-24.73	(0.05)
propyl acetate	hexafluorobenzene	298.00	[1]	-28.52	(0.13)	-29.75	(0.10)	-26.36	(0.10)
pyridine	benzene	298.00	[1]	-20.93	(0.08)	-21.82	(0.06)	-23.16	(0.06)
pyridine	trichloromethane	298.00	[1]	-24.41	(0.05)	-26.69	(0.04)	-27.00	(0.04)
pyridine	cyclohexane	298.00	[1]	-16.51	(0.04)	-16.72	(0.04)	-17.71	(0.04)
pyridine	ethoxyethane	298.00	[1]	-19.68	(0.08)	-20.86	(0.06)	-21.77	(0.06)
pyridine	toluene	298.00	[1]	-20.36	(0.07)	-21.29	(0.08)	-22.06	(0.08)
pyridine	2-methylpropan-1-ol	298.00	[1]	-18.70	(0.38)	-20.45	(0.16)	-23.21	(0.39)
pyridine	pyridine	298.00	[1]	-21.90	(0.08)	-23.60	(0.15)	-24.36	(0.15)
pyridine	TIP3P	298.00	[1]	-12.88	(0.10)	-17.89	(0.13)	-18.63	(0.13)
pyridine	TIP4P/2005	298.00	[1]	-12.92	(0.03)	-18.66	(0.07)	-18.27	(0.07)
TIP3P	toluene	298.00	[1]	-5.18	(0.03)	-6.68	(0.04)	-5.79	(0.04)
TIP4P/2005	toluene	298.00	[1]	-6.89	(0.04)	-8.73	(0.05)	-7.81	(0.05)
toluene	toluene	298.00	[1]	-18.54	(0.02)	-18.95	(0.03)	-23.14	(0.03)
toluene	3-methylphenol	298.00	[1]	-16.74	(0.16)	-14.68	(0.40)	-21.19	(0.40)
toluene	ethoxyethane	298.00	[1]	-18.84	(0.03)	-19.17	(0.02)	-22.78	(0.02)
toluene	acetonitrile	298.00	[1]	-18.08	(0.08)	-16.13	(0.10)	-21.13	(0.10)
toluene	benzonitrile	298.00	[1]	-16.41	(0.08)	-16.62	(0.15)	-22.23	(0.15)
toluene	TIP3P	298.00	[1]	-1.80	(0.10)	-4.81	(0.07)	-3.73	(0.07)
toluene	TIP4P/2005	298.00	[1]	-0.65	(0.18)	-4.16	(0.20)	-3.86	(0.20)
trans-pent-2-ene	carbon tetrachloride	298.00	[1]	-12.69	(0.05)	-13.04	(0.11)	-16.92	(0.11)
trans-pent-2-ene	TIP3P	298.00	[1]	9.73	(0.09)	8.30	(0.03)	5.95	(0.03)
trans-pent-2-ene	TIP4P/2005	298.00	[1]	11.11	(0.17)	9.86	(0.14)	5.08	(0.14)
tribromomethane	tribromomethane	298.00	[1]	-26.85	(0.27)	-27.82	(0.14)	-25.31	(0.14)
tribromomethane	TIP3P	298.00	[1]	-7.86	(0.12)	-9.70	(0.09)	-8.78	(0.09)
tribromomethane	TIP4P/2005	298.00	[1]	-9.05	(0.18)	-11.36	(0.17)	-9.61	(0.17)
trichloroethene	octan-1-ol	298.00	[1]	-15.48	(0.41)	-17.34	(0.29)	-16.52	(0.29)
trichloromethane	trichloromethane	298.00	[1]	-15.66	(0.10)	-15.64	(0.07)	-15.62	(0.07)
trichloromethane	TIP3P	298.00	[1]	0.79	(0.08)	0.08	(0.08)	-4.20	(0.08)
trichloromethane	TIP4P/2005	298.00	[1]	1.57	(0.03)	0.89	(0.12)	-4.72	(0.12)

2.2. Comparison of Different Force Fields for ΔG_{solv} Predictions for a Small Set of Test Systems

To highlight the impact of different force fields, we compare solvation free energy results obtained with GAFF/IPolQ-Mod+LJ-Fit for a small data set previously simulated [5] with force fields GAFF/RESP, GAFF/IPolQ-Mod, CGenFF and OPLS-AA. All simulations were conducted with solvent TIP3P at a temperature of T = 298 K and a pressure of p = 1 bar. Simulation protocols and parameters are identical to those described in section "Simulation Details"of the main paper, except for CGenFF where no long-range correction was applied as prescribed by the GROMACS manual [6]. Molecule topologies for CGenFF were created using the "CGenFF atom typer" [7,8] and the Python script "cgenff_charmm2gmx.py" [9], while "topolbuild 1.3" [10] was used for OPLS-AA. In Figure 1, simulation results are plotted *vs*. experimental data and numerical values are given in Table 3.

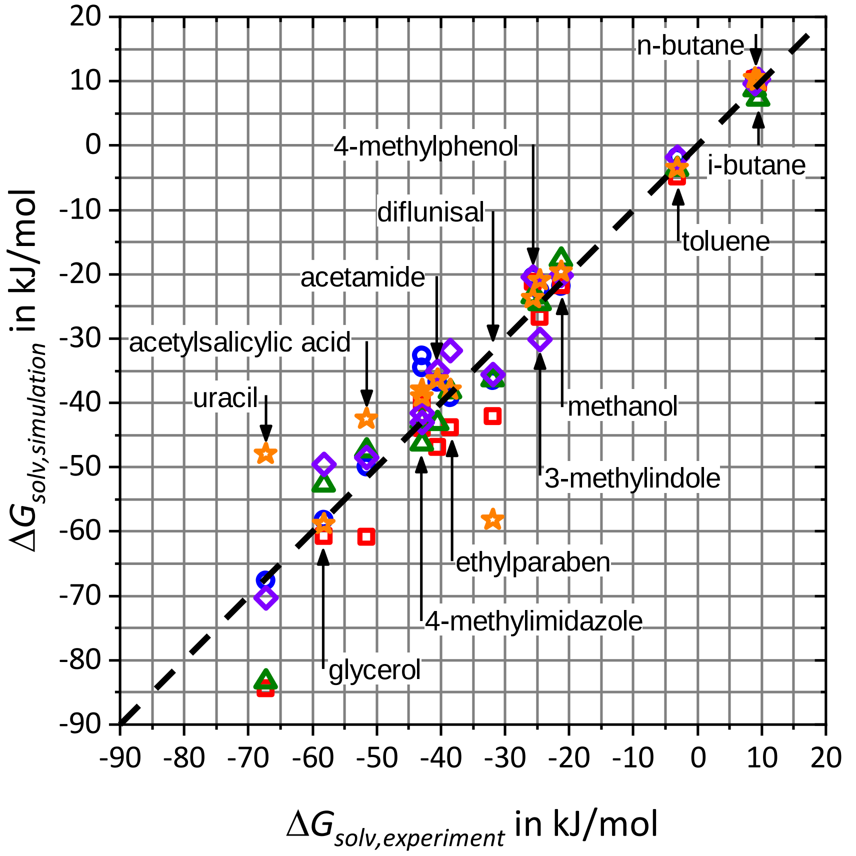

Figure 1. Simulated solvation free energies $\Delta G_{solv,simulation}$ *vs*. experimental data $\Delta G_{solv,experiment}$ for different force fields at T = 298 K and p = 1 bar. Results with GAFF/RESP are marked by blue circles, GAFF/IPolQ-Mod by red squares, GAFF/IPolQ-Mod+LJ-Fit by green triangles, CGenFF by violet diamonds and OPLS-AA by orange stars. The given compound names refer to the solutes and TIP3P was used as solvent in all cases.

It has to be stressed that the evaluation of such a small data set does not allow for a final assessment of force field quality, but merely demonstrates the impact different models may have on the description of solvation free energies.

Table 3: Comparison of simulation results for solvation free energies ΔG_{solv} obtained with different force fields, i.e. GAFF with default RESP and IPolQ-Mod partial charges as well as our newly derived parameters labelled GAFF/IPolQ-Mod+LJ-Fit; furthermore CGenFF and OPLS-AA. All simulations were conducted at a temperature of T = 298 K and a pressure of p = 1 bar with solvent TIP3P. The solvation free energies with corresponding statistical uncertainties, both given in kJ/mol, are followed by the absolute relative deviations (rel. dev.) referring to the reference data $\Delta G_{solv,ref}$. Compounds considered during the parameter fitting of GAFF/IPolQ-Mod+LJ-Fit are labelled as "fit".

**Solute**	**$\Delta G_{solv,ref}$**	**Source**	**GAFF/RESP**			**GAFF/IPolQ-Mod**			**GAFF/IPolQ-Mod+LJ-Fit**				**CGenFF**			**OPLS-AA**		
			**ΔG_{solv}**	**$\delta\Delta G_{solv}$**	**rel. dev.**	**ΔG_{solv}**	**$\delta\Delta G_{solv}$**	**rel. dev.**	**ΔG_{solv}**	**$\delta\Delta G_{solv}$**	**rel. dev.**		**ΔG_{solv}**	**$\delta\Delta G_{solv}$**	**rel. dev.**	**ΔG_{solv}**	**$\delta\Delta G_{solv}$**	**rel. dev.**
3-methylindole	-24.60	[11]	-22.58	(0.09)	8.22%	-26.73	(0.10)	8.64%	-24.58	(0.09)	**0.08%**	fit	-30.21	(0.11)	22.78%	-20.98	(0.15)	14.74%
4-methylimidazole (δ-prot.)	-42.97	[11]	-34.56	(0.17)	19.57%	-44.02	(0.11)	2.45%	-46.38	(0.14)	7.93%		-43.06	(0.11)	**0.21%**	-37.96	(0.14)	11.66%
4-methylimidazole	-42.97	[11]	-32.64	(0.09)	24.04%	-40.02	(0.12)	6.86%	-42.86	(0.09)	**0.25%**	fit	-41.63	(0.04)	3.11%	-39.00	(0.25)	9.23%
4-methylphenol	-25.56	[11]	-20.44	(0.11)	20.04%	-21.18	(0.05)	17.15%	-23.46	(0.05)	8.23%	fit	-20.39	(0.10)	20.22%	-23.80	(0.13)	**6.88%**
acetamide	-40.50	[11]	-36.86	(0.04)	8.99%	-46.89	(0.06)	15.77%	-43.21	(0.17)	**6.70%**	fit	-35.07	(0.16)	13.40%	-36.33	(0.10)	10.29%
acetylsalicylic acid	-51.59	[12]	-49.96	(0.13)	**3.16%**	-60.83	(0.14)	17.92%	-47.44	(0.08)	8.04%		-48.51	(0.20)	5.97%	-42.47	(0.32)	17.67%
diflunisal	-31.92	[12]	-36.53	(0.28)	14.42%	-42.07	(0.37)	31.79%	-36.41	(0.26)	14.07%		-35.64	(0.36)	**11.64%**	-58.25	(0.53)	82.46%
ethylparaben	-38.49	[12]	-39.15	(0.24)	1.69%	-43.84	(0.09)	13.88%	-38.27	(0.14)	**0.59%**		-31.93	(0.08)	17.06%	-38.05	(0.09)	1.15%
glycerol	-58.24	[12]	-58.17	(0.09)	**0.12%**	-60.70	(0.13)	4.22%	-52.69	(0.19)	9.53%		-49.52	(0.15)	14.98%	-58.93	(0.12)	1.18%
i-butane	9.54	[11]	9.79	(0.09)	2.63%	9.62	(0.10)	**0.80%**	7.20	(0.08)	24.55%		10.23	(0.13)	7.23%	10.07	(0.12)	5.52%
methanol	-21.17	[11]	-21.83	(0.14)	3.12%	-21.72	(0.09)	**2.61%**	-17.75	(0.04)	16.18%	fit	-20.15	(0.07)	4.82%	-19.58	(0.06)	7.54%
n-butane	9.00	[11]	10.41	(0.08)	15.75%	10.28	(0.09)	14.29%	8.69	(0.10)	**3.45%**	fit	9.61	(0.03)	6.84%	10.41	(0.07)	15.75%
toluene	-3.18	[11]	-1.80	(0.10)	43.39%	-4.81	(0.07)	51.27%	-3.73	(0.13)	17.36%	fit	-1.76	(0.04)	44.76%	-3.54	(0.15)	**11.24%**
uracil	-67.20	[12]	-67.69	(0.09)	**0.73%**	-84.44	(0.20)	25.67%	-83.29	(0.08)	23.95%		-70.27	(0.05)	4.58%	-48.03	(0.09)	28.53%

2.3. Refitting Data Set: Densities

Table 4: Simulation results for densities ρ in kg/m^3 from the refitting data set. The first column marks the compound, followed by the temperature T in K and the source for the experimental reference data. This is ensued by simulation results for the model parameter sets, whereas statistical uncertainties are given in brackets.

Compound	T	Source	GAFF/RESP		GAFF/IPolQ-Mod		GAFF/IPolQ-Mod+LJ-Fit	
			ρ	$\delta\rho$	ρ	$\delta\rho$	ρ	$\delta\rho$
1,1,1,3,3,3-hexafluoro-propan-2-ol	298.15	[13]	1576.08	(0.73)	1606.23	(0.62)	1506.22	(0.67)
1,1,1,3,3,3-hexafluoro-propan-2-ol	313.15	[13]	1539.00	(0.77)	1573.93	(1.10)	1481.02	(0.53)
1,1,1,3,3,3-hexafluoro-propan-2-ol	323.15	[13]	1517.98	(0.41)	1553.22	(1.20)	1463.91	(0.69)
1,1-dimethyl-1,3-butadien	198.15	[13]	786.29	(0.23)	791.91	(0.41)	779.06	(0.20)
1,1-dimethyl-1,3-butadien	248.15	[13]	730.24	(0.20)	736.55	(0.38)	735.26	(0.22)
1,1-dimethyl-1,3-butadien	298.15	[13]	670.42	(0.20)	677.34	(0.36)	691.44	(0.35)
1,2-dimethylimidazole	289.25	[14]	1008.72	(0.28)	1038.45	(0.86)	1003.17	(1.10)
1,4-dioxane	288.15	[13]	1095.00	(0.76)	1097.49	(0.78)	1078.61	(0.72)
1,4-dioxane	295.15	[13]	1087.10	(0.76)	1090.98	(0.65)	1070.70	(0.70)
1,4-dioxane	298.15	[13]	1084.40	(0.30)	1087.23	(0.40)	1065.89	(0.38)
1,4-dioxane	303.15	[13]	1078.87	(0.24)	1081.00	(0.45)	1059.16	(0.34)
1,4-dioxane	313.15	[13]	1068.04	(0.92)	1070.76	(0.82)	1045.21	(0.85)
1-ethyl-2-methylimidazole	294.95	[14]	964.31	(0.32)	989.52	(0.31)	966.90	(0.41)
1H-imidazole	368.15	[14]	1099.42	(0.58)	1136.16	(0.44)	1045.25	(0.36)
1H-imidazole	383.15	[14]	1085.51	(0.26)	1123.67	(0.42)	1033.68	(0.53)
1H-imidazole	426.15	[14]	1044.87	(0.41)	1086.97	(0.43)	1000.66	(0.43)
1H-imidazole	478.15	[14]	996.22	(0.41)	1044.78	(0.39)	958.76	(0.23)
1H-pyrrole	293.15	[13]	1012.19	(0.34)	1026.22	(1.30)	978.74	(0.23)
1H-pyrrole	288.15	[13]	1016.73	(0.90)	1029.69	(0.41)	983.17	(0.19)
1-isoamyl imidazole	292.85	[14]	945.79	(0.41)	964.52	(0.83)	947.13	(0.62)
1-methyl-1H-imidazole	293.65	[14]	1067.31	(0.27)	1103.96	(0.95)	1056.62	(0.34)
1-propyl-1H-imidazole	292.75	[14]	978.94	(0.79)	1003.80	(1.00)	983.79	(0.30)
2,2,2-trifluoroethan-1-ol	298.15	[13]	1348.58	(1.00)	1382.33	(1.40)	1308.36	(0.42)
2,4-dimethyl-1H-pyrrole	293.15	[13]	924.52	(0.96)	932.76	(1.50)	904.58	(0.29)
2-methylaniline	293.15	[13]	997.08	(0.54)	1013.04	(0.94)	965.64	(0.81)
2-methylaniline	308.15	[13]	980.80	(0.39)	997.58	(1.50)	952.50	(0.84)
2-methylphenol	313.15	[15]	1017.80	(0.74)	1027.15	(0.77)	1024.25	(0.78)
2-methylphenol	323.15	[15]	1005.22	(0.61)	1017.96	(0.59)	1016.31	(0.59)
2-methylphenol	333.15	[15]	995.57	(0.73)	1009.01	(0.31)	1008.44	(0.69)
2-methylpropan-2-ol	298.15	[13]	831.30	(2.10)	840.02	(1.40)	833.30	(0.89)
2-methylpropan-2-ol	303.15	[13]	828.96	(0.67)	837.10	(1.40)	828.20	(1.20)
2-methylpropan-2-ol	308.15	[13]	824.00	(2.80)	833.48	(1.10)	823.16	(0.54)
2-methylpropan-2-ol	313.15	[13]	818.00	(0.97)	826.27	(0.81)	818.39	(0.54)
2-methylpropan-2-ol	323.15	[13]	807.50	(1.4)	819.31	(0.81)	812.01	(0.46)
2-methylpyridine	293.15	[13]	943.71	(0.27)	963.91	(0.20)	945.28	(0.47)
2-methylpyridine	313.15	[13]	922.32	(0.27)	943.95	(0.29)	927.47	(0.29)
2-methylpyridine	333.15	[13]	901.43	(0.30)	924.33	(0.42)	909.31	(0.38)
3-methylaniline	293.15	[13]	1004.61	(0.81)	1019.69	(0.71)	966.69	(0.34)
3-methylaniline	308.15	[13]	988.70	(0.66)	1003.83	(0.71)	953.46	(0.27)
3-methylbutyl acetate	293.15	[13]	897.52	(0.21)	907.70	(0.39)	884.25	(0.73)
3-methylbutyl acetate	298.15	[13]	896.54	(1.40)	904.43	(0.29)	881.47	(0.67)
3-methylbutyl acetate	313.15	[13]	878.44	(0.65)	889.81	(0.31)	867.77	(0.39)
3-methylbutyl acetate	333.15	[13]	857.88	(0.38)	870.67	(0.26)	850.68	(0.25)
3-methylphenol	273.15	[13]	1057.83	(1.60)	1064.58	(0.75)	1055.34	(1.18)
3-methylphenol	285.15	[13]	1048.91	(0.97)	1055.53	(0.64)	1046.15	(0.61)
3-methylphenol	288.65	[13]	1046.41	(0.73)	1054.62	(1.70)	1045.34	(1.99)
3-methylphenol	293.15	[13]	1041.27	(1.20)	1052.53	(1.10)	1044.72	(1.15)
3-methylphenol	298.15	[13]	1039.33	(0.96)	1047.54	(1.40)	1042.11	(1.09)
3-methylphenol	313.15	[13]	1025.77	(0.66)	1038.49	(1.30)	1037.74	(1.24)
3-methylphenol	337.15	[13]	1002.97	(1.00)	1017.63	(0.70)	1014.73	(0.94)
3-methylpyridine	293.09	[16]	938.54	(0.25)	959.65	(0.28)	939.24	(0.24)
3-methylpyridine	298.24	[16]	932.59	(0.40)	954.24	(0.55)	935.17	(0.48)
3-methylpyridine	303.20	[16]	927.11	(0.46)	949.27	(0.45)	931.52	(0.57)
3-methylpyridine	308.29	[16]	922.16	(0.13)	945.27	(0.20)	927.02	(0.19)
3-methylpyridine	313.20	[16]	916.96	(0.48)	939.75	(0.38)	922.30	(0.65)
4-chloro-1-ethyl-2-methylbenzimidazole	289.95	[14]	1139.76	(1.30)	1164.72	(1.30)	1144.43	(0.64)
4-chloro-1-methylimidazole	292.95	[14]	1271.77	(0.59)	1306.74	(0.57)	1271.86	(0.58)
4-methyl-1H-imidazole	287.45	[14]	1070.51	(0.86)	1082.25	(1.00)	1024.77	(0.65)
4-methyl-1H-imidazole	291.15	[14]	1066.20	(0.82)	1077.16	(0.54)	1021.98	(0.71)
4-methyl-1H-imidazole	323.15	[14]	1038.20	(0.46)	1056.71	(0.78)	999.32	(0.37)
4-methyl-1H-imidazole	333.15	[14]	1029.77	(0.29)	1049.40	(0.60)	994.00	(0.75)
4-methyl-1H-imidazole	343.15	[14]	1021.51	(0.26)	1041.44	(0.81)	986.80	(0.56)
4-methyl-1H-imidazole	383.15	[14]	987.07	(0.60)	1009.27	(0.28)	957.58	(0.13)
4-methyl-1H-imidazole	426.15	[14]	948.98	(0.53)	976.02	(0.27)	926.42	(0.32)
4-methylphenol	313.15	[15]	1005.64	(0.80)	1006.33	(0.55)	1006.08	(0.75)
4-methylphenol	323.15	[15]	997.97	(0.61)	997.50	(0.41)	998.77	(0.33)
4-methylphenol	333.15	[15]	987.49	(0.64)	987.25	(0.46)	988.31	(0.44)

4-methylpyridine	298.15	[17]	949.80	(0.27)	975.83	(0.55)	955.83	(0.25)
4-methylpyridine	308.15	[17]	939.01	(0.47)	967.08	(0.42)	946.94	(0.16)
4-methylpyridine	318.15	[17]	929.79	(0.34)	956.83	(0.58)	938.40	(0.59)
4-methylpyridine	328.15	[17]	919.06	(0.55)	946.41	(0.55)	929.50	(0.40)
4-methylpyridine	338.15	[17]	908.45	(0.57)	937.14	(0.61)	921.07	(0.26)
5-chloro-1-ethyl-2-methylbenzimidazole	292.95	[14]	1127.71	(0.37)	1145.97	(0.76)	1131.26	(1.30)
5-chloro-1-methylimidazole	293.65	[14]	1264.83	(0.43)	1291.99	(0.90)	1260.86	(0.76)
5-chloro-1-methylimidazole	290.85	[14]	1268.10	(0.57)	1295.87	(0.51)	1261.87	(0.96)
acetaldehyde	273.15	[13]	803.65	(0.33)	859.76	(0.42)	795.64	(0.42)
acetaldehyde	288.15	[18]	783.26	(0.59)	843.28	(0.33)	778.88	(0.21)
acetaldehyde	291.15	[13]	779.82	(0.58)	839.95	(0.48)	774.98	(0.48)
acetone	278.15	[19]	801.11	(0.31)	844.21	(0.22)	777.01	(0.35)
acetone	288.15	[19]	788.40	(0.15)	834.20	(0.26)	768.32	(0.37)
acetone	298.15	[19]	777.05	(0.41)	823.53	(0.18)	758.16	(0.25)
acetone	303.15	[20]	770.71	(0.28)	817.98	(0.27)	753.43	(0.39)
acetone	308.15	[19]	765.45	(0.45)	813.08	(0.35)	748.24	(0.24)
acetone	313.15	[20]	758.70	(0.38)	807.82	(0.41)	743.28	(0.26)
acetone	318.15	[19]	753.50	(0.35)	802.47	(0.22)	738.59	(0.40)
acetone	323.15	[13]	745.68	(0.36)	797.47	(0.30)	733.22	(0.37)
acetone	343.15	[13]	720.90	(0.70)	775.03	(0.60)	713.43	(0.27)
acetonitrile	288.15	[21]	797.32	(0.36)	842.39	(0.34)	776.30	(0.38)
acetonitrile	293.15	[21]	792.26	(0.13)	838.03	(0.24)	771.40	(0.07)
acetonitrile	298.15	[21]	787.87	(0.23)	833.98	(0.16)	766.86	(0.23)
acetonitrile	303.15	[21]	783.27	(0.25)	830.48	(0.13)	762.75	(0.21)
acetonitrile	308.15	[21]	778.37	(0.33)	826.43	(0.20)	758.15	(0.30)
acetonitrile	313.15	[21]	774.36	(0.12)	822.63	(0.21)	754.50	(0.28)
acetonitrile	318.15	[21]	769.42	(0.20)	818.55	(0.38)	749.82	(0.31)
acetonitrile	323.15	[21]	764.90	(0.18)	814.57	(0.44)	745.32	(0.09)
acetonitrile	328.15	[21]	760.34	(0.18)	810.33	(0.18)	740.99	(0.33)
acetonitrile	333.15	[21]	755.58	(0.17)	806.63	(0.26)	737.14	(0.30)
aniline	293.15	[13]	1024.00	(0.52)	1051.20	(0.74)	986.59	(0.41)
aniline	295.15	[13]	1020.89	(0.77)	1048.57	(1.40)	985.69	(0.87)
aniline	298.15	[13]	1018.46	(0.86)	1046.30	(0.91)	982.54	(0.44)
aniline	303.15	[13]	1013.00	(0.78)	1038.42	(0.65)	978.08	(0.39)
aniline	305.15	[13]	1009.04	(0.43)	1036.94	(0.58)	974.85	(0.54)
aniline	308.15	[13]	1005.24	(0.71)	1033.52	(0.66)	973.47	(0.43)
aniline	313.15	[13]	1000.16	(0.44)	1027.51	(0.64)	968.08	(0.44)
aniline	333.15	[13]	975.82	(0.51)	1003.04	(0.51)	948.11	(0.63)
anisole	288.15	[22]	990.56	(0.26)	999.81	(0.30)	979.81	(0.29)
anisole	293.15	[22]	984.69	(0.33)	995.02	(0.38)	973.94	(0.35)
anisole	298.15	[13]	978.47	(0.60)	989.37	(0.36)	968.44	(0.44)
anisole	303.15	[23]	973.60	(0.60)	984.10	(0.54)	963.57	(0.60)
anisole	308.15	[13]	968.42	(0.44)	978.99	(0.46)	957.86	(0.47)
anisole	318.15	[13]	957.44	(0.26)	967.34	(0.19)	948.17	(0.20)
anisole	323.15	[13]	951.26	(0.49)	962.28	(0.36)	942.52	(0.47)
anisole	348.15	[13]	922.96	(0.49)	934.80	(0.50)	915.12	(0.47)
anisole	353.15	[13]	917.11	(0.55)	929.54	(0.59)	910.34	(0.54)
benzene	293.15	[13]	857.76	(0.45)	869.38	(0.59)	880.66	(0.59)
benzene	298.15	[13]	851.62	(0.80)	861.91	(0.61)	874.37	(0.27)
benzene	303.15	[13]	844.82	(0.69)	854.03	(0.53)	868.57	(0.35)
benzene	308.15	[13]	837.67	(0.45)	847.35	(0.66)	862.57	(0.59)
benzene	313.15	[13]	828.47	(0.46)	840.66	(0.33)	856.63	(0.20)
benzene	318.15	[13]	822.05	(0.66)	834.24	(1.30)	851.06	(0.41)
benzene	323.15	[13]	815.84	(0.63)	826.18	(0.42)	844.79	(0.41)
benzene	343.15	[13]	785.42	(0.89)	798.02	(0.63)	820.50	(0.64)
benzonitrile	283.15	[24]	1034.31	(0.74)	1054.32	(0.73)	1014.09	(0.54)
benzonitrile	288.15	[24]	1030.74	(0.33)	1049.76	(0.49)	1010.42	(0.52)
benzonitrile	293.15	[13]	1025.62	(0.98)	1045.67	(0.49)	1008.03	(0.49)
benzonitrile	298.15	[13]	1021.81	(0.55)	1042.52	(0.74)	1004.88	(0.76)
benzonitrile	303.15	[13]	1016.69	(0.28)	1039.81	(0.33)	999.91	(0.34)
benzonitrile	308.15	[24]	1012.30	(0.37)	1034.44	(0.23)	995.19	(0.93)
benzonitrile	313.15	[24]	1006.72	(0.50)	1030.71	(0.62)	990.22	(0.47)
benzonitrile	318.15	[24]	1003.57	(0.32)	1026.11	(0.45)	986.24	(0.36)
benzonitrile	343.15	[13]	980.39	(0.36)	1005.50	(0.69)	965.92	(0.57)
benzoyl chloride	293.15	[13]	1203.39	(0.40)	1215.99	(0.39)	1179.39	(0.43)
benzoyl chloride	303.15	[13]	1192.90	(0.56)	1205.67	(0.30)	1169.94	(0.45)
benzoyl chloride	323.15	[13]	1171.06	(0.41)	1183.23	(0.69)	1148.01	(0.44)
bromobenzene	283.15	[13]	1501.60	(0.26)	1517.69	(0.52)	1520.15	(0.74)
bromobenzene	293.15	[13]	1485.99	(0.69)	1501.84	(0.47)	1507.09	(0.47)
bromobenzene	298.15	[13]	1477.60	(0.50)	1494.95	(0.45)	1499.11	(0.29)
bromobenzene	303.15	[25]	1471.36	(0.49)	1487.27	(0.60)	1491.50	(0.45)
bromobenzene	308.15	[25]	1462.79	(0.30)	1480.32	(0.57)	1485.03	(0.28)
bromobenzene	313.15	[13]	1455.99	(1.00)	1471.79	(0.80)	1477.35	(0.51)
bromobenzene	323.15	[13]	1438.63	(0.73)	1456.72	(0.47)	1463.38	(0.44)
bromobenzene	333.15	[13]	1422.75	(0.54)	1440.98	(0.61)	1448.50	(0.67)
bromobenzene	343.15	[13]	1408.16	(0.40)	1425.85	(0.62)	1434.61	(0.46)

bromoethane	183.15	[13]	1680.58	(0.76)	1708.39	(0.56)	1695.14	(0.47)
bromoethane	213.15	[13]	1616.55	(0.17)	1648.75	(0.39)	1639.74	(0.30)
bromoethane	233.15	[13]	1573.99	(0.65)	1607.52	(0.48)	1603.10	(0.33)
bromoethane	253.15	[13]	1529.62	(0.68)	1567.82	(0.52)	1566.59	(0.81)
bromoethane	273.15	[13]	1486.78	(0.70)	1527.75	(0.55)	1528.17	(0.63)
bromoethane	293.15	[13]	1440.81	(0.38)	1483.90	(0.29)	1492.18	(0.94)
bromoethane	313.15	[13]	1392.60	(1.30)	1442.25	(0.76)	1452.85	(0.68)
butan-1-ol	293.15	[13]	811.94	(0.71)	818.56	(0.25)	803.28	(0.34)
butan-1-ol	295.15	[13]	810.74	(0.45)	816.77	(0.66)	802.13	(0.46)
butan-1-ol	298.15	[13]	807.58	(0.52)	814.11	(0.54)	800.61	(0.60)
butan-1-ol	303.15	[13]	805.41	(0.81)	809.63	(0.55)	796.67	(0.35)
butan-1-ol	308.15	[13]	800.98	(0.74)	806.23	(0.87)	793.17	(0.23)
butan-1-ol	313.15	[13]	796.28	(0.32)	800.57	(0.84)	789.00	(0.46)
butan-1-ol	318.15	[13]	792.67	(0.52)	798.10	(0.59)	785.54	(0.38)
butan-1-ol	323.15	[13]	787.98	(0.13)	793.14	(0.64)	781.67	(0.59)
butan-1-ol	333.15	[13]	779.50	(0.30)	785.11	(0.22)	774.46	(0.39)
butan-1-ol	363.15	[13]	753.80	(0.45)	760.77	(0.55)	750.10	(0.21)
butan-2-one	293.15	[13]	778.15	(0.47)	809.58	(0.29)	763.82	(0.62)
butan-2-one	298.15	[13]	772.24	(0.17)	804.77	(0.29)	759.47	(0.48)
butan-2-one	303.15	[13]	767.47	(0.60)	799.75	(0.40)	755.20	(0.30)
butan-2-one	313.15	[13]	755.44	(0.36)	789.20	(0.22)	745.96	(0.43)
butan-2-one	318.15	[13]	750.06	(0.30)	784.50	(0.35)	740.62	(0.31)
butyl acetate	298.15	[13]	887.07	(0.35)	896.75	(0.37)	862.08	(0.40)
butyl acetate	313.15	[13]	871.87	(0.33)	882.16	(0.62)	847.15	(0.46)
butyl acetate	333.15	[13]	851.03	(0.30)	862.15	(0.43)	827.64	(0.52)
carbon tetrachloride	288.15	[13]	1589.08	(0.51)	1593.95	(0.41)	1638.23	(0.69)
carbon tetrachloride	298.15	[13]	1569.95	(0.55)	1574.03	(0.69)	1617.58	(0.58)
carbon tetrachloride	313.15	[13]	1541.93	(0.34)	1544.46	(0.76)	1588.23	(0.52)
carbon tetrachloride	323.15	[13]	1522.11	(0.93)	1524.46	(0.33)	1568.66	(0.63)
chlorobenzene	283.15	[13]	1103.63	(0.86)	1101.65	(0.65)	1112.18	(0.42)
chlorobenzene	293.15	[13]	1090.54	(0.98)	1089.13	(0.75)	1100.42	(0.47)
chlorobenzene	298.15	[13]	1084.94	(0.98)	1083.28	(0.30)	1095.55	(0.39)
chlorobenzene	303.15	[13]	1079.19	(0.52)	1076.98	(0.53)	1089.18	(0.38)
chlorobenzene	308.15	[25]	1071.65	(0.67)	1070.84	(0.38)	1083.50	(0.32)
chlorobenzene	313.15	[13]	1065.25	(0.51)	1063.62	(0.42)	1078.10	(0.53)
chlorobenzene	333.15	[13]	1039.88	(0.65)	1037.37	(0.28)	1054.66	(0.82)
cyclohexane	293.15	[13]	754.89	(0.39)	755.59	(0.33)	755.59	(0.33)
cyclohexane	298.15	[13]	750.24	(0.62)	750.46	(0.47)	750.46	(0.47)
cyclohexane	303.15	[13]	745.64	(0.52)	746.04	(0.56)	746.04	(0.56)
cyclohexane	308.15	[13]	740.18	(0.42)	739.81	(0.36)	739.81	(0.36)
cyclohexane	313.15	[13]	733.83	(0.37)	734.78	(0.51)	734.78	(0.51)
cyclohexane	318.15	[13]	728.94	(0.84)	730.14	(0.26)	730.14	(0.26)
cyclohexane	323.15	[13]	724.96	(0.25)	724.59	(0.68)	724.59	(0.68)
cyclohexane	333.15	[13]	713.48	(0.37)	713.47	(0.81)	713.47	(0.81)
cyclohexane	343.15	[13]	701.87	(0.27)	701.65	(0.65)	701.65	(0.65)
cyclopentane	295.15	[13]	714.93	(0.36)	716.37	(0.40)	716.37	(0.40)
cyclopentane	297.65	[13]	713.07	(0.32)	712.54	(0.72)	712.54	(0.72)
ethane-1,2-diol	273.15	[13]	1190.51	(1.00)	1212.00	(0.99)	1101.86	(0.66)
ethane-1,2-diol	293.15	[13]	1183.39	(1.30)	1204.09	(0.90)	1086.50	(0.49)
ethane-1,2-diol	295.15	[13]	1181.72	(1.20)	1202.00	(0.59)	1085.35	(0.80)
ethane-1,2-diol	298.15	[13]	1178.23	(2.00)	1199.44	(0.52)	1083.71	(0.72)
ethane-1,2-diol	303.15	[13]	1177.84	(1.80)	1197.94	(0.42)	1079.25	(0.51)
ethane-1,2-diol	323.15	[13]	1162.70	(1.30)	1191.14	(0.22)	1064.63	(0.40)
ethanol	288.15	[13]	809.93	(0.57)	814.66	(0.47)	783.41	(0.54)
ethanol	293.15	[13]	805.89	(0.46)	810.44	(0.40)	778.48	(0.59)
ethanol	298.15	[13]	801.06	(0.51)	805.35	(0.43)	774.13	(0.27)
ethanol	303.15	[13]	795.53	(0.32)	799.72	(0.46)	770.06	(0.43)
ethanol	308.15	[26]	790.79	(0.44)	795.43	(0.52)	765.41	(0.44)
ethanol	313.15	[27]	786.34	(0.55)	790.87	(0.24)	761.20	(0.50)
ethanol	318.15	[13]	781.84	(0.22)	786.12	(0.46)	756.07	(0.46)
ethanol	323.15	[27]	776.45	(0.49)	780.74	(0.35)	751.19	(0.60)
ethanol	333.15	[13]	766.20	(0.54)	771.57	(0.48)	742.65	(0.31)
ethoxybenzene	298.15	[13]	952.38	(0.27)	956.23	(0.31)	945.37	(0.33)
ethoxyethane	288.15	[13]	735.13	(0.59)	735.80	(0.24)	723.40	(0.31)
ethoxyethane	293.15	[13]	728.42	(0.29)	728.77	(0.38)	718.89	(0.60)
ethoxyethane	298.15	[13]	721.86	(0.50)	722.76	(0.40)	711.80	(0.29)
ethoxyethane	303.15	[13]	716.08	(0.39)	716.78	(0.39)	705.65	(0.50)
ethoxyethane	313.15	[13]	702.03	(0.20)	703.34	(0.69)	693.27	(0.24)
ethyl acetate	273.15	[13]	955.92	(0.40)	972.67	(0.81)	909.51	(0.56)
ethyl acetate	283.15	[13]	943.12	(0.38)	961.39	(0.38)	897.58	(0.21)
ethyl acetate	298.15	[13]	925.56	(0.49)	944.35	(0.39)	881.00	(0.23)
ethyl acetate	303.15	[13]	919.96	(0.34)	938.66	(0.37)	874.96	(0.55)
fluorobenzene	273.15	[13]	1004.11	(0.48)	1020.32	(0.31)	1018.27	(0.38)
fluorobenzene	293.15	[13]	974.62	(0.48)	992.64	(0.77)	994.19	(0.31)
fluorobenzene	313.15	[13]	945.15	(0.55)	964.15	(0.51)	969.48	(0.63)
fluorobenzene	333.15	[13]	914.56	(0.78)	936.25	(0.56)	944.81	(0.65)

fluorobenzene	353.15	[13]	882.11	(1.30)	906.08	(1.00)	919.54	(0.80)
formamide	278.15	[28]	1161.78	(0.27)	1239.04	(0.21)	1235.79	(0.24)
formamide	288.15	[28]	1148.76	(0.18)	1228.92	(0.46)	1223.54	(0.31)
formamide	298.15	[28]	1135.43	(0.43)	1218.61	(0.69)	1212.32	(0.21)
formamide	308.15	[28]	1123.20	(0.43)	1209.05	(0.44)	1201.02	(0.47)
formamide	313.15	[13]	1116.22	(0.33)	1203.73	(0.46)	1194.57	(0.14)
formamide	323.15	[28]	1103.05	(0.45)	1192.93	(0.28)	1183.75	(0.46)
formamide	333.15	[28]	1089.83	(0.45)	1182.28	(0.44)	1173.03	(0.49)
heptafluoro-2,3,3-trichlorobutane	273.15	[29]	1771.62	(0.60)	1772.67	(1.00)	1759.55	(1.20)
heptafluoro-2,3,3-trichlorobutane	298.05	[29]	1730.23	(1.60)	1728.84	(0.9)	1715.78	(0.89)
heptafluoro-2,3,3-trichlorobutane	334.35	[29]	1664.66	(0.98)	1665.12	(0.93)	1657.71	(0.71)
heptafluoro-2,3,3-trichlorobutane	343.65	[29]	1646.82	(0.71)	1647.55	(0.60)	1644.37	(1.70)
heptafluoro-2,3,3-trichlorobutane	362.95	[29]	1611.72	(1.30)	1610.86	(1.60)	1610.00	(1.20)
heptafluoro-2,3,3-trichlorobutane	371.45	[29]	1598.49	(1.10)	1597.84	(0.70)	1597.50	(1.30)
heptafluoro-2,3,3-trichlorobutane	390.65	[29]	1560.22	(1.30)	1561.34	(0.84)	1563.65	(1.20)
heptafluoro-2,3,3-trichlorobutane	273.15	[29]	1771.62	(0.60)	1772.67	(1.00)	1759.55	(1.20)
hex-1-ene	273.15	[30]	671.06	(0.42)	672.01	(0.28)	684.97	(0.19)
hex-1-ene	283.15	[31]	660.41	(0.34)	660.77	(0.23)	676.04	(0.27)
hex-1-ene	289.40	[31]	652.08	(0.08)	654.26	(0.43)	670.03	(0.31)
hex-1-ene	293.15	[30]	648.43	(0.33)	648.67	(0.35)	666.68	(0.23)
hex-1-ene	298.15	[31]	642.88	(0.34)	643.92	(0.30)	661.78	(0.25)
hex-1-ene	303.15	[32]	636.72	(0.51)	637.63	(0.34)	657.06	(0.35)
hex-1-ene	312.92	[32]	625.15	(0.26)	626.76	(0.42)	648.34	(0.44)
hex-1-ene	323.15	[31]	612.29	(0.34)	614.00	(0.80)	639.18	(0.40)
hex-1-ene	333.15	[30]	599.01	(0.81)	601.62	(0.71)	628.90	(0.16)
hexane	293.15	[13]	640.15	(0.46)	639.84	(0.48)	648.97	(0.33)
hexane	298.15	[13]	634.56	(0.34)	633.93	(0.48)	644.57	(0.27)
hexane	313.15	[13]	618.60	(0.46)	619.04	(0.37)	630.57	(0.49)
hexane	328.15	[13]	602.23	(0.28)	602.07	(0.23)	615.75	(0.20)
iodobenzene	293.15	[13]			1843.18	(0.49)	1845.58	(0.56)
iodobenzene	298.15	[13]			1834.94	(0.43)	1839.47	(0.68)
iodobenzene	313.15	[13]			1808.66	(0.66)	1814.90	(0.99)
iodobenzene	323.15	[13]			1792.33	(0.26)	1798.01	(0.84)
iodobenzene	333.15	[13]			1772.39	(0.56)	1782.31	(0.43)
iodobenzene	353.15	[13]			1736.64	(0.71)	1750.24	(0.89)
iodoethane	213.15	[13]			2139.69	(0.35)	2127.54	(0.85)
iodoethane	253.15	[13]			2035.86	(0.07)	2037.17	(0.82)
iodoethane	273.15	[13]			1984.38	(0.60)	1992.29	(0.74)
iodoethane	288.15	[13]			1945.15	(0.80)	1956.59	(0.59)
iodoethane	293.15	[13]			1930.73	(0.76)	1944.98	(0.67)
iodoethane	313.15	[13]			1876.59	(0.84)	1898.51	(0.68)
iodoethane	343.15	[13]			1791.53	(1.10)	1827.26	(0.55)
iodomethane	253.15	[13]			2376.11	(1.30)	2336.33	(0.20)
iodomethane	273.15	[13]			2304.95	(0.85)	2270.97	(0.90)
iodomethane	293.15	[13]			2231.74	(1.80)	2205.80	(1.00)
iodomethane	298.15	[13]			2212.14	(1.00)	2187.39	(0.55)
methanamine	273.15	[13]	808.39	(0.56)	828.28	(0.61)	685.66	(0.22)
methanamine	275.01	[33]	806.40	(0.80)	826.57	(0.47)	684.10	(0.26)
methanamine	284.14	[33]	795.19	(0.50)	815.50	(0.63)	674.22	(0.43)
methanamine	290.36	[33]	787.19	(0.25)	808.62	(0.47)	667.14	(0.43)
methanamine	292.93	[33]	783.94	(0.26)	805.91	(0.50)	664.16	(0.29)
methanol	288.15	[34]	825.04	(0.24)	840.33	(0.53)	759.65	(0.47)
methanol	293.15	[13]	819.22	(0.44)	836.70	(0.50)	755.00	(0.41)
methanol	298.15	[13]	813.69	(0.45)	830.22	(0.68)	749.93	(0.69)
methanol	303.15	[13]	809.07	(0.40)	825.69	(0.33)	745.72	(0.53)
methanol	308.15	[34]	803.63	(0.57)	820.62	(0.21)	740.78	(0.23)
methanol	313.15	[13]	798.08	(0.24)	816.32	(0.20)	736.30	(0.36)
methanol	318.15	[34]	791.24	(0.26)	811.00	(0.41)	731.36	(0.34)
methanol	323.15	[13]	787.86	(0.20)	805.17	(0.28)	726.42	(0.30)
methanol	328.15	[34]	781.78	(0.26)	800.72	(0.15)	721.38	(0.07)
methanol	333.15	[13]	776.53	(0.53)	794.76	(0.53)	716.51	(0.37)
methyl acetate	283.15	[13]	978.18	(0.40)	1001.70	(0.33)	917.40	(0.39)
methyl acetate	293.15	[13]	965.52	(0.54)	991.05	(0.50)	905.98	(0.48)
methyl acetate	298.15	[13]	959.60	(0.53)	984.88	(0.40)	899.26	(0.36)
methyl acetate	318.15	[13]	935.01	(0.43)	961.85	(0.44)	875.30	(0.34)
N,N-diethylformamide	298.15	[13]	896.86	(0.25)	920.98	(0.40)	888.37	(0.39)
N,N-dimethylacetamide	283.15	[35]	937.58	(0.30)	967.42	(0.48)	899.90	(0.20)
N,N-dimethylacetamide	298.15	[35]	922.62	(0.17)	953.82	(0.54)	887.93	(0.31)
N,N-dimethylacetamide	313.15	[35]	907.81	(0.06)	940.59	(0.46)	874.20	(0.23)
N,N-dimethylaniline	293.15	[13]	950.37	(0.37)	957.52	(0.44)	957.75	(0.46)
N,N-dimethylaniline	298.15	[13]	946.67	(0.28)	953.71	(0.20)	954.39	(0.43)
N,N-dimethylaniline	313.15	[13]	930.11	(0.49)	938.50	(0.15)	942.37	(0.41)
N,N-dimethylaniline	333.15	[13]	910.37	(0.16)	919.03	(0.35)	926.24	(0.43)
N,N-dimethylformamide	283.15	[36]	977.34	(0.23)	1014.66	(0.32)	947.19	(0.35)
N,N-dimethylformamide	288.15	[36]	972.03	(0.19)	1010.28	(0.24)	942.95	(0.13)
N,N-dimethylformamide	293.15	[36]	966.48	(0.24)	1005.90	(0.19)	938.78	(0.06)

N,N-dimethylformamide	298.15	[36]	962.44	(0.37)	1000.72	(0.22)	934.32	(0.19)
N,N-dimethylformamide	303.15	[36]	957.09	(0.23)	997.13	(0.32)	929.38	(0.17)
N,N-dimethylformamide	308.15	[36]	951.74	(0.24)	991.79	(0.34)	925.50	(0.17)
N,N-dimethylformamide	313.15	[36]	946.26	(0.42)	987.43	(0.39)	921.21	(0.34)
N,N-dimethylformamide	318.15	[36]	942.42	(0.49)	983.26	(0.22)	916.76	(0.26)
N,N-dimethylformamide	323.15	[36]	936.60	(0.25)	978.80	(0.33)	912.03	(0.40)
N,N-dimethylformamide	328.15	[36]	931.25	(0.32)	973.70	(0.31)	907.59	(0.55)
N,N-dimethylformamide	333.15	[36]	926.77	(0.22)	969.16	(0.26)	903.64	(0.33)
N,N-dimethylformamide	338.15	[36]	921.86	(0.25)	964.99	(0.48)	898.52	(0.37)
N,N-dimethylformamide	343.15	[36]	915.52	(0.12)	959.67	(0.29)	893.68	(0.45)
N,N-dimethylformamide	348.15	[36]	910.68	(0.32)	955.55	(0.46)	889.71	(0.30)
N,N-dimethylformamide	353.15	[36]	905.96	(0.23)	950.88	(0.36)	885.21	(0.70)
N-methylacetamide	303.15	[13]	973.34	(0.33)	1007.74	(1.70)	939.00	(0.32)
N-methylacetamide	308.15	[37]	968.79	(0.14)	1003.27	(0.92)	935.39	(0.51)
N-methylacetamide	313.15	[37]	964.91	(0.32)	999.19	(0.95)	930.57	(0.62)
N-methylacetamide	318.15	[37]	960.14	(0.32)	995.25	(0.76)	926.83	(0.47)
N-methylacetamide	323.15	[37]	955.47	(0.32)	990.58	(0.77)	923.58	(0.27)
N-methylacetamide	328.15	[37]	950.58	(0.51)	986.52	(0.54)	919.54	(0.41)
oxolane	295.15	[13]	891.09	(0.45)	897.89	(0.33)	877.86	(0.36)
oxolane	308.15	[13]	876.87	(0.44)	883.45	(0.48)	861.43	(0.45)
pent-1-ene	293.15	[13]	607.35	(0.37)	609.59	(0.70)	620.03	(0.70)
pentane	293.15	[13]	602.95	(0.20)	602.84	(0.28)	615.92	(0.25)
pentane	298.15	[13]	597.22	(0.44)	598.22	(0.15)	611.91	(0.41)
pentane	303.15	[13]	592.39	(0.25)	592.12	(0.49)	606.78	(0.26)
pentyl acetate	284.15	[13]	897.26	(0.22)	906.16	(0.40)	883.07	(0.35)
pentyl acetate	298.15	[13]	882.63	(0.49)	892.61	(0.46)	871.95	(0.29)
pentyl acetate	313.15	[13]	868.29	(0.39)	878.28	(0.68)	858.20	(0.41)
pentyl acetate	333.15	[13]	848.82	(0.44)	859.64	(0.48)	840.68	(0.40)
perfluorohexane	298.15	[13]	1687.24	(0.42)	1683.94	(1.30)	1652.10	(1.60)
perfluorohexane	308.15	[13]	1667.87	(0.96)	1666.36	(0.97)	1634.50	(0.89)
perfluorohexane	328.15	[13]	1628.47	(0.89)	1627.71	(1.60)	1599.74	(1.60)
phenol	313.15	[13]	1058.41	(0.62)	1076.29	(1.30)	1074.04	(1.16)
phenol	318.15	[38]	1051.45	(0.71)	1069.23	(0.66)	1067.55	(0.70)
phenol	323.15	[38]	1047.10	(0.61)	1063.93	(0.38)	1063.20	(0.31)
phenol	328.15	[38]	1042.52	(0.59)	1059.51	(0.77)	1057.98	(0.64)
phenol	333.15	[38]	1036.63	(0.89)	1053.66	(0.25)	1054.98	(0.63)
phenol	338.15	[38]	1030.21	(1.10)	1048.52	(0.53)	1049.79	(0.47)
phenol	343.15	[38]	1025.30	(0.99)	1044.31	(0.67)	1044.49	(0.80)
phenol	353.15	[13]	1012.83	(0.75)	1032.67	(0.92)	1037.01	(0.78)
piperidine	293.15	[13]	900.21	(1.60)	903.89	(0.42)	879.08	(0.59)
piperidine	298.15	[13]	895.39	(1.40)	902.30	(1.00)	874.83	(0.72)
piperidine	323.15	[13]	870.97	(0.45)	880.83	(0.87)	851.30	(0.43)
piperidine	348.15	[13]	847.16	(0.92)	856.96	(0.72)	828.68	(0.37)
piperidine	373.15	[13]	821.70	(0.57)	831.73	(0.83)	806.02	(0.46)
propan-2-ol	293.15	[13]	824.63	(0.54)	837.09	(0.76)	805.45	(0.59)
propan-2-ol	295.15	[13]	824.63	(0.65)	833.97	(0.80)	803.51	(0.65)
propan-2-ol	298.15	[13]	821.59	(0.61)	832.79	(0.72)	801.29	(0.87)
propan-2-ol	303.15	[13]	816.07	(0.57)	828.16	(0.76)	797.49	(0.71)
propan-2-ol	308.15	[13]	812.88	(0.43)	824.06	(0.67)	792.40	(0.37)
propan-2-ol	313.15	[13]	808.37	(0.63)	818.15	(0.50)	788.09	(0.67)
propan-2-ol	318.15	[13]	803.97	(0.44)	816.55	(0.58)	784.01	(0.57)
propan-2-ol	323.15	[13]	799.02	(0.77)	812.45	(0.50)	779.33	(0.80)
propan-2-ol	333.15	[13]	790.12	(0.62)	802.29	(0.31)	770.51	(0.59)
propan-2-ol	348.15	[13]	775.56	(0.61)	789.23	(0.78)	755.62	(0.62)
propyl acetate	273.15	[13]	930.60	(0.20)	947.72	(0.40)	902.39	(0.54)
propyl acetate	293.15	[13]	908.68	(0.32)	926.49	(0.73)	882.33	(0.24)
propyl acetate	298.15	[13]	903.60	(0.45)	920.02	(0.75)	876.56	(0.21)
propyl acetate	313.15	[13]	886.51	(0.35)	904.96	(0.49)	861.57	(0.28)
pyridine	293.15	[13]	985.52	(0.38)	1015.57	(0.30)	988.40	(0.76)
pyridine	298.15	[39]	978.99	(0.23)	1009.96	(0.35)	984.00	(0.70)
pyridine	303.15	[39]	973.54	(0.18)	1004.58	(0.83)	979.08	(0.33)
pyridine	308.15	[39]	968.31	(0.72)	999.52	(0.70)	974.13	(0.52)
pyridine	313.15	[13]	962.66	(0.43)	993.63	(0.60)	969.15	(0.65)
pyridine	323.15	[13]	950.14	(0.53)	982.74	(0.59)	959.01	(0.30)
pyridine	333.15	[13]	938.40	(0.45)	972.23	(0.61)	947.82	(0.38)
pyridine	343.15	[13]	925.75	(0.63)	961.75	(0.45)	937.77	(0.45)
pyridine	348.15	[13]	920.78	(0.73)	954.97	(0.57)	932.88	(0.49)
pyridine	368.15	[13]	896.12	(0.84)	932.94	(0.80)	912.75	(0.49)
pyridine	388.15	[13]	871.72	(1.10)	910.03	(0.44)	890.39	(0.31)
toluene	288.15	[13]	849.62	(0.62)	855.29	(0.43)	864.84	(0.37)
toluene	293.15	[13]	842.98	(0.68)	850.21	(0.49)	860.33	(0.25)
toluene	298.15	[13]	837.30	(0.46)	844.88	(0.76)	855.55	(0.40)
toluene	303.15	[13]	831.33	(0.07)	839.60	(0.34)	850.99	(0.20)
toluene	313.15	[13]	820.19	(0.56)	827.32	(0.41)	841.66	(0.28)
toluene	323.15	[13]	807.04	(0.48)	815.20	(0.57)	832.06	(0.38)
toluene	328.15	[13]	802.34	(0.58)	809.43	(0.41)	827.67	(0.41)

toluene	348.15	[13]	777.14	(0.76)	784.10	(0.36)	807.08	(0.40)
tribromomethane	283.15	[13]	2954.09	(1.30)	2999.32	(1.40)	2999.53	(2.00)
tribromomethane	293.15	[13]	2924.98	(1.40)	2965.34	(1.10)	2972.33	(0.89)
tribromomethane	303.15	[13]	2895.88	(0.93)	2937.27	(0.69)	2935.89	(1.20)
tribromomethane	313.15	[13]	2868.94	(0.42)	2906.59	(1.70)	2907.17	(0.76)
tribromomethane	343.15	[13]	2780.28	(0.52)	2819.47	(0.98)	2812.50	(1.70)
trichloromethane	293.15	[13]	1440.90	(0.17)	1451.03	(0.42)	1479.77	(1.00)
trichloromethane	295.15	[40]	1437.19	(0.72)	1446.19	(0.75)	1475.09	(0.43)
trichloromethane	297.15	[40]	1433.16	(0.64)	1443.24	(0.84)	1470.09	(0.45)
trichloromethane	298.15	[41]	1430.59	(0.58)	1440.14	(1.10)	1469.00	(1.50)
trichloromethane	303.15	[42]	1419.31	(0.47)	1430.21	(1.40)	1458.23	(0.73)
trichloromethane	308.15	[42]	1410.48	(1.00)	1418.84	(0.46)	1447.27	(0.68)
trichloromethane	313.15	[13]	1397.68	(1.90)	1409.74	(0.63)	1437.52	(0.91)
trichloromethane	323.15	[13]	1379.29	(0.80)	1388.49	(0.66)	1415.83	(1.10)

2.4. Validation I Data Set

Table 5: Simulation results for solvation free energies ΔG_{solv} in kJ/mol from the validation I data set. The first two columns mark the solute and solvent compounds, followed by the temperature T in K and the source for the experimental reference data. This is ensued by simulation results for the model parameter sets, whereas statistical uncertainties are given in brackets.

Solute	Solvent	T	Source	GAFF/RESP		GAFF/IPolQ-Mod		GAFF/IPolQ-Mod+LJ-Fit	
				ΔG_{solv}	$\delta\Delta G_{solv}$	ΔG_{solv}	$\delta\Delta G_{solv}$	ΔG_{solv}	$\delta\Delta G_{solv}$
1,1-difluoroethane	TIP3P	298.00	[1]	1.19	(0.08)	-2.15	(0.09)	-4.35	(0.10)
1,1-difluoroethane	TIP4P/2005	298.00	[1]	2.30	(0.09)	-0.78	(0.09)	-7.19	(0.14)
1,4-dichlorobenzene	ethoxyethane	298.00	[1]	-26.27	(0.04)	-26.72	(0.09)	-28.81	(0.10)
1,4-dioxane	2-methylpyridine	298.00	[1]	-26.01	(0.06)	-26.58	(0.14)	-20.68	(0.14)
1,4-dioxane	iodobenzene	298.00	[1]			-26.44	(0.09)	-20.21	(0.09)
1,4-dioxane	butanone	298.00	[1]	-25.54	(0.08)	-25.98	(0.09)	-20.44	(0.11)
1,4-dioxane	cyclohexanone	298.00	[1]	-26.03	(0.11)	-26.52	(0.24)	-19.83	(0.12)
1-bromopropane	TIP3P	298.00	[1]	-0.37	(0.07)	-3.41	(0.13)	-4.08	(0.13)
1-bromopropane	TIP4P/2005	298.00	[1]	0.56	(0.13)	-2.36	(0.13)	-4.03	(0.11)
1-butanol	iodobenzene	298.00	[1]			-20.91	(0.09)	-23.62	(0.15)
1-butene	octan-1-ol	298.00	[1]	-5.05	(0.21)	-4.91	(0.23)	-8.00	(0.31)
1-iodopropane	TIP3P	298.00	[1]			-1.02	(0.11)	-5.01	(0.08)
1-iodopropane	TIP4P/2005	298.00	[1]			0.02	(0.11)	-4.77	(0.19)
1-pentyne	octan-1-ol	298.00	[1]	-17.63	(0.19)	-18.74	(0.33)	-17.17	(0.35)
2,2-dimethylpropane	TIP3P	298.00	[1]	9.83	(0.05)	9.49	(0.07)	6.07	(0.11)
2,2-dimethylpropane	TIP4P/2005	298.00	[1]	10.65	(0.14)	10.90	(0.18)	5.88	(0.05)
2,6-dimethylpyridine	cyclohexane	298.00	[1]	-22.77	(0.08)	-22.85	(0.10)	-26.83	(0.05)
2-butanone	aniline	298.00	[1]	-20.21	(0.14)	-23.47	(0.23)	-21.76	(0.20)
2-butanone	1-butoxybutane	298.00	[1]	-15.14	(0.09)	-16.36	(0.09)	-16.97	(0.07)
2-butanone	dimethylacetamide	298.00	[1]	-18.19	(0.15)	-21.14	(0.16)	-20.97	(0.08)
2-butanone	triethylamine	298.00	[1]	-14.35	(0.08)	-14.97	(0.12)	-15.24	(0.12)
2-butanone	cyclohexanone	298.00	[1]	-17.93	(0.13)	-20.90	(0.17)	-21.23	(0.12)
2-methylaniline	benzene	298.00	[1]	-26.36	(0.09)	-26.44	(0.13)	-31.49	(0.14)
2-methylphenol	benzene	298.00	[1]	-27.88	(0.03)	-29.19	(0.13)	-33.36	(0.14)
2-methylpropene	carbon tetrachloride	298.00	[1]	-9.01	(0.08)	-8.61	(0.04)	-12.41	(0.07)
2-methylpyridine	benzene	298.00	[1]	-23.50	(0.08)	-24.24	(0.11)	-26.72	(0.10)
2-methylpyridine	2-methylpyridine	298.00	[1]	-23.58	(0.12)	-25.26	(0.19)	-27.16	(0.27)
2-pentanone	benzene	298.00	[1]	-21.64	(0.07)	-23.24	(0.07)	-24.25	(0.13)
2-pentanone	perfluorobenzene	298.00	[1]	-24.53	(0.11)	-26.62	(0.08)	-26.00	(0.06)
3-bromoprop-1-ene	octan-1-ol	298.00	[1]	-13.38	(0.38)	-12.67	(0.07)	-15.64	(0.36)
4-methylaniline	butylacetate	298.00	[1]	-31.90	(0.24)	-33.89	(0.15)	-34.64	(0.21)
4-methylphenol	1,2-dibromoethane	298.00	[1]	-30.30	(0.13)	-30.71	(0.12)	-35.74	(0.15)
4-methylphenol	cyclohexane	298.00	[1]	-21.39	(0.12)	-21.57	(0.08)	-25.97	(0.08)
4-methylphenol	iodobenzene	298.00	[1]			-28.39	(0.08)	-32.85	(0.19)
acetonitrile	heptane	298.00	[1]	-10.58	(0.07)	-10.63	(0.02)	-8.36	(0.06)
benzaldehyde	TIP3P	298.00	[1]	-16.07	(0.10)	-23.45	(0.10)	-14.89	(0.10)
benzaldehyde	TIP4P/2005	298.00	[1]	-15.34	(0.24)	-23.12	(0.12)	-15.47	(0.12)
benzamide	carbon tetrachloride	298.00	[1]	-28.85	(0.11)	-29.92	(0.07)	-31.67	(0.13)
benzamide	cyclohexane	298.00	[1]	-26.06	(0.11)	-26.21	(0.07)	-27.55	(0.14)
benzonitrile	heptane	298.00	[1]	-24.23	(0.03)	-24.28	(0.07)	-24.76	(0.09)
bromobenzene	heptane	298.00	[1]	-22.03	(0.07)	-22.11	(0.08)	-24.51	(0.03)
butylacetate	butylacetate	298.00	[1]	-27.61	(0.13)	-29.14	(0.1)	-27.79	(0.18)
chlorobenzene	chlorobenzene	298.00	[1]	-20.99	(0.15)	-21.47	(0.15)	-24.11	(0.10)
cis-1,2-dimethylcyclohexane	TIP3P	298.00	[1]	7.58	(0.03)	7.48	(0.13)	4.42	(0.08)
cis-1,2-dimethylcyclohexane	TIP4P/2005	298.00	[1]	8.97	(0.18)	8.81	(0.19)	4.99	(0.11)
cyclohexane	trichloromethane	298.00	[1]	-19.64	(0.06)	-19.59	(0.10)	-19.47	(0.06)
cyclohexane	benzene	298.00	[1]	-17.05	(0.05)	-16.53	(0.10)	-16.81	(0.10)
cyclopentane	octan-1-ol	298.00	[1]	-12.49	(0.18)	-12.54	(0.27)	-11.45	(0.32)
dichloro(difluoro)methane	trichloromethane	298.00	[1]	-10.31	(0.07)	-10.09	(0.07)	-10.54	(0.07)
dimethoxymethane	TIP3P	298.00	[1]	-7.58	(0.09)	-8.37	(0.11)	-11.13	(0.14)
dimethoxymethane	TIP4P/2005	298.00	[1]	-7.65	(0.07)	-8.64	(0.18)	-8.37	(0.20)
dimethylamine	TIP3P	298.00	[1]	-10.42	(0.09)	-11.60	(0.12)	-14.89	(0.05)
dimethylamine	TIP4P/2005	298.00	[1]	-12.66	(0.12)	-13.79	(0.19)	-14.57	(0.14)
ethanol	tribromomethane	298.00	[1]	-15.47	(0.22)	-15.41	(0.28)	-16.77	(0.05)
ethanol	aniline	298.00	[1]	-17.87	(0.13)	-19.26	(0.08)	-17.78	(0.15)
ethanol	triethylamine	298.00	[1]	-10.07	(0.15)	-10.05	(0.05)	-11.49	(0.08)
ethanol	butylacetate	298.00	[1]	-21.84	(0.11)	-24.89	(0.15)	-19.73	(0.17)
ethanol	pyridine	298.00	[1]	-19.27	(0.16)	-22.43	(0.10)	-20.83	(0.13)
ethanol	dimethylformamide	298.00	[1]	-24.33	(0.11)	-29.64	(0.10)	-22.82	(0.03)
ethanol	acetophenone	298.00	[1]	-19.49	(0.12)	-23.73	(0.27)	-19.24	(0.21)
ethene	octan-1-ol	298.00	[1]	2.23	(0.08)	1.91	(0.22)	1.72	(0.13)
ethoxyethane	cyclohexane	298.00	[1]	-13.75	(0.08)	-13.69	(0.07)	-13.98	(0.09)
ethylacetate	2-methylpropan-1-ol	298.00	[1]	-22.27	(0.27)	-24.52	(0.20)	-22.24	(0.20)
ethylacetate	perfluorobenzene	298.00	[1]	-25.09	(0.12)	-26.49	(0.11)	-22.61	(0.10)
ethyne	octan-1-ol	298.00	[1]	-8.79	(0.16)	-10.14	(0.22)	-6.76	(0.25)
fluorobenzene	fluorobenzene	298.00	[1]	-16.67	(0.06)	-17.50	(0.08)	-20.41	(0.03)

iodobenzene	octan-1-ol	298.00	[1]			-25.04	(0.31)	-28.61	(0.16)
iodobenzene	heptane	298.00	[1]			-24.10	(0.06)	-27.65	(0.11)
methylacetate	carbon tetrachloride	298.00	[1]	-16.20	(0.03)	-16.92	(0.09)	-14.23	(0.11)
N-(2-hydroxyphenyl)acetamide	TIP3P	298.00	[1]	-46.15	(0.13)	-55.73	(0.12)	-55.13	(0.11)
N-(2-hydroxyphenyl)acetamide	TIP4P/2005	298.00	[1]	-47.53	(0.13)	-57.96	(0.23)	-53.87	(0.12)
N-methylformamide	methylformamide	298.00	[1]	-33.13	(0.23)	-40.31	(0.38)	-36.46	(0.16)
octane	pyridine	298.00	[1]	-18.49	(0.13)	-14.37	(0.09)	-21.26	(0.05)
octane	dimethylacetamide	298.00	[1]	-15.85	(0.08)	-11.43	(0.11)	-19.56	(0.16)
octane	ethanol	298.00	[1]	-17.35	(0.08)	-16.50	(0.11)	-22.74	(0.07)
o-xylene	ethoxyethane	298.00	[1]	-21.99	(0.05)	-22.36	(0.07)	-27.37	(0.10)
1-propanol	iodobenzene	298.00	[1]			-17.74	(0.09)	-19.73	(0.14)
phenol	iodobenzene	298.00	[1]			-25.24	(0.12)	-28.74	(0.21)
phenol	butylacetate	298.00	[1]	-36.10	(0.22)	-38.26	(0.21)	-36.45	(0.14)
propionitrile	octan-1-ol	298.00	[1]	-16.62	(0.34)	-20.99	(0.29)	-19.69	(0.36)
propylacetate	cyclohexane	298.00	[1]	-20.14	(0.11)	-20.15	(0.10)	-19.55	(0.06)
propylamine	iodobenzene	298.00	[1]			-17.31	(0.14)	-19.56	(0.10)
propylamine	bromobenzene	298.00	[1]	-16.88	(0.15)	-17.61	(0.12)	-19.91	(0.21)
propyne	TIP3P	298.00	[1]	-4.27	(0.01)	-6.80	(0.08)	-1.47	(0.12)
propyne	TIP4P/2005	298.00	[1]	-3.97	(0.14)	-6.42	(0.15)	-1.86	(0.10)
pyridine	1-butoxybutane	298.00	[1]	-18.66	(0.06)	-19.32	(0.08)	-20.24	(0.20)
pyridine	butylacetate	298.00	[1]	-21.15	(0.11)	-22.67	(0.07)	-23.45	(0.10)
quinoline	trichloromethane	298.00	[1]	-37.87	(0.11)	-40.39	(0.11)	-42.89	(0.09)
tetrachloroethene	tetrachloroethene	298.00	[1]	-20.75	(0.09)	-20.74	(0.09)	-20.79	(0.08)
tetrafluoromethane	octan-1-ol	298.00	[1]	0.58	(0.11)	1.07	(0.12)	0.42	(0.20)
toluene	2-methylpyridine	298.00	[1]	-18.19	(0.08)	-18.42	(0.11)	-22.88	(0.16)
toluene	bromoethane	298.00	[1]	-19.85	(0.04)	-20.67	(0.09)	-24.79	(0.10)
toluene	1-butoxybutane	298.00	[1]	-17.89	(0.09)	-17.92	(0.11)	-22.36	(0.03)
toluene	anisole	298.00	[1]	-18.10	(0.06)	-18.43	(0.08)	-22.98	(0.07)
toluene	bromobenzene	298.00	[1]	-18.80	(0.08)	-19.38	(0.12)	-23.74	(0.07)
toluene	iodobenzene	298.00	[1]			-19.15	(0.11)	-23.68	(0.10)
toluene	fluorobenzene	298.00	[1]	-18.95	(0.08)	-19.45	(0.08)	-23.60	(0.10)
trans-2-pentene	carbon tetrachloride	298.00	[1]	-12.81	(0.03)	-12.84	(0.10)	-17.09	(0.09)
tribromomethane	pentane	298.00	[1]	-23.52	(0.02)	-23.72	(0.03)	-22.78	(0.04)
trichloroethene	cyclohexane	298.00	[1]	-16.96	(0.08)	-17.01	(0.06)	-17.35	(0.09)

2.5. Validation II Data Set

Table 6: Simulation results for solvation free energies ΔG_{solv} in kJ/mol from the validation II data set. The first two columns mark the solute and solvent compounds, followed by the temperature T in K and the source for the experimental reference data. This is ensued by simulation results for the model parameter sets, whereas statistical uncertainties are given in brackets.

Solute	Solvent	T	Source	GAFF/RESP		GAFF/IPolQ-Mod+LJ-Fit	
				ΔG_{solv}	$\delta\Delta G_{solv}$	ΔG_{solv}	$\delta\Delta G_{solv}$
haloperidol	1,4-dioxane	298.15	[43]	-94.46	(0.18)	-103.09	(0.20)
haloperidol	acetone	298.15	[43]	-89.84	(0.18)	-97.59	(0.18)
haloperidol	benzene	298.15	[43]	-84.63	(0.16)	-98.45	(0.18)
haloperidol	butyl acetate	298.15	[43]	-95.41	(0.17)	-102.96	(0.22)
haloperidol	trichloromethane	298.15	[43]	-91.43	(0.16)	-101.22	(0.20)
haloperidol	N,N-dimethylformamide	298.15	[43]	-91.86	(0.20)	-103.67	(0.16)
haloperidol	ethanol	298.15	[43]	-88.97	(0.17)	-98.71	(0.17)
haloperidol	ethyl acetate	298.15	[43]	-97.34	(0.19)	-101.30	(0.18)
haloperidol	glycerol	298.15	[43]	-148.04	(0.21)	-86.70	(0.16)
haloperidol	methanol	298.15	[43]	-87.41	(0.18)	-95.99	(0.15)
haloperidol	toluene	298.15	[43]	-85.37	(0.17)	-96.00	(0.16)
phenacetin	benzene	297.90	[43]	-50.97	(0.06)	-54.02	(0.03)
phenacetin	trichloromethane	298.15	[43]	-58.43	(0.13)	-61.57	(0.10)
phenacetin	cyclohexane	298.15	[43]	-39.15	(0.18)	-39.15	(0.13)
phenacetin	ethanol	298.25	[43]	-55.40	(0.16)	-63.84	(0.06)
phenacetin	ethyl acetate	298.35	[43]	-58.14	(0.16)	-60.70	(0.15)
phenacetin	hexane	298.15	[43]	-38.26	(0.06)	-39.62	(0.04)
phenacetin	methanol	298.18	[43]	-55.94	(0.22)	-65.71	(0.17)
phenacetin	octan-1-ol	298.15	[43]	-50.96	(0.32)	-58.31	(0.40)
phenacetin	oxolane	298.17	[43]	-56.94	(0.15)	-63.76	(0.24)
temazepam	acetone	298.15	[43]	-80.88	(0.17)	-84.50	(0.13)
temazepam	acetonitrile	298.15	[43]	-85.03	(0.14)	-84.67	(0.30)
temazepam	1-phenylethan-1-one	298.15	[43]	-81.99	(0.34)	-86.45	(0.38)
temazepam	benzene	298.15	[43]	-75.49	(0.11)	-81.77	(0.13)
temazepam	phenylmethanol	298.15	[43]	-88.90	(0.65)	-88.04	(0.85)
temazepam	cyclohexane	298.15	[43]	-63.27	(0.12)	-66.68	(0.13)
temazepam	dichloromethane	298.15	[43]	-81.35	(0.06)	-86.00	(0.09)
temazepam	N,N-dimethylformamide	298.15	[43]	-86.37	(0.08)	-87.04	(0.25)
temazepam	ethanol	298.15	[43]	-78.38	(0.43)	-82.15	(0.31)
temazepam	ethyl acetate	298.15	[43]	-83.59	(0.28)	-84.22	(0.20)
temazepam	formamide	298.15	[43]	-86.45	(0.28)	-75.68	(0.18)
temazepam	hexane	298.15	[43]	-59.68	(0.08)	-65.28	(0.10)
temazepam	methanol	298.15	[43]	-79.54	(0.35)	-83.46	(0.15)
temazepam	methyl acetate	298.15	[43]	-85.24	(0.26)	-83.76	(0.12)
temazepam	propan-1-ol	298.15	[43]	-75.87	(0.26)	-80.81	(0.51)
temazepam	oxolane	298.15	[43]	-81.64	(0.17)	-87.03	(0.17)
temazepam	toluene	298.15	[43]	-73.87	(0.21)	-80.64	(0.16)
trimethoprim	acetone	297.96	[43]	-88.80	(0.14)	-81.56	(0.17)
trimethoprim	butan-1-ol	298.46	[43]	-79.27	(0.41)	-80.08	(0.64)
trimethoprim	ethanol	297.95	[43]	-81.20	(0.56)	-81.73	(0.20)
trimethoprim	methanol	298.17	[43]	-83.84	(0.13)	-82.92	(0.10)
trimethoprim	oxolane	298.08	[43]	-87.45	(0.19)	-83.79	(0.29)

2.6. Validation III Data Set

Table 7: Simulation results for densities ρ in kg/m^3 from the validation III data set. The two columns mark the compounds of the binary mixture, followed by the mole fraction x_1, temperature T in K and the source for the experimental reference data. This is ensued by simulation results for the model parameter sets, whereas statistical uncertainties are given in brackets.

Compound 1	Compound 2	x_1	T	Source	GAFF/RESP		GAFF/IPolQ-Mod		GAFF/IPolQ-Mod+LJ-Fit	
					ρ	$\delta\rho$	ρ	$\delta\rho$	ρ	$\delta\rho$
1,1,1-trichloroethane	cyclohexane	0.251	298.15	[13]	883.72	(0.56)	883.57	(0.69)	890.46	(0.39)
1,1-dimethylbuta-1,3-diene	hexane	0.108	198.15	[13]	737.54	(0.07)	737.55	(0.23)	744.69	(0.20)
1,2-dichlorohexafluorocyclopentene	heptafluoro-2,3,3-trichlorobutane	0.527	298.15	[13]	1691.29	(0.91)	1692.16	(0.61)	1664.31	(0.83)
1-bromobutane	heptane	0.415	183.15	[13]	998.27	(0.45)	999.79	(0.63)	1003.14	(0.75)
1-bromobutane	heptane	0.415	283.15	[13]	878.94	(0.37)	881.12	(0.62)	901.32	(0.34)
1-bromobutane	heptane	0.415	363.15	[13]	778.08	(0.54)	781.95	(0.81)	817.37	(0.29)
1-iodobutane	heptane	0.546	193.15	[13]			1264.02	(0.53)	1269.56	(0.91)
1-iodobutane	heptane	0.546	273.15	[13]			1150.62	(0.57)	1168.54	(0.24)
1-iodobutane	heptane	0.546	353.15	[13]			1030.44	(0.39)	1069.55	(0.24)
2,2,2-trifluoroethanol	ethanol	0.489	298.15	[13]	1096.88	(0.17)	1119.07	(0.78)	1064.66	(0.41)
2,3-dimethylbuta-1,3-diene	hexane	0.332	298.15	[13]	645.58	(0.19)	645.59	(0.27)	666.52	(0.26)
2-iodo-2-methylpropane	carbon tetrachloride	0.522	293.15	[13]			1626.90	(0.46)	1659.61	(0.50)
2-iodobutane	carbon tetrachloride	0.460	293.15	[13]			1607.97	(0.53)	1637.46	(0.61)
2-methylaniline	ethanol	0.506	308.15	[13]	921.29	(0.47)	938.28	(0.37)	892.49	(0.42)
2-methylbuta-1,3-diene	2-methylbut-2-ene	0.507	293.15	[13]	621.69	(0.33)	630.58	(0.20)	655.22	(0.32)
2-propan-2-yloxypropane	methylcyclohexane	0.500	288.15	[44]	763.13	(0.29)	764.95	(0.33)	764.54	(0.24)
2-propan-2-yloxypropane	methylcyclohexane	0.500	298.15	[44]	752.07	(0.26)	754.53	(0.33)	754.91	(0.26)
2-propan-2-yloxypropane	methylcyclohexane	0.500	308.15	[44]	742.29	(0.24)	744.46	(0.49)	746.22	(0.19)
4-methylpyridine	TIP3P	0.500	338.15	[17]	912.53	(0.53)	936.66	(0.73)	935.86	(0.71)
4-methylpyridine	TIP4P/2005	0.500	338.15	[17]	925.40	(0.17)	957.99	(1.50)	940.1	(0.61)
acetamide	phenol	0.500	353.15	[13]	1024.70	(0.26)	1050.74	(0.89)	1026.99	(0.70)
acetamide	phenol	0.500	333.15	[13]	1043.60	(0.76)	1069.02	(0.69)	1043.5	(0.73)
acetone	methanol	0.298	363.15	[13]	721.20	(0.37)	760.70	(0.36)	688.51	(0.27)
acetone	chlorobenzene	0.491	273.15	[13]	987.11	(0.17)	1013.25	(0.27)	983.25	(0.31)
acetone	iodomethane	0.600	273.15	[13]			1404.82	(0.38)	1323.11	(0.71)
acetone	bromobenzene	0.499	273.15	[13]	1232.19	(0.33)	1263.31	(0.29)	1221.71	(0.35)
acetone	iodomethane	0.400	253.15	[13]			1744.93	(0.70)	1663.76	(0.43)
acetone	TIP3P	0.230	293.15	[45]	884.76	(0.49)	918.11	(0.14)	864.12	(0.43)
acetone	TIP3P	0.230	310.93	[45]	863.09	(0.40)	899.28	(0.26)	845.15	(0.19)
acetone	TIP3P	0.230	323.2	[45]	847.25	(0.38)	885.50	(0.44)	831.45	(0.31)
acetone	TIP4P/2005	0.230	293.15	[45]	905.57	(0.53)	936.65	(0.39)	880.30	(0.29)
acetone	TIP4P/2005	0.230	310.93	[45]	888.70	(0.34)	920.89	(0.20)	865.09	(0.11)
acetone	TIP4P/2005	0.230	323.2	[45]	876.10	(0.43)	910.53	(0.34)	854.02	(0.44)
acetonitrile	carbon tetrachloride	0.550	323.15	[13]	1221.98	(0.46)	1253.72	(0.70)	1225.68	(0.26)
acetonitrile	benzoyl chloride	0.547	323.15	[13]	1030.18	(0.22)	1062.78	(0.47)	1009.89	(0.16)
acetonitrile	2-methylbuta-1,3-diene	0.624	293.15	[13]	723.56	(0.53)	756.42	(0.43)	721.23	(0.28)
acetonitrile	pent-1-ene	0.631	293.15	[13]	705.75	(0.69)	727.35	(0.46)	701.53	(0.34)
acetonitrile	pent-2-ene	0.631	293.15	[13]	706.34	(0.24)	722.42	(0.48)	702.93	(0.12)
aniline	cyclohexane	0.509	333.15	[13]	820.61	(0.57)	825.38	(0.52)	815.86	(0.57)
aniline	methanol	0.290	313.15	[13]	904.30	(0.24)	932.63	(0.30)	853.01	(0.28)
aniline	ethoxybenzene	0.521	333.15	[13]	940.78	(0.40)	953.48	(0.42)	929.00	(0.16)
aniline	ethoxybenzene	0.521	353.15	[13]	916.52	(0.49)	930.56	(0.50)	909.03	(0.28)
anisole	benzene	0.500	323.15	[13]	892.47	(0.52)	904.15	(0.42)	898.42	(0.49)
benzonitrile	chloromethylbenzene	0.450	343.15	[13]	995.00	(0.47)	1016.28	(0.61)	1006.81	(0.33)
benzonitrile	benzene	0.556	298.15	[13]	955.56	(0.38)	972.55	(0.61)	950.01	(0.43)
bromobenzene	benzene	0.413	343.15	[13]	1066.46	(0.50)	1081.28	(0.51)	1096.46	(0.43)
bromobenzene	chlorobenzene	0.674	273.15	[13]	1385.05	(1.66)	1397.63	(0.71)	1402.03	(0.31)
butyl acetate	propyl acetate	0.400	333.15	[13]	859.16	(0.43)	874.60	(0.34)	835.45	(0.65)
cyclohexane	hexane	0.500	298.15	[13]	685.28	(0.47)	685.27	(0.36)	698.34	(0.33)
ethoxybenzene	iodoethane	0.607	273.15	[13]			1277.23	(0.29)	1266.36	(0.35)
ethoxyethane	trichloromethane	0.500	293.15	[13]	1063.42	(0.92)	1072.29	(0.78)	1076.93	(0.24)
ethoxyethane	tribromomethane	0.442	293.15	[13]	1905.62	(0.61)	1923.7	(0.70)	1912.84	(0.75)
ethoxyethane	ethanol	0.567	273.15	[46]	774.45	(0.31)	781.42	(0.26)	771.84	(0.24)
ethyl acetate	ethanol	0.343	273.15	[13]	884.48	(0.49)	900.97	(0.31)	852.72	(0.20)
ethyl acetate	ethanol	0.520	303.15	[13]	874.15	(0.32)	893.50	(0.97)	842.17	(0.28)
ethyl carbamate	ethoxyethane	0.357	293.15	[13]	864.74	(0.36)	871.63	(0.53)	864.79	(0.36)
ethyl carbamate	ethanol	0.341	293.15	[13]	956.76	(0.75)	974.77	(0.48)	933.5	(0.44)
fluorobenzene	benzene	0.446	353.15	[13]	822.25	(0.68)	838.95	(0.24)	860.15	(0.32)
fluorobenzene	carbon tetrachloride	0.502	293.15	[13]	1273.96	(0.42)	1285.65	(1.10)	1305.6	(0.21)
fluorobenzene	benzene	0.446	273.15	[13]	939.36	(0.15)	954.43	(0.29)	957.04	(0.62)
formamide	1,4-dioxane	0.679	313.15	[13]	1091.45	(0.81)	1130.77	(0.21)	1133.65	(0.58)
formamide	TIP3P	0.489	333.15	[28]	1037.34	(0.32)	1100.93	(0.25)	1075.97	(0.21)
formamide	TIP4P/2005	0.489	333.15	[28]	1056.90	(0.23)	1113.67	(0.14)	1103.82	(0.22)
formamide	pyridine	0.266	298.15	[13]	1005.69	(0.27)	1040.65	(0.38)	1016.21	(0.25)
formamide	methanol	0.498	298.15	[13]	981.09	(0.28)	1029.95	(0.29)	975.02	(0.16)
formamide	methanol	0.498	313.15	[13]	962.70	(0.38)	1013.63	(0.28)	958.22	(0.19)

hexafluoro-2-propanol	benzene	0.299	323.15	[13]	1041.91	(0.65)	1060.67	(0.43)	1056.24	(0.28)
hexane	2-methylbuta-1,3-diene	0.793	198.15	[13]	734.08	(0.23)	733.04	(0.20)	740.82	(0.27)
hexane	2-methylbuta-1,3-diene	0.793	248.15	[13]	684.18	(0.20)	682.77	(0.33)	698.78	(0.16)
iodobenzene	benzene	0.276	353.15	[13]			1082.73	(0.34)	1107.32	(0.41)
iodoethane	iodomethane	0.500	288.15	[13]			2080.62	(1.10)	2078.37	(0.94)
methyl acetate	benzene	0.504	318.15	[13]	877.17	(0.08)	895.11	(0.46)	859.83	(0.22)
morpholine	benzene	0.356	298.15	[13]	940.68	(0.52)	951.37	(0.63)	942.53	(0.27)
N,N-dimethylaniline	Isopropylbenzol	0.600	333.15	[13]	865.81	(0.46)	871.84	(0.26)	884.74	(0.29)
N,N-dimethylaniline	phenol	0.437	323.15	[13]	978.18	(0.47)	989.17	(0.37)	992.44	(0.71)
N,N-dimethylaniline	phenol	0.437	373.15	[13]	924.91	(0.47)	936.75	(0.66)	947.76	(0.19)
N,N-dimethylformamide	2-methylbuta-1,3-diene	0.482	293.15	[13]	784.74	(0.55)	810.15	(0.82)	786.77	(0.26)
N-methylacetamide	propyl acetate	0.484	308.15	[47]	923.16	(0.15)	946.42	(0.25)	894.86	(0.35)
N-methylacetamide	propan-1-ol	0.516	303.15	[13]	892.35	(0.26)	916.12	(0.38)	866.72	(0.21)
N-phenylaniline	phenol	0.326	313.15	[13]	1057.31	(0.65)	1073.40	(0.65)	1068.71	(1.10)
oxolane	cyclohexane	0.534	295.15	[13]	809.77	(0.25)	809.74	(0.18)	797.66	(0.29)
perfluorohexane	hexane	0.485	298.15	[13]	1246.58	(0.63)	1246.23	(0.38)	1245.74	(0.51)
perfluorohexane	hexane	0.485	328.15	[13]	1195.77	(0.33)	1196.28	(0.95)	1202.92	(0.87)
phenol	benzene	0.455	343.15	[13]	900.81	(0.38)	912.02	(0.93)	924.50	(0.66)
phenol	methanol	0.254	313.15	[13]	915.05	(0.41)	914.98	(0.17)	878.52	(0.32)
phenylhydrazine	phenol	0.466	323.15	[13]	1059.86	(0.43)	1083.13	(0.64)	1040.52	(0.22)
piperidine	phenol	0.550	373.15	[13]	902.72	(0.16)	917.18	(0.70)	900.34	(0.53)
pyridine	piperidine	0.472	323.15	[13]	905.96	(0.52)	921.85	(0.43)	899.04	(0.28)
pyridine	2-chlorophenol	0.605	273.15	[13]	1135.68	(0.29)	1155.03	(0.68)	1138.03	(0.73)
pyridine	2-chlorophenol	0.605	333.15	[13]	1063.28	(0.63)	1087.71	(0.25)	1076.73	(0.22)
pyridine	2-chlorophenol	0.605	383.15	[13]	1001	(0.37)	1028.38	(0.69)	1022.2	(0.11)
quinoline	phenol	0.328	313.15	[13]	1066.75	(0.24)	1085.46	(0.53)	1077.77	(0.44)
quinoline	phenol	0.328	353.15	[13]	1026.57	(0.66)	1048.20	(0.38)	1043.67	(0.33)
tetrachloroethene	cyclopentane	0.486	298.15	[13]	1149.34	(0.33)	1152.21	(0.38)	1164.97	(0.47)
TIP3P	ethane-1,2-diamine	0.503	353.15	[48]	1103.27	(1.50)	1141.70	(0.76)	953.4	(0.35)
TIP3P	acetonitrile	0.496	333.15	[49]	796.21	(1.05)	841.44	(0.28)	764.86	(0.88)
TIP4P/2005	ethane-1,2-diamine	0.503	353.15	[48]	1115	(1.06)	1135.62	(0.44)	964.80	(0.28)
TIP4P/2005	acetonitrile	0.496	333.15	[49]	810.32	(0.22)	853.12	(0.23)	779.73	(0.58)
toluene	cyclohexane	0.500	328.15	[13]	751.72	(0.69)	753.77	(0.60)	765.44	(0.24)
tribromomethane	cyclohexane	0.390	293.15	[13]	1484.37	(0.54)	1488.24	(1.40)	1485.65	(0.44)

3. Representation of Force Field Accuracies for the Validation I Data Set

In order to further analyze the reasons for the unexpected performances of the three model parameter sets in the validation I data set, all solvation free energy systems from the refitting- and validation I data set were considered as hypothetical basis population (BP). For GAFF/IPolQ-Mod and GAFF/IPolQ-Mod+LJ-Fit, this consists of 457 ΔG_{solv} systems each, whereas for GAFF/RESP only 428 systems are considered due to the exclusion of iodine components. Thus, the question arises whether the validation I data set represents the hypothetical population statistically. In order to show the relative frequency of RMSD deviations, 1e6 RMSD values were calculated for each of the model parameter sets and each RMSD value refers to 100 randomly drawn ΔG_{solv} results from the basis population. The distribution of the RMSD values is shown in Figure .

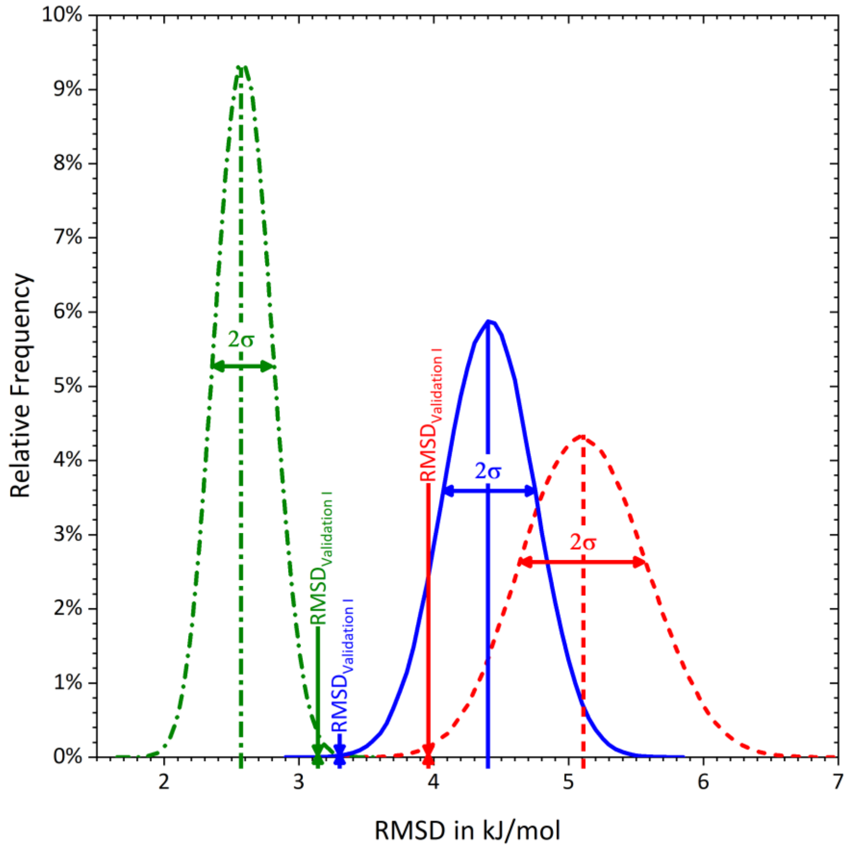

Figure 2. Relative frequencies of RMSD deviations calculated for 1e6 blocks of 100 randomly drawn ΔG_{solv}-results from refitting and validation I data sets. The blue continuous line refers to GAFF/RESP, the red dashed line to GAFF/IPolQ-Mod and the green dash dotted line to GAFF/IPolQ-Mod+LJ-Fit.

The figure illustrates that the peaks of the curves reflect the RMSD deviations for the population with $RMSD_{BP,RESP}$ = 4.40 kJ/mol, $RMSD_{BP,IPolQ\text{-}Mod}$ = 5.11 kJ/mol and $RMSD_{BP,IPolQ\text{-}Mod+LJ\text{-}Fit}$ = 2.57 kJ/mol. GAFF/IPolQ-Mod has the highest standard deviation with $\sigma_{BP,IPolQ\text{-}Mod}$ = 0.45 kJ/mol, followed by GAFF/RESP with $\sigma_{BP,RESP}$ = 0.34 kJ/mol and GAFF/IPolQ-Mod+LJ-Fit with $\sigma_{BP,IPolQ\text{-}Mod+LJ\text{-}Fit}$ = 0.21 kJ/mol. All RMSD deviations from the validation I data set deviate more than the standard deviation from the RMSD value of the population. As a result, the RMSD values from the validation I data set for GAFF/RESP and GAFF/IPolQ-Mod are significantly underestimated with respect to the overall basis population, while the RMSD value for GAFF/IPolQ-Mod+LJ fit is overestimated. We therefore conclude that the qualities of the ΔG_{solv} predictions for the validation I data set do not represent these of the overall population for neither of the three model parameter sets. As a consequence, we added the validation II data set for further analysis, which is discussed in the main paper.

4. References

[1] A. V. Marenich, C. P. Kelly, J. D. Thompson, G. D. Hawkins, C. C. Chambers, D. J. Giesen, P. Winget, C. J. Cramer, D. G. Truhlar. Minnesota Solvation Database – version 2012, University of Minnesota, Minneapolis.

[2] D. L. Mobley, J. P. Guthrie. FreeSolv: a database of experimental and calculated hydration free energies, with input files. *J Comput Aided Mol Des* **28** no. 7 (2014) 711–720.

[3] G. Duarte Ramos Matos, D. Y. Kyu, H. H. Loeffler, J. D. Chodera, M. R. Shirts, D. L. Mobley. Approaches for Calculating Solvation Free Energies and Enthalpies Demonstrated with an Update of the FreeSolv Database. *J. Chem. Eng. Data* (2017).

[4] A. Villa, A. E. Mark. Calculation of the free energy of solvation for neutral analogs of amino acid side chains. *J. Comput. Chem.* **23** no. 5 (2002) 548–553.

[5] A. Mecklenfeld, G. Raabe. *Efficient Molecular Simulations of the Free Energy of Solvation - Jahrestreffen der ProcessNet-Fachgruppe Molekulare Modellierung*, Frankfurt/Main, Germany 03/09/2017.

[6] GROMACS development team. Force fields in GROMACS, http://manual.gromacs.org/documentation/current/user-guide/force-fields.html?highlight=force%20fields%20gromacs.

[7] K. Vanommeslaeghe, A. D. MacKerell, JR. Automation of the CHARMM General Force Field (CGenFF) I: bond perception and atom typing. *J. Chem. Inf. Model.* **52** no. 12 (2012) 3144–3154.

[8] K. Vanommeslaeghe, E. P. Raman, A. D. MacKerell, JR. Automation of the CHARMM General Force Field (CGenFF) II: assignment of bonded parameters and partial atomic charges. *J. Chem. Inf. Model.* **52** no. 12 (2012) 3155–3168.

[9] E. Prabhu Raman. *cgenff_charmm2gmx.py* 2014.

[10] B. D. Ray. *topolbuild*, IUPUI Physics Dept., Indianapolis, IN 46202, USA 2009.

[11] R. Wolfenden, L. Andersson, P. M. Cullis, C. C. B. Southgate. Affinities of amino acid side chains for solvent water. *Biochemistry* **20** no. 4 (1981) 849–855.

[12] M. T. Geballe, A. G. Skillman, A. Nicholls, J. P. Guthrie, P. J. Taylor. The SAMPL2 blind prediction challenge: introduction and overview. *J Comput Aided Mol Des* **24** no. 4 259–279.

[13] H. Landolt, K.-H. Hellwege, O. Madelung. *Zahlenwerte und Funktionen aus Naturwissenschaften und Technik*, Springer, Berlin 1974.

[14] K. Hofmann. *The Chemistry of Heterocyclic Compounds, Imidazole and Its Derivatives*, John Wiley & Sons, Hoboken 1953.

[15] R. Rosal, I. Medina, E. Forster, J. MacInnes. Viscosities and densities for binary mixtures of cresols. *Fluid Phase Equilibria* **211** no. 2 (2003) 143–150.

[16] W. Marczak. Speed of Ultrasound, Density, and Adiabatic Compressibility for 3-Methylpyridine + Heavy Water in the Temperature Range 293−313 K. *J. Chem. Eng. Data* **41** no. 6 (1996) 1462–1465.

[17] L.-C. Wang, H. Ding, J.-H. Zhao, C.-Y. Song, J.-S. Wang. Density and Viscosity of (4-Picoline + Water) Binary Mixtures from T = (298.15 to 338.15) K. *J. Chem. Eng. Data* **54** no. 3 (2009) 1000–1003.

[18] D. Zhu, D. Gao, H. Zhang, B. Winter, P. Lücking, H. Sun, H. Guan, H. Chen, J. Shi. Geometric Structures of Associating Component Optimized toward Correlation and Prediction of Isobaric Vapor–Liquid Equilibria for Binary and Ternary Mixtures of Ethanal, Ethanol, and Ethanoic Acid. *J. Chem. Eng. Data* **58** no. 1 (2013) 7–17.

[19] E. Vercher, F. J. Llopis, M. V. González-Alfaro, A. Martínez-Andreu. Density, Speed of Sound, and Refractive Index of 1-Ethyl-3-methylimidazolium Trifluoromethanesulfonate with Acetone, Methyl Acetate, and Ethyl Acetate at Temperatures from (278.15 to 328.15) K. *J. Chem. Eng. Data* **55** no. 3 (2010) 1377–1388.

[20] N. Deenadayalu, P. Bhujrajh. Density, Speed of Sound, and Derived Thermodynamic Properties of Ionic Liquids [EMIM] + [BETI] − or ([EMIM] + [CH 3 (OCH 2 CH 2) 2 OSO 3] − + Methanol or + Acetone) at T = (298.15 or 303.15 or 313.15) K. *J. Chem. Eng. Data* **53** no. 5 (2008) 1098–1102.

[21] M. T. Khimenko, N. N. Gritsenko. Determination of the Polarisabilities and Radii of the Acetonitrile and Dimethylacetamide Molecules. *Zh. Fiz. Khim.* **54** (1980) 198–199.

[22] J. A. Al-Kandary, A. S. Al-Jimaz, A.-H. M. Abdul-Latif. Viscosities, Densities, and Speeds of Sound of Binary Mixtures of Benzene, Toluene, o -Xylene, m -Xylene, p -Xylene, and Mesitylene with Anisole at (288.15, 293.15, 298.15, and 303.15) K. *J. Chem. Eng. Data* **51** no. 6 (2006) 2074–2082.

[23] J. N. Nayak, M. I. Aralaguppi, T. M. Aminabhavi. Density, Viscosity, Refractive Index, and Speed of Sound in the Binary Mixtures of Ethyl Chloroacetate with Aromatic Liquids at 298.15, 303.15, and 308.15 K. *J. Chem. Eng. Data* **47** no. 4 (2002) 964–969.

[24] Y. Lei, Z. Chen, X. An, M. Huang, W. Shen. Measurements of Density and Heat Capacity for Binary Mixtures { x Benzonitrile + (1 − x) (Octane or Nonane)} †. *J. Chem. Eng. Data* **55** no. 10 (2010) 4154–4161.

[25] J. N. Nayak, M. I. Aralaguppi, T. M. Aminabhavi. Density, Viscosity, Refractive Index, and Speed of Sound in the Binary Mixtures of Ethyl Chloroacetate + Cyclohexanone, + Chlorobenzene, + Bromobenzene, or + Benzyl Alcohol at (298.15, 303.15, and 308.15) K. *J. Chem. Eng. Data* **48** no. 3 (2003) 628–631.

[26] A. García-Abuín, D. Gómez-Díaz, M. D. La Rubia, J. M. Navaza, R. Pacheco. Density, Speed of Sound, and Isentropic Compressibility of Triethanolamine (or N -Methyldiethanolamine) + Water + Ethanol Solutions from t = (15 to 50) °C. *J. Chem. Eng. Data* **54** no. 11 (2009) 3114–3117.

[27] G. Sivaramprasad, M. V. Rao, D. H. L. Prasad. Density and viscosity of ethanol + 1,2-dichloroethane, ethanol + 1,1,1-trichloroethane, and ethanol + 1,1,2,2-tetrachloroethane binary mixtures. *J. Chem. Eng. Data* **35** no. 2 (1990) 122–124.

[28] G. I. Egorov, D. M. Makarov. Densities and Molar Isobaric Thermal Expansions of the Water + Formamide Mixture over the Temperature Range from 274.15 to 333.15 K at Atmospheric Pressure. *J. Chem. Eng. Data* **62** no. 4 (2017) 1247–1256.

[29] R. H. Capps, W. M. Jackson. Density, Vapor Pressure And Heat Of Vaporization Of 2,2,3-Trichloro-Heptafluorobutane. *J. Phys. Chem.* **60** no. 6 (1956) 811–812.

[30] G. A. Torín-Ollarves, J. J. Segovia, M. C. Martín, M. A. Villamañán. Density, Viscosity, and Isobaric Heat Capacity of the Mixture (1-Butanol + 1-Hexene). *J. Chem. Eng. Data* **58** no. 10 (2013) 2717–2723.

[31] A. M. Kerimov, T. A. Apaev. Experimental values of density of 1-hexene,1-octene,cyclohexene,cyclohexane, and methylcyclohexane in dependence on temperature and pressure. *Teplofiz.Svoistva Vesh.Mater.* (1972) 26–46.

[32] D. I. Sagdeev, M. G. Fomina, G. K. Mukhamedzyanov, I. M. Abdulagatov. Experimental Study and Correlation Models of the Density and Viscosity of 1-Hexene and 1-Heptene at Temperatures from (298 to 473) K and Pressures up to 245 MPa. *J. Chem. Eng. Data* **59** no. 4 (2014) 1105–1119.

[33] W. A. Felsing, A. R. Thomas. Vapor Pressures and Other Physical Constants of Methylamine and Methylamine Solutions. *Ind. Eng. Chem.* **21** no. 12 (1929) 1269–1272.

[34] M. T. Zafarani-Moattar, N. Tohidifar. Vapor−Liquid Equilibria, Density, and Speed of Sound for the System Poly(ethylene glycol) 400 + Methanol at Different Temperatures. *J. Chem. Eng. Data* **51** no. 5 (2006) 1769–1774.

[35] S. Mrad, C. Lafuente, M. Hichri, I. Khattech. Density, Speed of Sound, Refractive Index, and Viscosity of the Binary Mixtures of N, N -dimethylacetamide with Methanol and Ethanol. *J. Chem. Eng. Data* **61** no. 9 (2016) 2946–2953.

[36] J. M. Bernal-García, A. Guzmán-López, A. Cabrales-Torres, A. Estrada-Baltazar, G. A. Iglesias-Silva. Densities and Viscosities of (N, N -Dimethylformamide + Water) at Atmospheric Pressure from (283.15 to 353.15) K. *J. Chem. Eng. Data* **53** no. 4 (2008) 1024–1027.

[37] A. A. Dyshin, O. V. Eliseeva, M. G. Kiselev. Density and Viscosity of N -Methylacetamide–Calcium Chloride Mixtures over the Temperature Range from 308.15 to 328.15 K at Atmospheric Pressure. *J. Chem. Eng. Data* **62** no. 12 (2017) 4128–4132.

[38] D. L. Cunha, J. A. P. Coutinho, J. L. Daridon, R. A. Reis, M. L. L. Paredes. Experimental Densities and Speeds of Sound of Substituted Phenols and Their Modeling with the Prigogine–Flory–Patterson Model. *J. Chem. Eng. Data* **58** no. 11 (2013) 2925–2931.

[39] J. N. Nayak, M. I. Aralaguppi, U. S. Toti, T. M. Aminabhavi. Density, Viscosity, Refractive Index, and Speed of Sound in the Binary Mixtures of Tri- n -butylamine + Triethylamine, + Tetrahydrofuran, + Tetradecane, + Tetrachloroethylene, + Pyridine, or + Trichloroethylene at (298.15, 303.15, and 308.15) K. *J. Chem. Eng. Data* **48** no. 6 (2003) 1483–1488.

[40] M. A. Varfolomeev, I. T. Rakipov, B. N. Solomonov, W. Marczak. Speed of Sound, Density, and Related Thermodynamic Excess Properties of Binary Mixtures of 2-Pyrrolidone and N -Methyl-2-pyrrolidone with Acetonitrile and Chloroform. *J. Chem. Eng. Data* **61** no. 3 (2016) 1032–1046.

[41] M. A. Varfolomeev, K. V. Zaitseva, I. T. Rakipov, B. N. Solomonov, W. Marczak. Speed of Sound, Density, and Related Thermodynamic Excess Properties of Binary Mixtures of Butan-2-one with C1–C4 n -Alkanols and Chloroform. *J. Chem. Eng. Data* **59** no. 12 (2014) 4118–4132.

[42] T. M. Aminabhavi, K. Banerjee. Density, Viscosity, Refractive Index, and Speed of Sound in Binary Mixtures of Dimethyl Carbonate with Methanol, Chloroform, Carbon Tetrachloride, Cyclohexane, and Dichloromethane in the Temperature Interval (298.15−308.15) K. *J. Chem. Eng. Data* **43** no. 6 (1998) 1096–1101.

[43] A. Jouyban. *Handbook of solubility data for pharmaceuticals*, CRC Press, Taylor & Francis Group, Boca Raton, Fla. 2010.

[44] J.-D. Ye, C.-H. Tu. Densities, Viscosities, and Refractive Indices for Binary and Ternary Mixtures of Diisopropyl Ether, Ethanol, and Methylcyclohexane. *J. Chem. Eng. Data* **50** no. 3 (2005) 1060–1067.

[45] K. T. Thomas, R. A. McAllister. Densities of liquid-acetone-water solutions up to their normal boiling points. *AIChE J.* **3** no. 2 (1957) 161–164.

[46] T. Nitta, J. Fujio, T. Katayama. Solubilities of nitrogen in binary solutions. Mixtures of ethanol with benzene, ethyl acetate, and diethyl ether. *J. Chem. Eng. Data* **23** no. 2 (1978) 157–159.

[47] V. Jaana, S. Nallani. THERMODYNAMIC AND TRANSPORT PROPERTIES OF BINARY LIQUID MIXTURES OF N-METHYLACETAMIDE WITH ALKYL (METHYL, ETHYL, n-PROPYL AND n-BUTYL) ACETATES AT 308.15 K. *Rasayan J. Chem* **1** no. 3 (2008) 602–608.

[48] Å. U. Burman, K. H. U. Ström. Density for (Water + Ethylenediamine) at Temperatures between (283 and 353) K. *J. Chem. Eng. Data* **53** no. 10 (2008) 2307–2310.

[49] H. A. Zarei, M. Z. Lavasani, H. Iloukhani. Densities and Volumetric Properties of Binary and Ternary Liquid Mixtures of Water (1) + Acetonitrile (2) + Dimethyl Sulfoxide (3) at Temperatures from (293.15 to 333.15) K and at Ambient Pressure (81.5 kPa). *J. Chem. Eng. Data* **53** no. 2 (2008) 578–585.
